# MultistageOT: Multistage optimal transport infers trajectories from a snapshot of single-cell data

**DOI:** 10.1073/pnas.2516046122

**Published:** 2025-12-11

**Authors:** Magnus Tronstad, Johan Karlsson, Joakim S. Dahlin

**Affiliations:** ^a^Department of Medicine Solna, Karolinska Institutet, and Center for Molecular Medicine, Karolinska University Hospital, Stockholm 171 76, Sweden; ^b^Department of Mathematics, KTH Royal Institute of Technology, Stockholm 100 44, Sweden

**Keywords:** trajectory inference, cell differentiation, single-cell, optimal transport, multistage

## Abstract

Single-cell RNA-sequencing captures a temporal slice, or a snapshot, of a cell differentiation process. A major bioinformatical challenge is the inference of differentiation trajectories from a single snapshot, and methods that account for outlier cells that are unrelated to the differentiation process have yet to be established. We present MultistageOT (https://github.com/dahlinlab/MultistageOT), a generalized optimal transport-based framework that models cell differentiation in a single snapshot as a series of intermediate cell transitions. MultistageOT employs multiple transport stages to establish temporal progression within the snapshot—overcoming limitations with the classic bimarginal formulation of optimal transport. Moreover, our multistage framework uses global information across all cells and differentiation stages to infer coherent trajectories from initial to terminal states. This allows MultistageOT to infer individual outlier cells that are unrelated to the analyzed differentiation process—an essential mechanism for preventing the inference of spurious or biologically implausible trajectories. We benchmark MultistageOT on snapshot data of cell differentiation, showing significantly improved fate prediction accuracy over state-of-the-art bimarginal optimal transport and demonstrating MultistageOT’s unique ability to detect outlier cells.

## Significance

Cell differentiation is a fundamental biological process whose dysregulation leads to disease. Single-cell sequencing offers unique insight into the differentiation process, but data analysis remains a major modeling challenge—particularly in complex branching systems e.g. hematopoiesis (blood cell development). Here, we extend optimal transport theory to address a previously inaccessible modeling problem: inferring developmental progression of differentiating cells from a single snapshot of an in vivo process. We achieve this by deriving a multistage transport model. Our approach accurately reconstructs cell fate decision in hematopoiesis. Moreover, it infers rare bipotent cell states and uniquely detects individual outlier cells that diverge from the main differentiation paths. We thus introduce a powerful mathematical framework that enables more granular analyses of cell differentiation.

Cell differentiation is a fundamental biological process. For example, the continuous differentiation of hematopoietic stem and progenitor cells in bone marrow ensures the replenishment of the blood cells in the adult setting, and dysregulation of the same process causes disease. The revolution in single-cell RNA-sequencing now enables the capture of a high-resolution snapshot of the cell differentiation process. The resulting data comprise the single-cell molecular profiles from a spectrum of developmental stages. Thus, a single snapshot has the potential to serve as a basis for comprehensively mapping the cell differentiation process at the molecular level. Cells from early and late stages of a given process can typically be identified; however, the mapping of the intermediate cells is hampered by a snapshot’s inherent lack of temporal labels that relate a cell’s state to a specific stage of differentiation. Computational approaches are therefore necessary to accurately map the cell differentiation process and infer the cells’ trajectories based on their gene expression measurements.

Optimal transport has recently been highlighted as a unifying mathematical framework to address many challenges related to the analysis of single-cell omics data, including trajectory inference (1). Current optimal transport-based trajectory inference methods perform well on time-series data (2–8), but their reliance on temporal information prevents application in the snapshot setting where the temporal identity of each cell is unknown—the common setting when studying primary cells in their in vivo context. The noteworthy exception is StationaryOT (9), which represents an elegant approach to applying classical optimal transport to a single snapshot. However, StationaryOT is fundamentally constrained by its bimarginal formulation: Traditional optimal transport operates over a single transport step between two known distributions, inherently restricting its capacity to capture temporal progression and hindering its broad utility in snapshot data. Extensions to optimal transport involving multiple marginals can address temporal progressions (10). However, in the single-cell snapshot setting, the lack of temporal labeling means that cells cannot be assigned to a specific marginal, further complicating the analysis.

Here, we develop MultistageOT (https://github.com/dahlinlab/MultistageOT). MultistageOT uses global information across all cells and differentiation stages to infer continuous trajectories between predefined initial and terminal states. While classical optimal transport requires two fixed marginal distributions, MultistageOT treats the intermediate unobserved marginal distributions as latent variables to be learned, allowing MultistageOT to infer pseudotime-resolved high-resolution trajectories from a single snapshot. In doing so, we extend the applicability of optimal transport to a previously inaccessible problem: establishing developmental progression within a single snapshot of in vivo cell differentiation. Notably, by learning the intermediate stages of development, MultistageOT is uniquely able to infer individual outlier cells unrelated to the analyzed differentiation process.

We benchmark MultistageOT using a well-established dataset with lineage tracing information. We demonstrate how our approach introduces a pseudotemporal dimension and that it outperforms StationaryOT in predicting the fates of the differentiating cells in a large-scale dataset of hematopoiesis. Finally, we show how MultistageOT identifies cell states that do not belong to the main cell differentiation process.

## Results

Here, we develop MultistageOT for the inference of cell differentiation trajectories in a snapshot of single-cell data. We give a brief explanation of the MultistageOT framework, test it using synthetic data, validate it using results from lineage tracing experiments of in vitro hematopoiesis, and apply it to multiple in vivo snapshots of hematopoiesis. Finally, we show that the framework generalizes beyond single-cell RNA-sequencing data, and to systems beyond hematopoiesis. For the mathematically interested readers, we rigorously develop the entire mathematical framework behind MultistageOT in *SI Appendix*, *Supplementary Note*.

### Trajectory Inference with MultistageOT.

Single-cell RNA-sequencing measures the gene expression profiles of individual cells. In a snapshot of single-cell data, we view the measured gene expression profiles as representing possible cell states during a differentiation process. Let the squared Euclidean distance between any two cell states correspond to the cost of transitioning between them. Note that this cost corresponds to the negative log-likelihood of transitioning under a Gaussian probability kernel. By minimizing a total transition cost, discrete bimarginal optimal mass transport can be used to match single cells sampled at two different stages of a differentiation process (Box 1). This approach has been used as a tool to infer one-step cell transitions from single snapshot data (9). However, cell differentiation involves multiple transitions over multiple differentiation stages, which corresponds to an unobserved pseudotemporal progression within the snapshot. Such a progression would naturally be captured by a dynamical formulation of optimal transport, but this has yet to be considered. To address this, we developed MultistageOT. MultistageOT extends bimarginal optimal transport (Box 1) to transport across multiple (more than two) marginals (Box 2), allowing cell differentiation in a snapshot to be modeled as a stepwise transportation process over multiple differentiation stages. Therefore, MultistageOT can be viewed as a dynamic formulation of optimal transport (see *SI Appendix*, *Supplementary Note*, for a more detailed discussion). In brief, MultistageOT assumes predefined initial and terminal distributions—constraining the solution space with prior biological knowledge—and infers the latent intermediate distributions by solving a convex optimization problem. Technically, this is achieved by simultaneously optimizing the unknown intermediate marginals under structural constraints (Box 2). This stitches together the intermediate differentiation stages and allows us to reconstruct a pseudotemporal order directly from the snapshot, without the need for time-series measurements. Since existing optimal transport solvers cannot accommodate latent intermediate distributions, we derived and implemented a generalized Sinkhorn algorithm, based on entropy-regularization, that accommodates the unknown intermediate cell differentiation stages (*SI Appendix*, *Supplementary Note*, *Algorithm 1*). Our implementation, together with a simple tutorial and user-friendly notebook scripts, is available at (https://github.com/dahlinlab/MultistageOT).

**Box 1: Optimal transport**

In the classical discrete optimal transport setting, two discrete mass distributions are given:

$$\sum_{i=1}^{n_{1}}\mu_{1}^{\left(i\right)}\delta_{x_{1}^{\left(i\right)}},\;\sum_{j=1}^{n_{2}}\mu_{2}^{\left(j\right)}\delta_{x_{2}^{\left(j\right)}},$$

where the ordered sets $X_{1}={\left(x_{1}^{\left(i\right)}\right)}_{i=1}^{n_{1}}$ and $X_{2}={\left(x_{2}^{\left(j\right)}\right)}_{j=1}^{n_{2}}$ correspond to the points of support of the respective distributions, and where $\mu_{1}^{\left(i\right)}$ and $\mu_{2}^{\left(j\right)}$ represent the mass in the points $x_{1}^{\left(i\right)}$ and $x_{2}^{\left(j\right)},$ respectively.

A transport plan is a matrix, $M={\left[m_{\mathrm{ij}}\right]}_{i=1,j=1}^{n_{1},n_{2}}\in R_{+}^{n_{1}\times n_{2}}$, whose component $m_{\mathrm{ij}}$ denotes the amount of mass transported from $x_{1}^{\left(i\right)}\in X_{1}$ to $x_{2}^{\left(j\right)}\in X_{2}$. We say that the transport plan is feasible if the total amounts transported are consistent with the initial and final distributions, i.e., if $M1_{n_{2}}=\mu_{1}$ and $M^{T}1_{n_{1}}=\mu_{2}$, where $\mu_{1}={\left[\mu_{1}^{\left(i\right)}\right]}_{i=1}^{n_{1}}$ and $\mu_{2}={\left[\mu_{2}^{\left(j\right)}\right]}_{j=1}^{n_{2}}$ are the vectors representing the mass distributions. Let

[1] $$\begin{array}{c} c_{\mathrm{ij}}:=\left|\right|x_{1}^{\left(i\right)}-x_{2}^{\left(j\right)}{\left|\right|}_{2}^{2} \end{array}$$

denote the cost of moving a unit of mass from $x_{1}^{\left(i\right)}$ to $x_{2}^{\left(j\right)}$, and let $C={\left[c_{\mathrm{ij}}\right]}_{i=1,j=1}^{n_{1},n_{2}}$ be the corresponding cost matrix. The optimal transport problem is to find a feasible transport plan that solves

[2a] $$\begin{array}{c} \begin{array}{c} \begin{array}{cc} T(\mu_{1},\mu_{2})\;:=\underset{M\in {\mathbb{R}}_{+}^{n_{1}\times n_{2}}}{\text{minimize}} & \langle C,M\rangle \end{array} \end{array} \end{array}$$

[2b] $$\begin{array}{c} \begin{array}{c} \begin{array}{cc} \text{subject to} & M1_{n_{2}}\;=\mu_{1} \end{array} \end{array} \end{array}$$

**Fig. 1.**
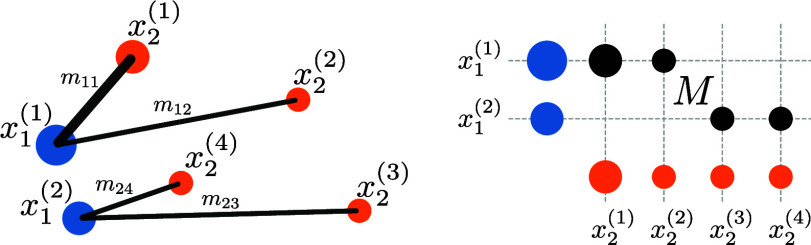
Optimal transport finds an optimal assignment between points in two point cloud distributions $\mu_{1}\in R_{+}^{2}$ (blue dots) and $\mu_{2}\in R_{+}^{4}$ (orange dots). The two distributions $\mu_{1}$ and $\mu_{2}$ could for example represent two cell populations. The solution is a matrix, $M\in R_{+}^{2\times 4}$, encoding the optimal way to redistribute the mass in $\mu_{1}$ to form $\mu_{2}$. The thickness of the black lines in the *Left* plot represents the amount of mass sent between the points in the optimal solution under the squared Euclidean cost [**1**]. The corresponding transport plan’s matrix elements in the *Right* plot can be interpreted as the strength of the association between any pair of points $x_{1}^{\left(i\right)}\in X_{1},x_{2}^{\left(j\right)}\in X_{2}$.

[2c] $$\begin{array}{c} \begin{array}{c} \begin{array}{cc} & M^{T}1_{n_{1}}=\mu_{2}, \end{array} \end{array} \end{array}$$

where ⟨·,·⟩ is the Frobenius inner product. An example of such a problem, together with a corresponding optimal solution, is given in Fig. 1. Note that when $\mu_{1}$ and $\mu_{2}$ are probability measures, then $T(\mu_{1},\mu_{2})$ defines the Wasserstein-2 metric between $\mu_{1}$ and $\mu_{2}$. In practice, it is common to solve an entropy-regularized version of the problem using Sinkhorn algorithms (11), corresponding to coordinate ascent on the dual function (12).

**Box 2: The multistage optimal transport problem**

**Fig. 2.**
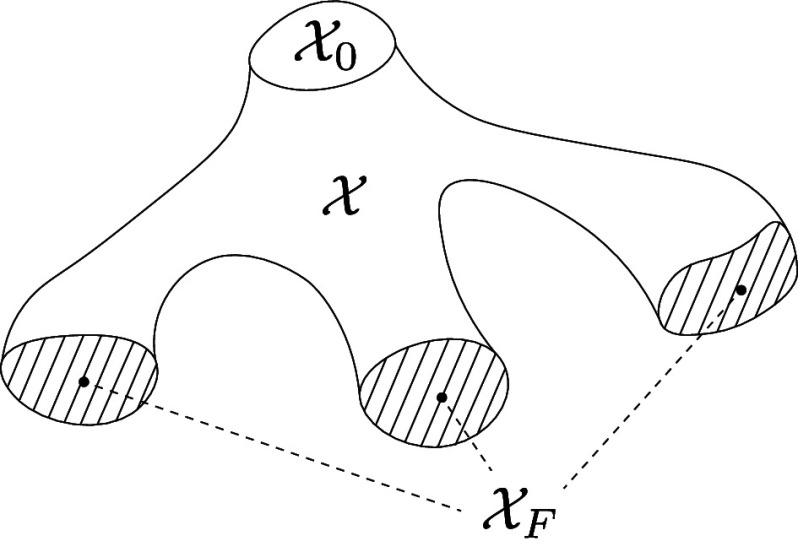
Conceptual figure of a scRNA-seq dataset partitioned into three subsets: $X_{0}$ (initial states), X (intermediate states), and $X_{F}$ (terminal states).

We assume a single-cell RNA sequencing snapshot of a developmental process is given, with corresponding sets of initial states (e.g., stem cells), denoted $X_{0}$, and terminal states (e.g., lineage committed cell types), denoted $X_{F}$. We refer to all other cells as intermediate states, denoted X (Fig. 2). Let $n_{0}:=\left|X_{0}\right|$, n:=|X| and $n_{F}:=\left|X_{F}\right|$ denote the cardinalities of each set.

**Fig. 3.**
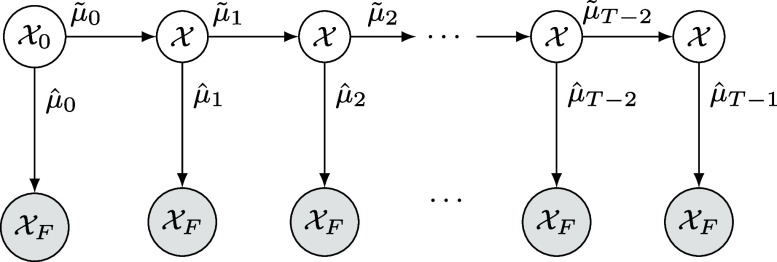
Graph representation of our general multistage optimal transport framework to model cell differentiation. In each time step t=0,1,⋯,T−1 mass can be sent either to intermediate states in X or to terminal states in $X_{F}$. Mass sent to X stays within the system, whereas any mass sent to $X_{F}$ permanently leaves the system. The intermediate distributions in t=1,⋯,T−1 are unknown, latent, variables learned by our method.

We model the transitions of cells from $X_{0}$ to $X_{F}$ as a multistage mass transport process, represented below in terms of latent marginals ${\tilde{\mu }}_{t}$ (mass that continues in the system) and ${\hat{\mu }}_{t}$ (mass that exits the system) in the *t*:th stage of transport (Fig. 3).

We compute the latent intermediate marginals and optimal transitions between the cells by solving the following multistage optimal transport problem (MultistageOT):

$$\begin{array}{c} \begin{array}{cc} \underset{\underset{t=0,\cdots ,T-1}{{\tilde{\mu }}_{t},{\hat{\mu }}_{t},{\hat{\nu }}_{t}}}{\text{minimize}}\; & \sum_{t=0}^{T-2}{\tilde{T}}_{t}\left({\tilde{\mu }}_{t},{\tilde{\mu }}_{t+1}+{\hat{\mu }}_{t+1}\right)+\sum_{t=0}^{T-1}{\hat{T}}_{t}\left({\hat{\mu }}_{t},{\hat{\nu }}_{t}\right)\\ \text{subject to}\; & {\tilde{\mu }}_{0}+{\hat{\mu }}_{0}\geq 1_{n_{0}}\\ & \sum_{t=1}^{T-2}{\tilde{\mu }}_{t}+\sum_{t=1}^{T-1}{\hat{\mu }}_{t}\geq 1_{n}\\ & \sum_{t=0}^{T-1}{\hat{\nu }}_{t}\geq 1_{n_{F}}, \end{array} \end{array}$$

where each term in the objective, i.e., ${\tilde{T}}_{t}\left(\cdot ,\cdot \right)$ or ${\hat{T}}_{t}\left(\cdot ,\cdot \right)$, is the optimal transport cost in a bimarginal optimal transport problem using the squared Euclidean distances between the cell’s gene expression states as ground metric (Box 1), and ${\hat{\nu }}_{t}={\left[{\left({\hat{\nu }}_{t}\right)}_{i}\right]}_{i=1,\cdots ,n_{F}}$ is the mass received by cells in $X_{F}$ in stage *t*. In practice, we solve an entropy-regularized version of MultistageOT nested in a proximal point scheme (see *SI Appendix*, *Supplementary Note* for full details and derivation).

Conceptually, MultistageOT leverages mass transport to infer the optimal transitions between cell states. The transport of mass in each differentiation stage can be interpreted in terms of transition likelihoods: The more mass a cell sends in a particular differentiation stage, the more likely it is to transition in that stage. This induces a probability distribution that models how probable a cell is to transition to a nearby cell state in each differentiation stage (*SI Appendix*, *Supplementary Methods*). MultistageOT thus provides a natural basis for pseudotemporal ordering of the cells and for inferring cell fate potential (*SI Appendix*, *Supplementary Methods*).

### MultistageOT Establishes Differentiation Trajectories in Unstructured Snapshot Data.

**Fig. 4.**
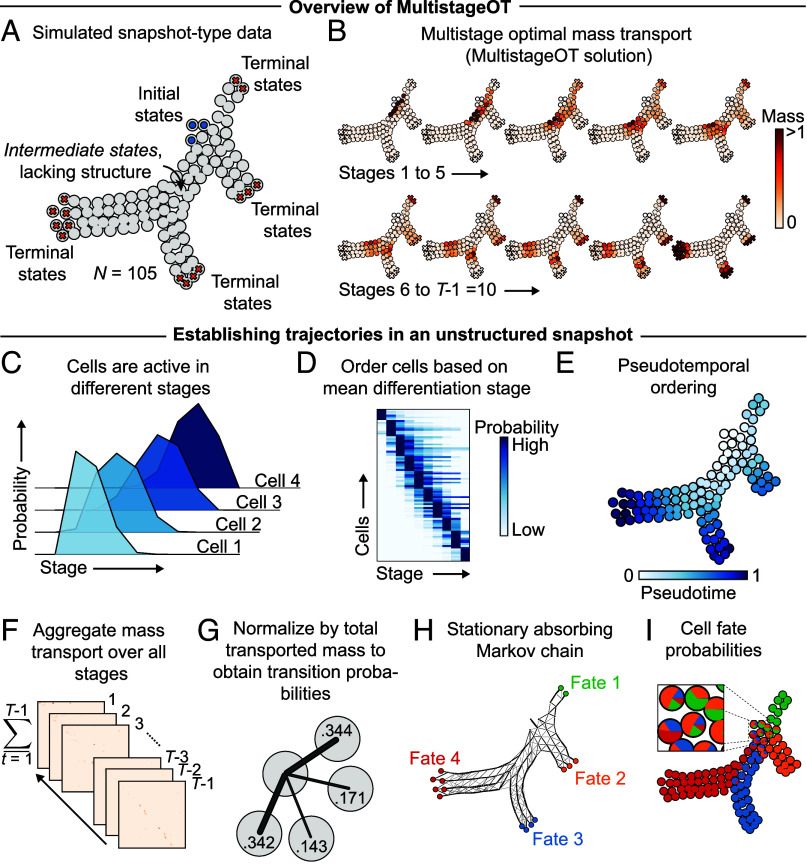
MultistageOT orders simulated snapshot data along a pseudotemporal axis and induces state transition probabilities between data points (*SI Appendix*, *Supplementary Methods*). (*A*) Two-dimensional data (N=105) was generated in the unit square to mimic samples from a developmental process with known initial and terminal states. (*B*) MultistageOT solves an entropy-regularized multistage optimal mass transport problem over *T* stages, modeling transitions between cell states in a discrete-time differentiation process, where each transport stage models a differentiation step (see *SI Appendix*, *Supplementary Note* for details). Here, T=11. The plot visualizes the optimal way for cells to transition from initial into terminal states, achieved by plotting the total transported mass out from each cell in each stage t=1,⋯,10. (*C*) Mass transport varies across the differentiation stages, inducing a probability distribution for each cell over the differentiation stages. The plot shows the probability profiles of four example cells. (*D*) The varying distributions of cells across the differentiation stages establishes a pseudotemporal order, from early- to late-stage transporters. (*E*) Cells ordered along a pseudotemporal axis based on mean transport stage. (*F*) The transport maps are aggregated over all stages to obtain the total likelihood of transition between any cell pair. (*G*) The aggregated likelihood matrix is normalized such that each matrix element represents a transition probability between a cell pair. (*H*) The MultistageOT-based transition probabilities define a stationary absorbing Markov chain in which the terminal states represent different classes of absorbing states. (*I*) Under the MultistageOT-induced Markov chain, each cell has certain probability of being absorbed in each fate. We refer to these as cell fate probabilities, and represent them as a pie chart for each cell.

As a proof-of-concept, MultistageOT was first applied on synthetic data. A set of data points was generated in the 2D unit square, representing cell states from a differentiation process with known initial and terminal states (Fig. 4*A*). We employed MultistageOT for finding the optimal transitions from the initial states to the terminal states (using *SI Appendix*, Algorithm 1; see *SI Appendix*, Supplementary Note)—hypothesizing that MultistageOT would find a pseudotemporal progression through the states consistent with the shape of the data.

Visualizing the mass transport in the optimal solution revealed a gradual progression through the intermediate cell states (Fig. 4*B*). As expected, cells near the initial states transitioned (transported mass) in earlier differentiation stages compared to cells near the terminal states (Fig. 4*B*). The varied distribution over the differentiation stages (Fig. 4 *C* and *D*) enables a ranking of the cell states based on their mean differentiation stage: Each cell is assigned a normalized pseudotime index between 0 and 1, with 0 corresponding to the earliest mean differentiation stage and 1 to the latest mean differentiation stage (*SI Appendix*, *Supplementary Methods*). In the 2D dataset, the pseudotime values produced a smooth heatmap gradient from the initial to the terminal states (Fig. 4*E*), consistent with the shape of the data.

To infer cell fates, we assume a Markov chain model. First, the general affinity between any two cells is quantified by summing, over all differentiation stages, the mass transported between the cells (Fig. 4*F*). The normalized aggregated mass transport between each cell pair is then interpreted as a transition probability in a stationary absorbing Markov chain (Fig. 4*G*). To infer likely cell fates, we let different groups of terminal states define different classes of absorbing states in the Markov chain. Each class of absorbing states represents a fate (Fig. 4*H*). For each cell, we compute the probability of it being absorbed in the different fates under the Markov chain model, and refer to this as cell fate probabilities (see *SI Appendix*, *Supplementary Methods* for additional details).

To assess the utility of the MultistageOT framework in predicting likely cell fates, we considered the four groups of terminal states in each of the diverging arms of the data (Fig. 4*A*) to represent cells committed to four different cell lineages. We computed cell fate probabilities and graphed each cell’s predicted fate probabilities as a pie chart on top of their state coordinates. This enabled joint visualization of how lineage commitment changed as a function of cellular state for each lineage (Fig. 4*I*). The estimated cell fate probabilities were consistent with the shape of the data; cells near the initial states reflected multipotency, whereas cell states located in the diverging arms of the data reflected full commitment to a particular fate (Fig. 4*I*). The MultistageOT algorithm (*SI Appendix*, Algorithm 1) depends on a regularization parameter controlling the level of diffusion in the optimal transitions. Shannon entropy quantifies the degree of multipotency reflected in the predicted cell fate probabilities. The degree to which cells exhibit multipotency, as measured by mean Shannon entropy over all cells, increased with increasing regularization parameter values (*SI Appendix*, Fig. S1), as expected.

**Fig. 5.**
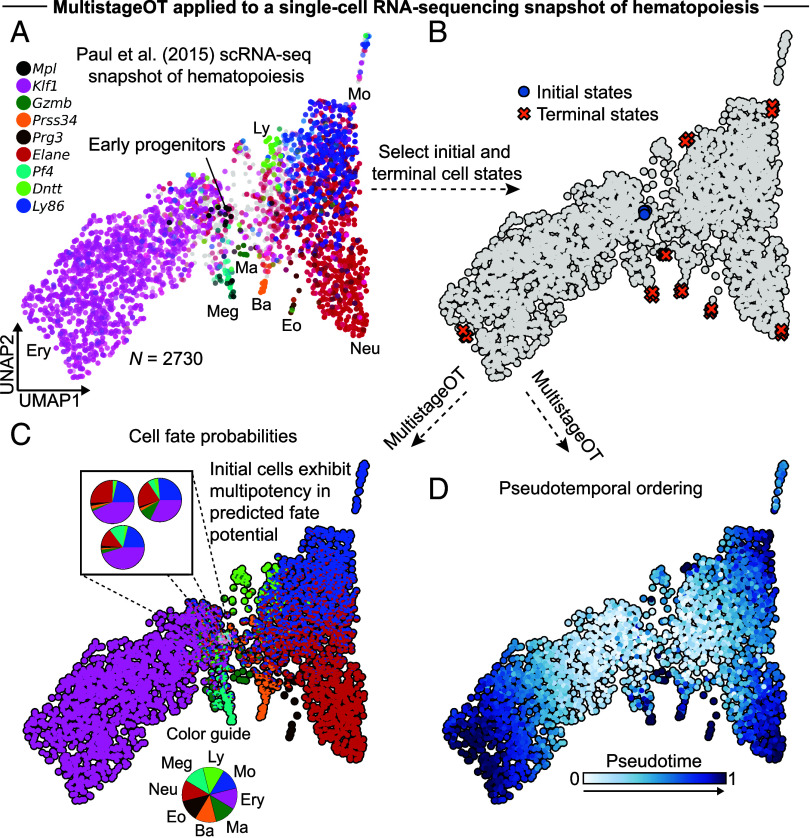
MultistageOT-based inference of fate potential and pseudotemporal ordering of single cells in a publicly available single-cell RNA-sequencing snapshot of hematopoiesis. (*A*) Layered gene expression level (log(*x*+1)-transformed) plot of known marker genes for early progenitors and eight mature blood cell lineages graphed on a UMAP embedding of the data from Paul et al. (13). Abbreviations: Meg (megakaryocyte), Ery (erythroid), Ma (mast cell), Ba (basophil), Eo (eosinophil), Neu (neutrophil), Mo (monocyte), and Ly (lymphoid). (*B*) Marker genes were used to orient the UMAP and choose initial and terminal cell states as input to our MultistageOT model. (*C*) MultistageOT-based inference of fate potential. Each cell’s UMAP coordinates are graphed as a pie chart, representing the MultistageOT-based fate probabilities (*D*) Pseudotemporal ordering of the cells based on the optimal mass transport.

The results based on synthetic data encouraged the testing of the MultistageOT framework on a single-cell RNA-sequencing snapshot of a cell differentiation process. The complex process of hematopoiesis was chosen to challenge the framework (Fig. 5*A*). We applied MultistageOT to the dataset of Paul et al. (13), selecting multipotent progenitors as initial states and progenitors at the entry points to each of 8 distinct cell lineages as terminal states (Fig. 5 *A* and *B*). UMAP visualization of the MultistageOT-based results revealed progressively increasing pseudotime values and lineage commitment from the multipotent progenitor region to the cell lineage entry points (Fig. 5 *C* and *D*), results consistent with how hematopoietic stem and progenitor cells differentiate into the various cell types. To evaluate the robustness of MultistageOT’s downstream results with respect to different choices of hyperparameters, including the choice of initial and terminal cells, the number of transport stages and the regularization strength, we performed a sensitivity analysis (*SI Appendix*, Fig. S2). We found that the cell fate probabilities and pseudotime estimates were overall robust with respect to changes to the values of these hyperparameters (*SI Appendix*, Fig. S2 *A*–*P*).

Taken together, MultistageOT infers trajectories in unstructured snapshot data using multistage optimal mass transport. The mass transport induces state transition probabilities between data points, representing an affinity between cell states, as well as a pseudotemporal axis, representing the degree of differentiation.

### Cell Fate Predictions Based on MultistageOT Matches Clonal Sister Fates Observed In Vitro in Mouse Hematopoiesis.

Thus far, we have shown that MultistageOT can produce reasonable downstream results in a single-cell snapshot. However, the dataset of Paul et al. (13) (Fig. 5) does not include a reference or ground truth to quantitatively assess MultistageOT’s performance in predicting cell differentiation. To evaluate the accuracy of MultistageOT, we utilized the dataset of Weinreb et al. (14), which provides longitudinal single-cell RNA-sequencing data of in vitro hematopoiesis, integrated into a unified snapshot-like landscape of cell states. Notably, the dataset includes lineage tracing information of cell clones, which can serve as proxy for the ground truth trajectories of individual cells.

**Fig. 6.**
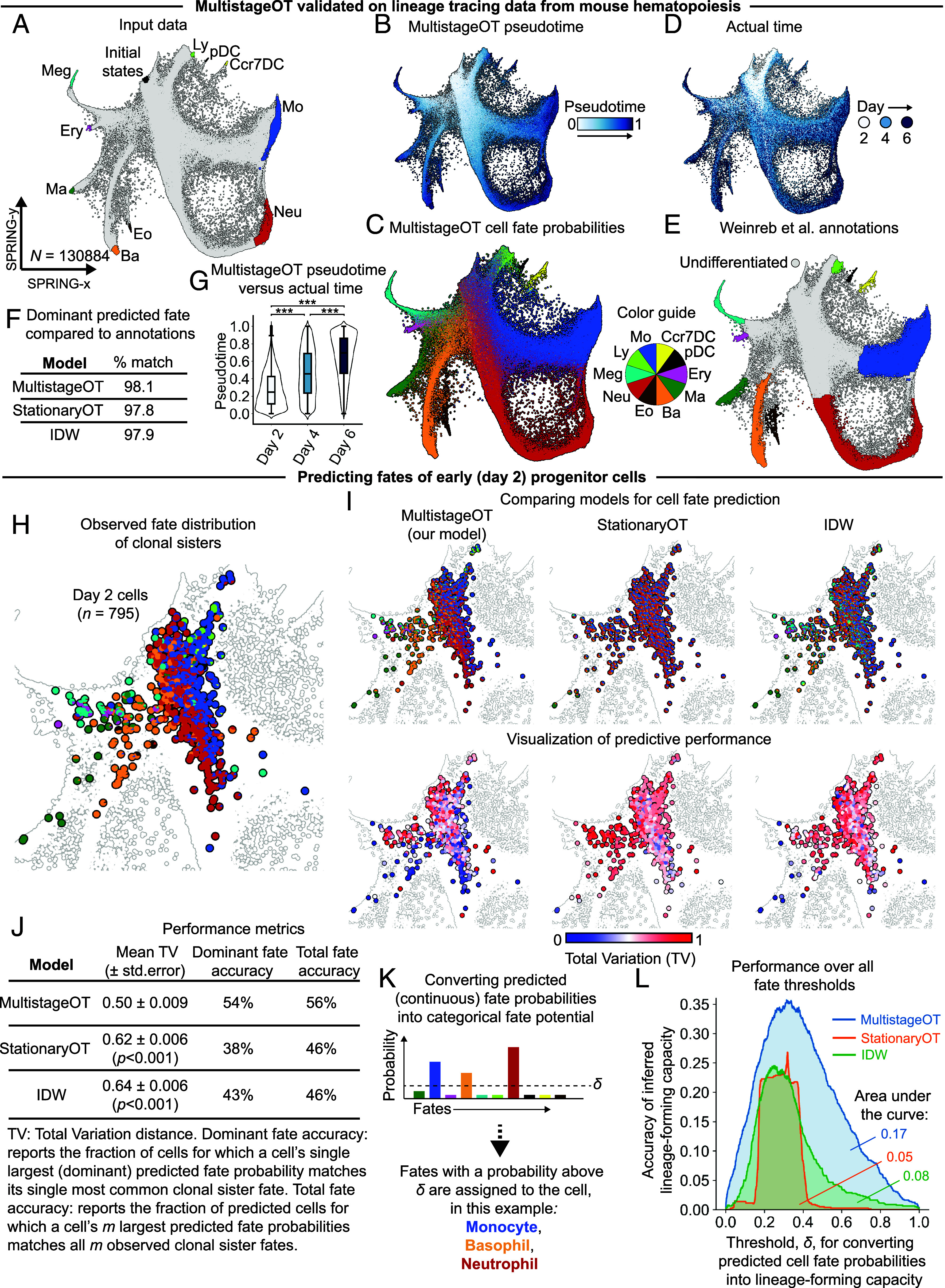
Validation of the MultistageOT framework with in vitro lineage tracing data from mouse hematopoiesis. (*A*) SPRING-plot of the data from Weinreb et al. (14) highlighted with initial and terminal cell states in different lineages. (*B*) MultistageOT-based pseudotemporal ordering. (*C*) MultistageOT-based inference of cell fate potential. Each cell is represented by a pie chart with wedge sizes proportional to the predicted probability of the cell ending up in each fate. (*D*) Experimental time point for each cell. (*E*) Cell type annotation given by Weinreb et al. (14). (*F*) Accuracy when comparing annotated cell label against dominant predicted fate potential. (*G*) Box plots of MultistageOT pseudotime. Each box extends from the first to the third quartile of the data. The black horizontal line indicates the median. Whiskers extend to the farthest pseudotime still within 1.5 times the interquartile range. Mann–Whitney *U* test (two-sided), ***P<0.001. (*H*) “Ground truth” fate distribution of clonal sisters for day 2 cells. (*I*) Benchmarking fate predictions of MultistageOT (*Top Left*), StationaryOT (*Top Middle*), and an Inverse Distance Weighted (IDW) model (*Top Right*). *Bottom* row shows corresponding errors (total variation distance, TV) in the predictions when compared to the distribution of clonal sister fates. (*J*) Performance metrics for each method. The *P*-values correspond to comparisons to MultistageOT in a Mann–Whitney *U* test (two-sided). (*K*) Assigning a cell to fates with predicted fate probability above a threshold, denoted *δ*. (*L*) Predictive performance for each of the three models over all possible thresholds for *δ* (horizontal axis). The vertical axis reports the fraction of cells whose model-based assignment of fate potential matches the observed clonal sister fates. Abbreviations: Meg (megakaryocyte), Ery (erythroid), Ma (mast cell), Ba (basophil), Eo (eosinophil), Neu (neutrophil), Mo (monocyte) pDC (plasmacytoid dendritic cell), Ccr7DC (Ccr7^+^ DC), and Ly (lymphoid).

The Weinreb et al. (14) dataset consists of 130,887 cells. To satisfy the memory requirements of a standard laptop computer, we uniformly subsampled cells into 12 disjoint subsets of size N= 10,907 cells and MultistageOT was applied to each subset. Initial and terminal cell states were selected (Fig. 6*A*, and *Materials and Methods*), corresponding to multipotent progenitors and the most differentiated progenitors in the landscapes, respectively. Pooling the MultistageOT results from each subset generated a unified cell landscape, in which the cells were pseudotemporally ordered and the fate potential of each cell was predicted (Fig. 6 *B* and *C*).

Visualization of the MultistageOT-derived pseudotime values showed progressively increasing pseudotime from undifferentiated progenitors to the mature cells (Fig. 6 *B* and *E*). Overall, these MultistageOT-derived pseudotime values were consistent with actual time, showing a significant tendency to increase with the cells’ experimental time stamps (Fig. 6 *D* and *G*).

Visualization of the MultistageOT-based inference of fate potential of the cells displayed a gradual increase in lineage commitment as the cells approached the terminal states (Fig. 6*C*), which is expected from differentiating cells. StationaryOT (9) was applied to the same dataset to allow a side-by-side comparison with the state-of-the-art optimal-transport-based trajectory inference method (*SI Appendix*, Fig. S3*A*). As reference, we also developed a naive predictor of cell fate referred to as Inverse Distance Weighted (IDW). The IDW method is not based on optimal transport and instead uses the relative Euclidean distances to the mature cell types to infer the cell fate potential (*SI Appendix*, Fig. S3 *B* and *C* and *Supplementary Methods*).

The predictions made with MultistageOT, StationaryOT, and IDW reflected increasing commitment as cells approach the terminal cells (Fig. 6*C* and *SI Appendix*, Fig. S3 *A* and *C*). Each model scored similarly in the simple task of predicting fates of cells that Weinreb et al. (14) had annotated as mature (Fig. 6*E*). Specifically, MultistageOT recovered the dominant cell fate in 98.1% of the cells. StationaryOT and IDW reached 97.8% and 97.9% matches respectively (Fig. 6*F*).

To rigorously benchmark each method, we used the lineage tracing data of Weinreb et al. (14) as proxy for the ground truth fate potential and evaluated their performance in predicting the fates of the most immature progenitors of the dataset. We specifically analyzed the progenitors belonging to the earliest time point (day 2) that had clonal sister cells in any of the subsequent time points (days 4 or 6) (*SI Appendix*, Fig. S4 and *Supplementary Methods*). Visual comparison between the clonal fates and the three models’ predictions—using colors to represent the unique cell fates—revealed striking similarities in the color patterns of MultistageOT compared to the observed fate distribution, with seemingly complete matches in some cells (Fig. 6 *H* and *I*), whereas StationaryOT showed a tendency to predict substantial multipotency across all day 2 progenitors, with less obvious color matching compared with the observed fates. The color pattern of IDW appeared as a hybrid between the MultistageOT and StationaryOT results (Fig. 6*I*).

We evaluated several metrics to quantify the degree of similarity between the observed fates and the model predictions (Fig. 6*J*). The output of MultistageOT, StationaryOT, and the IDW predictor are probability distributions that for each cell predict the likelihood of each fate. We therefore assessed Total Variation (TV) distance (Fig. 6*I*, *Bottom*), which measures the total absolute deviations between two probability distributions. In doing so, we took each cell’s clonal sister fate distribution in days 4 or 6 as the reference, “ground truth,” distribution (*SI Appendix*, *Supplementary Methods*). The mean TV was found to be significantly lower in MultistageOT compared to StationaryOT and IDW (Fig. 6*J*), indicating that MultistageOT’s fate predictions best matched the observed clonal sister fate distributions. We then turned to viewing fate prediction in terms of a classification problem and benchmarked the accuracy of a model’s dominant fate prediction compared to the single most common fate among the cell’s clonal sisters in days 4 or 6. We call this the dominant fate accuracy. Notably, MultistageOT achieved higher dominant fate accuracy than StationaryOT and IDW (Fig. 6*J*).

We next evaluated a metric that accounts for the observation that some progenitors give rise to multiple cell lineages. This metric, which we termed total fate accuracy, quantifies how often a cell’s observed clonal sister fates correspond to a model’s dominant fate predictions. For example, if a cell’s clonal sisters in days 4 or 6 are observed to be neutrophils and monocytes, then the model’s prediction for that cell is considered a correct match only if the neutrophil and monocyte fates correspond to the model’s two highest predicted fate probabilities. MultistageOT achieved the highest total fate accuracy score compared with StationaryOT and IDW (Fig. 6*J*).

As a final evaluation, we assigned lineage-forming capacity if the model-inferred fate probability for a lineage was higher than a threshold *δ*, which converted the progenitors’ model-inferred fate probabilities into categorical data (Fig. 6*K*). We then compared the inferred lineage-forming capacity to the observed clonal fates of each progenitor for different values of *δ*. An assignment was considered correct only if the model-inferred lineage-forming capacity and observations matched completely. Notably, MultistageOT performed better than StationaryOT and IDW across all values of *δ* (Fig. 6*L*).

For additional reference, we compared MultistageOT against two recently published methods not based on optimal transport (*SI Appendix*, Fig. S5): CellRank2 (15) and StaVIA (16). CellRank2 provides two methods for single snapshot cell fate prediction: “PseudotimeKernel” and “ConnectivityKernel.” The connectivity kernel predicted substantial multipotency across a large portion of the cell landscape (*SI Appendix*, Fig. S5 *A*, *Left*), and showed low accuracy in predicting fates of day 2 cells (*SI Appendix*, Fig. S5 *C* and *D*). The pseudotime kernel showed competitive predictive performance (*SI Appendix*, Fig. S5 *A*, *Right*) but was less accurate than MultistageOT in predicting the fates of the day 2 cells (*SI Appendix*, Fig. S5 *C* and *D*). StaVIA’s fate predictions, reflecting substantial multipotency with strong lymphoid bias, corresponded to the lowest accuracy among the benchmarked methods (Fig. 6 *J* and *L*, and *SI Appendix*,Fig. S5 *B*–*D*).

Taken together, MultistageOT predicts the fate of differentiating progenitors in an integrated snapshot-like dataset more accurately than StationaryOT, IDW, CellRank2, and StaVIA.

### MultistageOT Identifies Outliers and Predicts Bipotent Progenitors in a Snapshot of Bone Marrow Hematopoiesis.

Modeling cell differentiation in an in vivo snapshot, i.e., a single time point measurement of the primary progenitors’ transcriptome, can be associated with challenges. For example, technical limitations in cell isolation can result in the unintentional capture of cells not belonging to the differentiation process, leading to a dataset containing outliers. In turn, this risks the inadvertent inference of biologically implausible trajectories. To explore the in vivo snapshot setting further, we applied MultistageOT to a publicly available single-cell RNA-sequencing dataset of hematopoiesis. This dataset comprises the gene expression profiles of more than 44,000 primary hematopoietic stem and progenitor cells isolated from mouse bone marrow (17). Notably, the dataset includes hematopoietic stem cells and 8 author-annotated entry points to distinct hematopoietic cell lineages, which were used to define the initial and terminal states in MultistageOT (*SI Appendix*, Fig. S6 and *Materials and Methods*). In computing the transition costs between cells, we identified a small number of cells at extreme distances from other cells (*SI Appendix*, Fig. S7*C*). Large transition costs cause numerical instability if the distances are too extreme, causing the computer to interpret every possible transition as infeasible. Such instability issues may be combated by increasing the level of regularization in the optimal transport maps. However, this may lead to undesirable levels of diffusion in the optimal transitions if the parameter necessary for stability grows large enough (*SI Appendix*, Fig. S1). We hypothesized the presence of outlier cells, not representing intermediate states between the selected initial and the terminal states, as the cause of these large transition costs.

**Fig. 7.**
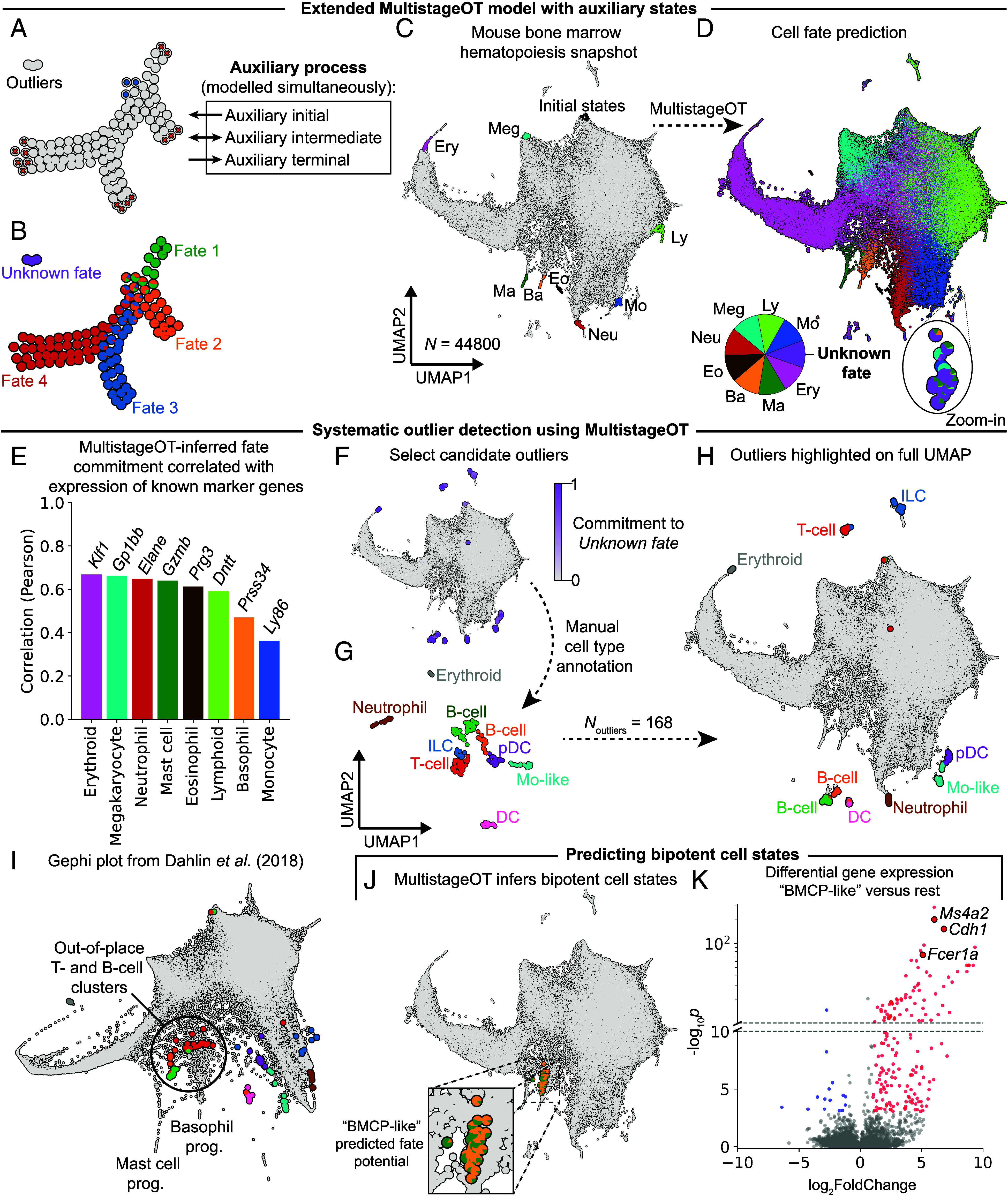
MultistageOT identifies outliers and predicts bipotent progenitors in a snapshot of bone marrow hematopoiesis. (*A*) The 2D synthetic dataset with the addition of a small population of outliers; auxiliary states enable MultistageOT to model the development of outliers separately. (*B*) MultistageOT-based cell fate probabilities, represented by pie charts. The “unknown fate” (purple) represents absorption in the auxiliary terminal state. (*C*) UMAP of Dahlin et al. (17) single-cell RNA sequencing data from mouse hematopoiesis, highlighted with initial and terminal cell states in different lineages. (*D*) MultistageOT-based cell fate probabilities. (*E*) log(x+1)-expression of each lineage associated gene marker (graphed in *SI Appendix*, Fig. S6) correlated (Pearson) against MultistageOT’s predicted commitment to the associated lineage. (*F*) Predicted commitment to an “unknown fate” was used as a means to identify outliers. Cells committed to “unknown fate” with a probability above 0.5 are highlighted. (*G*) $N_{\text{outlier}}=168$ candidate outlier cells with “unknown fate” probability above 0.5 were selected. The candidate outliers were clustered, represented in a new UMAP embedding and annotated. (*H*) Annotated outlier cells highlighted on the original UMAP. (*I*) Annotated outlier cells highlighted on the gephi embedding used by Dahlin et al. (*J*) MultistageOT predicts mast cell/basophil common progenitors (above 10% in mast cell and basophil commitment, and below 1% in all other fates). (*K*) Volcano plot showing log_2_-fold change of gene expression levels against the Benjamini–Hochberg adjusted *P*-value in a Mann–Whitney *U* test (two-sided) of log(*x*+1)-transformed gene expression in the BMCP-like subset versus the rest of the cells in the snapshot. Positive fold change indicates higher expression in the BMCP-like subset. Genes with absolute fold change above 1.0 and adjusted *P*-value below 0.001 are highlighted (blue and red indicates negative and positive fold change respectively). Note the broken *y*-axis. Abbreviations: Meg (megakaryocyte), Ery (erythroid), Ma (mast cell), Ba (basophil), Eo (eosinophil), Neu (neutrophil), Mo (monocyte), Ly (lymphoid), DC (dendritic cell), pDC (plasmacytoid DC), Mo-like (monocyte-like), ILC (innate lymphoid cell), and prog. (progenitor).

To be able to identify and account for potential outliers, we developed an extension of the MultistageOT model. The extension works by introducing three auxiliary states (corresponding to an initial, intermediate, and terminal cell state) to which cells will transition if the main transportation process is associated with too high cost. This allows outliers to be modeled in a separate auxiliary process (Fig. 7*A*), and stabilizes the algorithm with respect to extreme transition costs, without necessitating undesirably large levels of regularization. To test this extension, we added outliers to the simulated data in Fig. 4*A*. To mark cells as outliers, we let the auxiliary terminal state represent an “unknown fate,” expecting MultistageOT to highlight outliers with strong commitment to this “unknown fate” (*SI Appendix*, Fig. S8). Notably, the extension of MultistageOT was able to identify the outlier cells in the simulated data (Fig. 7 *A* and *B*).

The in vivo hematopoiesis dataset (17) (Fig. 7*C*) was subsampled into four disjoint subsets of size N=11,200 cells and MultistageOT was applied to each subset. Pooling the results and plotting the fate probabilities for each cell highlighted the progression from multipotent progenitors to lineage-committed cells and revealed potential outlier cells that were marked by MultistageOT as committed to the “unknown fate” (Fig. 7*D*). The hematopoietic progenitors’ ground truth fate potential is unavailable in a single snapshot of hematopoiesis. To assess the validity of the inference, we therefore correlated the MultistageOT-derived fate potential with established lineage marker genes. The results revealed positive correlation for each of the eight mature cell lineages and the corresponding marker gene (Fig. 7*E*).

A small number of cells were predicted to have 50% or higher probability to be absorbed in an unknown fate (Fig. 7*F*). We considered such cells candidate outliers. To inspect these cells further, we visualized them in a new UMAP embedding and performed Leiden clustering analysis (Fig. 7*G*). The candidate outlier clusters were annotated based on the expression levels of marker genes (*SI Appendix*, Figs. S9–S12). Two of the clusters expressed genes representative of cells in late stages of neutrophil and erythrocyte differentiation, respectively (*SI Appendix*, Fig. S9 *D* and *E*). As these neutrophil and erythroid cells are formed beyond the entry points to each respective lineage identified by Dahlin et al. (17), they do not represent intermediate differentiation stages in the snapshot and MultistageOT appropriately flagged them as potential outliers. The annotation also revealed the presence of cells with lineage programs representative B cells, T cells, and innate lymphoid cells (ILCs) (Fig. 7*G*). These lineages emerge past the lymphoid lineage entry point that was identified by Dahlin et al. (17), further evidence that the candidate cells constituted outliers in the analyzed differentiation process. We observed that the B-, T-, and ILC-like cells occupied distinct regions of the UMAP, separate from the main single-cell landscape and the lymphoid entry point. This served as an additional indicator that the identified cells did not belong to the differentiation process. However, we also note that MultistageOT is independent of data visualization approach and can therefore reveal outliers in situations where the data visualization fails to clearly distinguish them. The relevance of this becomes clear when the data is visualized with the original gephi embedding used by Dahlin et al. (17). In the gephi-plot, the B- and T-like cells are found out-of-place as they position near the entry point to the basophil and mast cell differentiation trajectories (Fig. 7*I*). Taken together, by detecting individual outlier cell states, MultistageOT offers a unique ability to prevent the inference of biologically implausible trajectories—enabling robust trajectory inference even at the single-cell level.

The identification of bipotent progenitor stages is crucial for the aim of resolving lineage diversification in the cell differentiation process. Dahlin et al. (17) experimentally demonstrated the existence of bipotent basophil-mast cell progenitors (BMCPs) in bone marrow through an educated guess of the progenitors’ phenotype. Here, we investigated whether MultistageOT’s data-driven inference of fate potential predicted the existence of bipotent BMCPs in this snapshot. Visualization of cells predicted by MultistageOT to possess specific basophil- and mast cell-forming capacity (*SI Appendix*, *Supplementary Methods*) revealed a population of BMCP-like cells located near the basophil and mast cell entry points on the UMAP (Fig. 7*J*). Differential gene expression analysis between the MultistageOT-inferred BMCP-like cells and all other cells (Fig. 7*K*) revealed high expression of the genes coding for the *α* and *β* chains of Fc*ϵ*RI (*Fcer1a*, Ms4a2) and E-cadherin (*Cdh1*), genes shown to mark progenitors with basophil- and mast cell-forming capacity (17, 18). Thus, MultistageOT achieves an unsupervised in silico recovery of BMCP-like intermediates, a rare bipotent progenitor population whose existence was experimentally demonstrated in the original publication of this dataset (17).

Taken together, MultistageOT infers cell fate potential consistent with known gene markers, detects outlier cell states, and predicts bipotent progenitors in a single-cell RNA-sequencing snapshot of cell differentiation.

### Generalizability to Different Systems and Modalities.

To explore its generalizability beyond mouse hematopoiesis, we applied MultistageOT to data from the cell atlas generated by Shahan et al. (19), featuring the RNA expression profiles of individual cells in the *Arabidopsis thaliana* root. *A. thaliana* exhibits a typical growth pattern (20), in which cells develop from a stem cell niche (SCN) located in the root tip, with the youngest cells adjacent to the SCN and older cells displaced further away from it (19). This spatiotemporal organization facilitates lineage tracing (19), making it a suitable system for testing trajectory inference methods. Applying MultistageOT inferred a developmental progression from the SCN to terminal states consistent with the cell type annotations (*SI Appendix*, Fig. S13 *A*–*D*). Notably, MultistageOT achieved 81% dominant fate accuracy in predicting the fates of the intermediate cell states (*SI Appendix*, Fig. S13 *B* and *C*), suggesting that its inferred trajectories were well aligned with true biological trajectories.

Thus far, we have applied MultistageOT to well-established single-cell RNA-sequencing data from two different developmental processes, showing that it generalizes well across kingdoms (Animalia and Plantae). We next explored the human setting in particular, applying MultistageOT to a recently published multiomic in vivo snapshot of human bone marrow hematopoiesis by Weng et al. (21). The data feature the joint RNA and chromatin accessibility profiles of differentiating human progenitor cells undergoing hematopoiesis (21). Applying MultistageOT on the joint profiles (*SI Appendix*, *Supplementary Methods*, and Fig. S13*E*) allowed us to establish a unified map of how fate commitment and pseudotime change across the human hematopoietic landscape (*SI Appendix*, and Fig. S13 *F* and *G*), demonstrating applicability of MultistageOT both in the human in vivo setting and with different modalities.

Together, the results demonstrate that MultistageOT is not restricted to a single development system and that the applicability of MultistageOT stretches across biological kingdoms and beyond the setting of single-cell RNA-sequencing data.

## Discussion

Data-driven computational methods that reliably infer hidden developmental trajectories from a single-cell RNA-sequencing snapshot are necessary for charting the cell differentiation process at the molecular level. In this work, we presented a multistage optimal transport model of cell differentiation trajectories.

Since cell differentiation underlies a progression from undifferentiated states to more differentiated states, a central property of computational models that seek to infer biological relationships between cell states in a snapshot is directional bias. Note that while the concept of RNA velocity (22) has received much attention for its potential use in inferring local directions of RNA states, the RNA velocity model does not infer relationships between the cells. To infer such relationships, MultistageOT follows established methods in trajectory inference (e.g., refs. 9 and 23–27) and takes the view of a single-cell snapshot as a graph, where nodes represent feasible differentiation states to which cells can transition, and edge weights represent affinities between states. However, naively building affinities between cells through a *k*-nearest neighbor graph based on Euclidean distances alone would fail to capture this directional bias as symmetries in neighbor proximity generally lead to undirected graphs. Directionality can be imprinted on a cell–cell affinity graph in different ways: by explicitly incorporating directional estimates in the affinity calculations, e.g. based on the RNA velocity model (22) as in ref. 27; or by applying post hoc modifications on the graph, pruning edges that do not conform with a secondary pseudotime model (26); or by leveraging prior knowledge of initial and terminal cell states (9). Building on this latter strategy, MultistageOT represents a dynamic multistage approach to computing the cell–cell affinities. MultistageOT infers global differentiation trajectories across a latent temporal progression of the intermediate states, allowing for pseudotemporal ordering as well as cell fate prediction. This is achieved via a dynamic optimal transport formulation over multiple transport stages, which enables a discrete-time approximation of the continuous-time differentiation process (see *SI Appendix*, *Supplementary Note* for the full derivation and a discussion about the connection between MultistageOT and continuous-time optimal transport). Finally, note that the concepts of RNA velocity and MultistageOT are not mutually exclusive. MultistageOT’s flexible structure in principle allows it to incorporate dynamics in its cost-function to regularize the optimal transport maps. The dynamics could for example be based on RNA velocity, which would be an interesting direction for future work.

We derived an algorithm for applying MultistageOT to a single-cell RNA-sequencing snapshot. The discretization of the differentiation process into multiple differentiation stages in MultistageOT enables a large degree of control and modeling flexibility, as constraints can be imposed on each differentiation stage. Notably, our MultistageOT implementation showed improved predictive performance over the state-of-the-art StationaryOT (9) algorithm when inferring fate potential of early hematopoietic progenitor cells. One limitation in our implementation of MultistageOT is the memory requirement, leading us to subsample the larger datasets to around $10^{4}$ cells. However, the subsampling approach had the advantage of allowing us to affirm that MultistageOT produced consistent annotations across multiple subsamples. For increased robustness, the data could in principle be repartitioned multiple times and estimates could be averaged over the resamplings for each cell (9). Note that the memory bottle neck remains an issue in standard optimal transport formulations as well, as the number of decision variables generally grows quadratically with the number of points in each support, whereas it grows linearly with the number of differentiation stages in MultistageOT for a fixed number of cells. However, recent work (5) has proposed leveraging online computation of the optimal transport costs to reduce memory cost, combined with GPU acceleration to combat the accompanying increase in compute time. Although demonstrated by Klein et al. (5) for a different setting than ours, we note that the benefits might extend to broader classes of optimal transport formulations, and we thus see potential in future implementations of MultistageOT to experiment with similar techniques.

A single-cell RNA-sequencing snapshot may feature disconnected clusters of cells, or even individual cells, that lie far away from other cells in gene expression space. In previous work, the issue of disconnectedness in a snapshot has been addressed by leveraging clustering algorithms and identifying disconnected clusters on the basis of a “connectivity measure” (25). However, methods have yet to be established that can automatically detect and highlight single cells that do not belong to the differentiation process of interest. Such outliers can create spurious trajectories through the data, which in turn may lead to incorrect conclusions about the underlying cell differentiation process. MultistageOT takes a fine-grained approach to address this issue: Auxiliary states act as a layer of stem, intermediate, and mature cells that bridges the disconnected cells to a hidden process, allowing for identification of outliers at single-cell resolution on the basis of inferred cell fate potential. Using our algorithm, MultistageOT was employed for cell fate inference in a snapshot of in vivo bone marrow hematopoiesis, allowing us to identify true outliers (in the sense of lineage commitment), demonstrating the effectiveness of this approach. Moreover, the results also highlight limitations of low-dimensional representations of high-dimensional data, as the B- and T-like cells detected by MultistageOT are poorly resolved in the original force-directed graph embedding published by Dahlin et al. (17).

Recent advances in spatial transcriptomics now allow for profiling of gene expression in spatially resolved tissues, achieved by giving the same spatial coordinates to multiple cells that colocalize in the same array feature (28). As spatial transcriptomics techniques are approaching single-cell resolution, we see potential in future works to leverage this type of data to guide the trajectory inference. This could be achieved by penalizing transport couplings between cells depending on physical distance, thereby enabling spatially informed trajectory inference. In particular, MultistageOT could incorporate spatial information with an extended transport cost on the form $L=\left(1-\alpha \right)L_{\text{orig}}+\alpha L_{\text{ST}}$, where $L_{\text{orig}}$ is the original MultistageOT cost, $L_{\text{ST}}$ is a spatial cost term based on spatial transcriptomics data and α∈[0,1] controls the relative weight given to the spatial data. For example, a natural choice for $L_{\mathrm{ST}}$ would be the Euclidean (physical) distance between the spatial coordinates of cells. We foresee that such a framework could work well in systems not dominated by circulating cells, where the spatial coordinates of cells carry meaningful information with respect to their development, such as *A. thaliana* root tip development.

Finally, applying MultistageOT to predict cells belonging to a previously described, but rare, bipotent progenitor population allowed us to establish the transcriptional composition of the predicted cells at the single-cell level. Recent advances in methods enabling paired transcriptome and surface protein measurements for individual cells [e.g., CITE-seq (29)] in principle enables analogous characterization of differentially expressed surface markers of the predicted cells. In future work, we thus hope to see utilization of multiomics data for model-guided inference of fluorescence-activated cell sorting strategies to isolate the progenitors of interest. Cell fate assays are then used to investigate and validate the progenitors’ predicted fate potential.

Taken together, we believe that MultistageOT has the potential to be utilized in the discovery, characterization, and validation of uncharted differentiation stages of any developmental process marked by well-defined beginning and end states.

## Materials and Methods

### Implementation.

MultistageOT solves a multistage optimal transport problem on cellular state space to minimize a total transition cost (see *SI Appendix*, *Supplementary Note* for details). Transition costs between cell states were computed as the squared Euclidean distances between data points. In applying MultistageOT to scRNA-seq data, we applied the preprocessing steps described in *SI Appendix*, *Supplementary Methods* and then computed the squared Euclidean distances on the PCA transformed data, keeping the first 50 principal components.

All results were computed using a MacBook Pro 14 with an M1 Pro chip and 32 GBs of memory. This allowed us to compute the MultistageOT solution on datasets of around N≈11,000 cells using *SI Appendix*, Algorithm 1. Hence, for the larger datasets from Dahlin et al. (17) and Weinreb et al. (14), we partitioned the data into uniformly subsampled disjoint subsets of roughly that size and applied the method independently on each subset.

#### Number of transport stages.

The number of transport stages sets the resolution in the analysis of temporal progression. For all scRNA-seq datasets, we solved the MultistageOT problem using T−1=20 intermediate transport steps to balance computational efficiency and resolution.

#### Specifying initial and terminal states.

Specifying initial and terminal differentiation states in MultistageOT grounds the model in well-established biology.

For the Paul et al. (13) data, in the absence of prior knowledge about the number of cells represented in each mature blood cell lineage, we picked $n_{0}=3$ initial cell states and $n_{F}=24$ terminal cell states (3 in each of the eight lineages) in regions of the UMAP expressing lineage associated markers (Fig. 5*A*). The initial states were chosen to lie in a region of the UMAP marked by high *Mpl* expression as well as low *Pf4* expression.

For the Weinreb et al. (14) lineage tracing data, we selected a total of $n_{0}=120$ initial states, and $n_{F}=6972$ terminal states (Fig. 6*A*). Since the data were partitioned into 12 subsets, solved independently, we selected exactly 10 initial states and 581 terminal states in each subset. The initial states comprise a subset of an HSC population found by only considering cells annotated by Weinreb et al. (14) as “Undifferentiated,” in a region of the SPRING-plot embedding also marked by high expression of *Procr* and *Cd34*. Terminal states were chosen to lie on the “tips” of the spring-graph embedding, representing each of the annotated mature blood cell lineage. We based the number of terminal states represented in each fate on the relative frequency of annotated cells in each fate, and then normalized this number of terminal states so that the smallest fate had one cell represented.

For the Dahlin et al. (17) data, we picked a total of $n_{F}=252$ terminal cell states, in regions of the UMAP expressing lineage associated markers (*SI Appendix*, Fig. S6) [40 in each lineage except for Eosinophils (12), Mast cell (20), and Baso (20)]. We ensured that any terminal cell states in $X_{F}$ had nearby intermediate cell states in X so as to not cause numerical instability. We picked $n_{0}=40$ initial states in a region of the UMAP marked by high expression of the gene *Procr*.

#### Cost of transporting mass to auxiliary cell states.

When applying the model extension with auxiliary cell states on the scRNA-seq data in ref. 17, we used a fixed cost Q=3.25 of transporting mass to any of the auxiliary states. This cost was chosen as conservatively as possible so as to not needlessly affect the overall solution (see *SI Appendix*, Fig. S7*A* for a representative plot of the distributions of the costs on which Q=3.25 is marked). As such, it corresponds to the highest value that we found prevented the iterates from generating division-by-zero errors.

To gauge the effects of the auxiliary cell state extension, we applied it to the synthetic dataset and found it to produce robust results over a range of *Q*-values (*SI Appendix*, Fig. S14). We also tested it on a partition of the lineage tracing dataset (14) with Q=2 and found that too produced similar results (*SI Appendix*, Table S3).

#### Convergence.

We monitored the convergence of the Sinkhorn algorithm (*SI Appendix*, *Supplementary Note*) and terminated the iterations when the maximum over all dual variable updates and absolute constraint deviations were less than the chosen tolerance level $\tau =10^{-4}$ for the data from Paul et al. (13) and Dahlin et al. (17) and $\tau =5\cdot 10^{-4}$ for the data from Weinreb et al. (14) (tolerance level was increased slightly to lower compute time on this bigger dataset).

#### Regularization parameter.

MultistageOT solves an entropy-regularized multistage optimal transport problem. The level of regularization is set by the user and is therefore a hyperparameter in MultistageOT, affecting the level of diffusion in the optimal transitions (*SI Appendix*, *Supplementary Note*). It is important to note that the relative influence of the regularization parameter, denoted *ϵ*, will in general be affected by the scale of the data. Therefore, in our implementations, following Schiebinger et al. (2), we normalized the cost matrices by dividing the elements by the median of all costs. This made *ϵ* less dependent of the original scale of the input data and it was thus on a comparable scale between different datasets. See *SI Appendix*, Table S1 for values used in our implementations.

To achieve a regularization parameter in the desired range, we nested our algorithm in a proximal point scheme (*SI Appendix*, Supplementary Note, section 4.3). This required us to set a starting value for the regularization parameter. This starting value was chosen as low as possible to limit the number of outer proximal point iterations needed to reach a sufficiently low effective regularization parameter. Note that, even when normalizing the cost matrices, these starting values differ slightly depending on specific data distribution within each dataset (e.g., due to the presence of outliers that correspond to many large distances to other cells). For example, the median transition cost in the synthetic dataset with outliers is ∼1.1 times larger than the median in the original data without outliers. To ensure that the optimal transitions between the nonoutlier cells would be comparable in the two settings assessed in *SI Appendix*, Fig. S8*E*, the regularization parameter was scaled accordingly. We refer to *SI Appendix*, Table S1 for a summary of the initial and final regularization parameter values for each dataset.

### Use of Generative AI.

GPT-4o was used for minor text edits in preparing the text for the manuscript. Any suggestions offered by GPT-4o that ended up incorporated into the text have been carefully reviewed by the authors. Thus, we take full responsibility for the entire text in the manuscript.

## Supplementary Material

Appendix 01 (PDF)

## Data Availability

All code, data, and scripts used in producing the results have been deposited on Zenodo (DOI: 10.5281/zenodo.17233337). Raw and processed data from Weinreb et al. (14) is available at the Gene Expression Omnibus (GEO) (Series GSE140802) as well as from https://github.com/AllonKleinLab/paper-data/tree/master/Lineage_tracing_on_transcriptional_landscapes_links_state_to_fate_during_differentiation. The raw data from Dahlin et al. (17) are available at GEO (Series GSE107727). The data from Paul et al. (13) are available through Scanpy. We also provide access to the synthetic data and the Paul et al. (13) data through our public GitHub repository (https://github.com/dahlinlab/MultistageOT). All processed data can be provided upon request. MultistageOT is released under the BSD-3-Clause license, with code available at https://github.com/dahlinlab/MultistageOT, including user-friendly Jupyter Notebook scripts. Previously published data were used for this work (13, 14, 17, 19, 21).
