# Supplementary material for: MultistageOT: Multistage optimal transport infers trajectories from a snapshot of single-cell data
Source: Proc Natl Acad Sci U S A. 2025 Dec 11;122(50):e2516046122. doi: 10.1073/pnas.2516046122 (PMC12718350; doi:10.1073/pnas.2516046122)
Supplement: Supplementary file 1 — Appendix 01 (PDF) [file pnas.2516046122.sapp.pdf]

PNAS

Supporting Information (SI) Appendix for:

MultistageOT: Multistage optimal transport infers trajectories from a snapshot of single-cell data

Magnus Tronstad[1*], Johan Karlsson[2†*], and Joakim S. Dahlin[1†*]

[1]Department of Medicine Solna, Karolinska Institutet, and Center for Molecular Medicine, Karolinska University Hospital, Stockholm, Sweden
[2]Department of Mathematics, KTH Royal Institute of Technology, Stockholm, Sweden

[†]These authors jointly supervised the work.
[*]Corresponding authors. Emails: magnus.tronstad@ki.se, johan.karlsson@math.kth.se, and joakim.dahlin@ki.se

November 27, 2025

Contents

This *SI Appendix* contains (in order):

- Supplementary Tables
- Supplementary Figures
- Supplementary Methods
- Supplementary Note (Mathematical formulation of MultistageOT)

Supplementary Tables

Table S1: Regularization parameter values. Note that ϵ_0 is the initial value in the proximal point scheme, and ϵ' is the effective regularization parameter value, corresponding to having obtained the optimal transport plans with an uninformed prior matrix of all-ones in the entropy-regularization (see section 4.3 in Supplementary Note). The parameter values for the synthetic data with and without outliers correspond to the results in Fig. 7B and Fig. 4I, respectively.

**Data set**	**Initial** ϵ_0	**Effective**, ϵ'
Synthetic data:		
Without outliers	0.015	0.0042
With outliers	0.038	0.0038
Paul et al. (2015):	0.055	0.0055
Dahlin et al. (2018):		
Partition 1	0.013	0.0045
Partition 2	0.013	0.0043
Partition 3	0.013	0.0043
Partition 4	0.013	0.0045
Weinreb et al. (2020):		
Partition 1	0.03	0.0057
Partition 2	0.03	0.0060
Partition 3	0.03	0.0055
Partition 4	0.03	0.0060
Partition 5	0.03	0.0055
Partition 6	0.03	0.0059
Partition 7	0.03	0.0060
Partition 8	0.03	0.0057
Partition 9	0.03	0.0060
Partition 10	0.03	0.0060
Partition 11	0.03	0.0066
Partition 12	0.03	0.0058
Shahan et al. (2022):	0.01	0.005
Weng et al. (2024):	0.03	0.005

Table S2: Weights, b_k used in the Inverse Distance Weighed (IDW) model for estimating cell fate probabilities

Fate, k:	Weight, b_k:
Neutrophil	0.3789
Monocyte	0.3268
Baso	0.1730
Mast	0.0440
Meg	0.0357
Lymphoid	0.0146
Erythroid	0.0117
Eos	0.0095
Ccr7_DC	0.0041
pDC	0.0017

Table S3: Change (measured by total variation distance (TV)) when recomputing the cell fate probabilities with the extended MultistageOT model with auxiliary cell states on day 2 cells in a partition of the Weinreb et al. [1] data set, for three different values for the fixed transport cost, Q.

Cost, Q	Mean(max) TV
1	0.047(0.25)
2	0.0092(0.054)
3.25	0.0091(0.054)

References

[1] Weinreb, C., Rodriguez-Fraticelli, A., Camargo, F. D. & Klein, A. M. Lineage tracing on transcriptional landscapes links state to fate during differentiation. *Science* **367**, eaaw3381 (2020).

Supplementary Figures

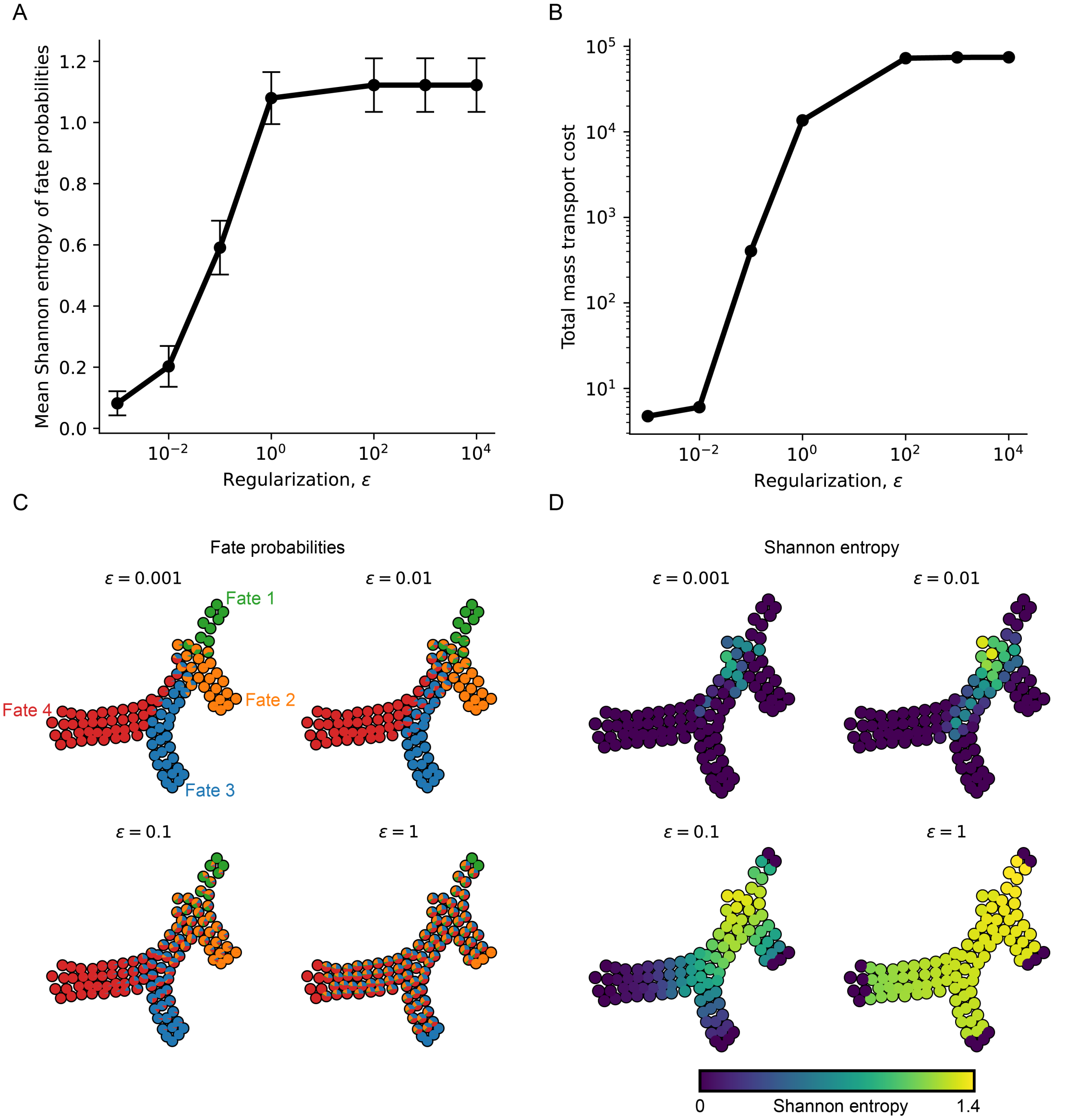

Fig. S1: Overall Shannon entropy increases monotonically with increasing regularization parameter values. (A) Mean Shannon entropy of fate probabilities, averaged over all cells in the MultistageOT solution corresponding to the data set used in Fig. 4, plotted against increasing values of the regularization parameter, ϵ. Errorbars correspond to approximate 95% confidence intervals. (B) Total cost of transports in the MultistageOT solution plotted against increasing values of the regularization parameter, ϵ. (C) Cell fate probabilities, represented by pie charts, for different values of ϵ. (D) Shannon entropy of the cell fate probabilities for each cell shown in (C), for different values of ϵ.

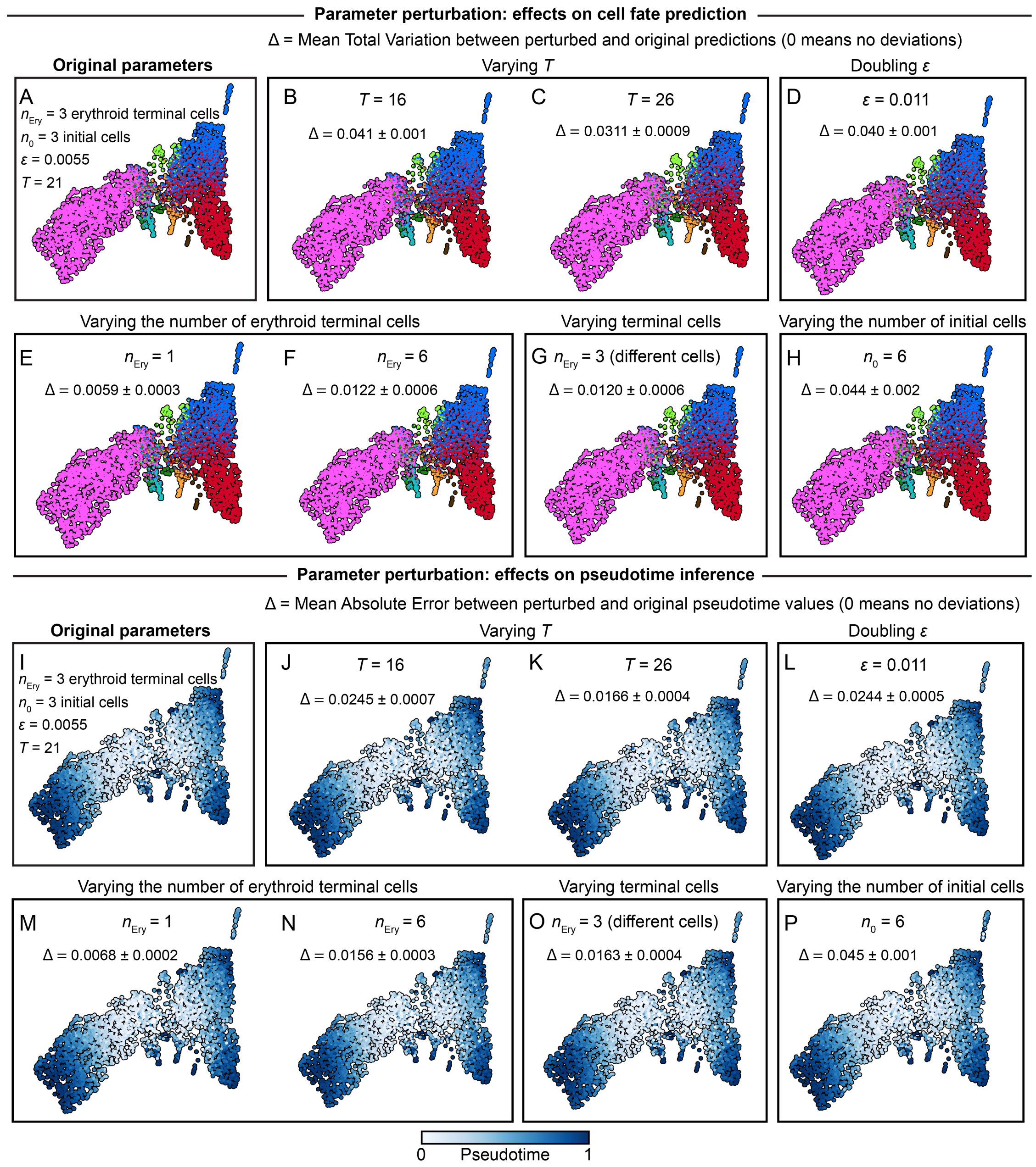

Fig. S2: Sensitivity analysis. (A-H) Results for cell fate probability estimates, where Δ represents mean total variation between perturbed and original predictions. (I-P) Results for pseudotime estimates, where Δ represents mean absolute error between perturbed and original predictions. Panels A and I show the original results on the Paul et al. data [1] corresponding to Fig. 5 for reference. Panels B, C, J, and K correspond to varying T. Panels D and L correspond to doubling the regularization parameter value. Panels E, F, M, and N correspond to varying the number of erythroid cells. Panels G and O correspond to specifying a different set of terminal erythroid cells. Panels H and P correspond to doubling the number of initial cells.

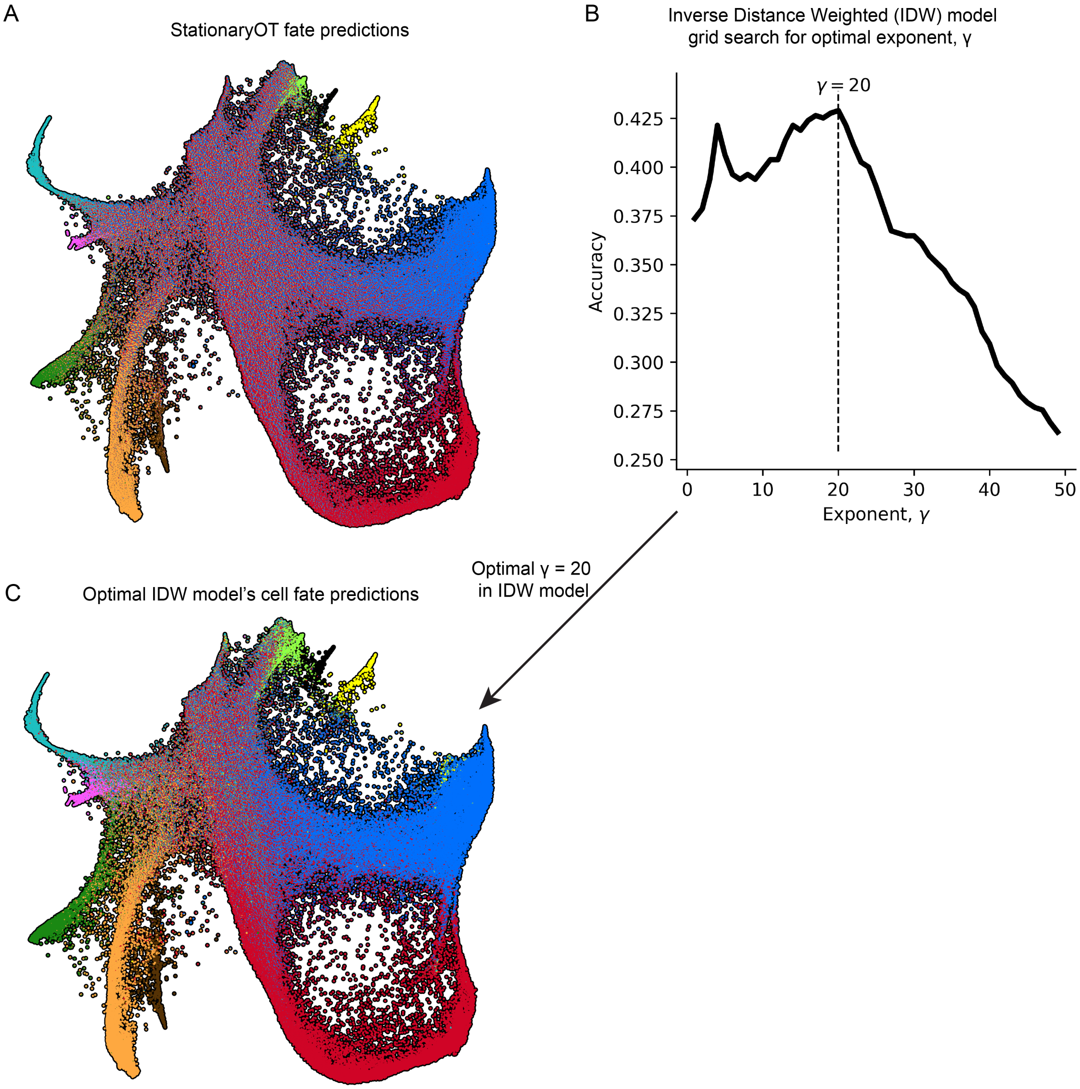

Fig. S3: (A) StationaryOT's predictions of cell fate probabilities for 130884 cells in the Weinreb et al. [2] data set. (B) The inverse distance weighted (IDW) model (see Methods). We used the ground truth fates to optimize the parameter with respect to "dominant fate accuracy". (C) IDW model's predictions of cell fate probabilities for 130884 cells in the Weinreb et al. data set.

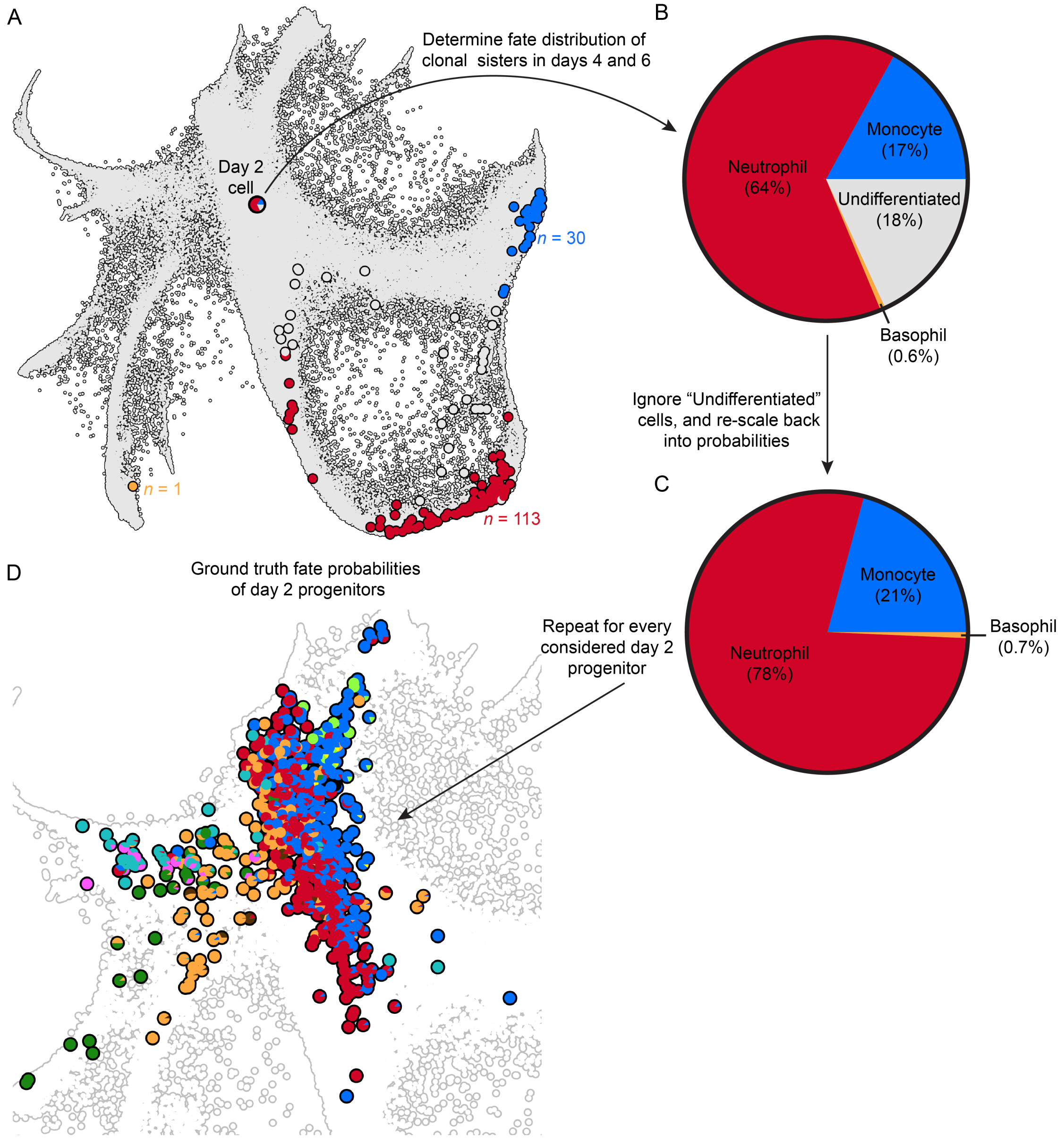

Fig. S4: Establishing "ground truth" fate probabilities of day 2 progenitor cells using fate distribution of their clonal sisters in later time points (days 4 and 6). (A) Example of a day 2 progenitor cell and its clonal sisters in days 4 and 6. (B) The distribution of clonal sisters (basophil: 1, neutrophil: 113, monocyte: 30, undifferentiated: 32) can be normalized to induce a probability distribution over the different fates. (C) We ignore the "Undifferentiated" cells, and then rescale the relative frequencies of clonal fates so that they sum to unity. (D) We repeat this for every selected day 2 progenitor that matched our inclusion criteria (see Methods). Performance metrics were then obtained by comparing our model's estimated cell fate probabilities predictions to these empirical fate probabilities.

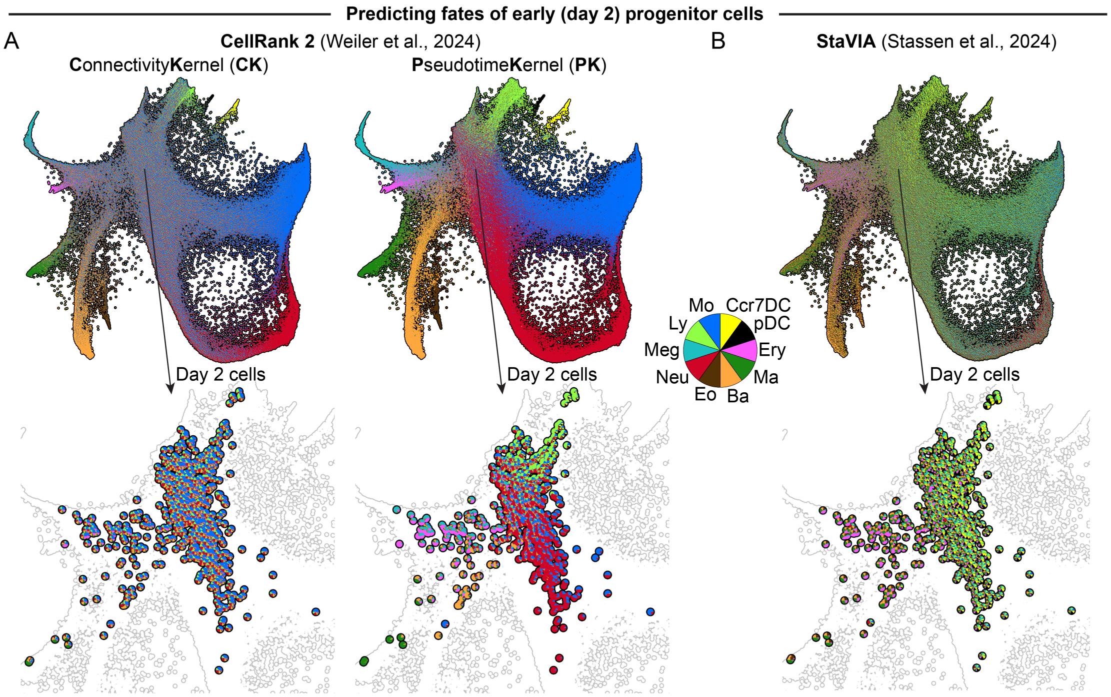

C

Performance metrics (Day 2 cells)

Model	Mean TV (± std.error)	Dominant fate accuracy	Total fate accuracy
MultistageOT	0.50 ± 0.009	54%	56%
CellRank 2 (CK)	0.71 ± 0.005 (p<0.001)	37%	47%
CellRank 2 (PK)	0.57 ± 0.009 (p<0.001)	43%	49%
StaVIA	0.91 ± 0.004 (p<0.001)	3%	3%

Mean TV (lower values are better), Dominant fate accuracy (higher values are better), and Total fate accuracy (higher values are better). *p*-values indicate significance of the difference in mean TV relative to MultistageOT based on a two-sided Mann-Whitney *U*-test.

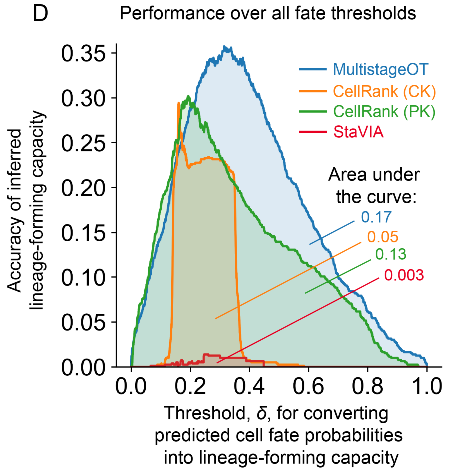

Fig. S5: Benchmarking accuracy of trajectory inference methods in predicting fates of progenitor cells in Weinreb et al. [2]. (A) Cell fate probabilities estimated with CellRank2 [3] (left: with ConnectivityKernel, right: with PseudotimeKernel). (B) Cell fate probabilities estimated with StaVIA [4]. (C) Performance metrics for each method. The p-values correspond to comparisons to MultistageOT in a Mann-Whitney U-test (two-sided). (D) Predictive performance for each model (including MultistageOT for reference) after assigning a cell to fates with predicted fate probability above a threshold, denoted δ. The plot shows performance for each threshold (horizontal axis). Vertical axis reports the fraction of cells whose model-based assignment of fate potential matches the observed clonal sister fates. Abbreviations: Meg (megakaryocyte), Ery (erythroid), Ma (mast cell), Ba (basophil), Eo (eosinophil), Neu (neutrophil), Mo (monocyte) pDC (plasmacytoid dendritic cell), Ccr7DC (Ccr7^{+} DC) and Ly (lymphoid).

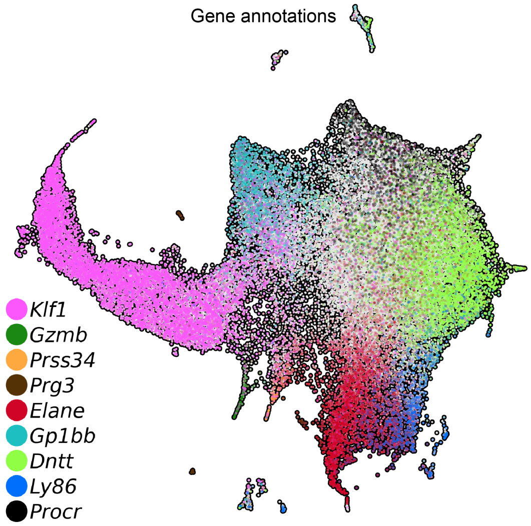

Fig. S6: Layered gene expression (log(x+1)-transformed) plot showing the orientation of different cell lineages and hematopoietic stem cells: erythroid (*Klf1*), mast cell (*Gzmb*), basophil (*Prss34*), eosinophil (*Prg3*), neutrophil (*Elane*), megakaryocyte (*Gp1bb*), lymphoid (*Dntt*), monocyte (*Ly86*) and hematopoietic stem cells (*Procr*). Gene markers from Dahlin et al. [5].

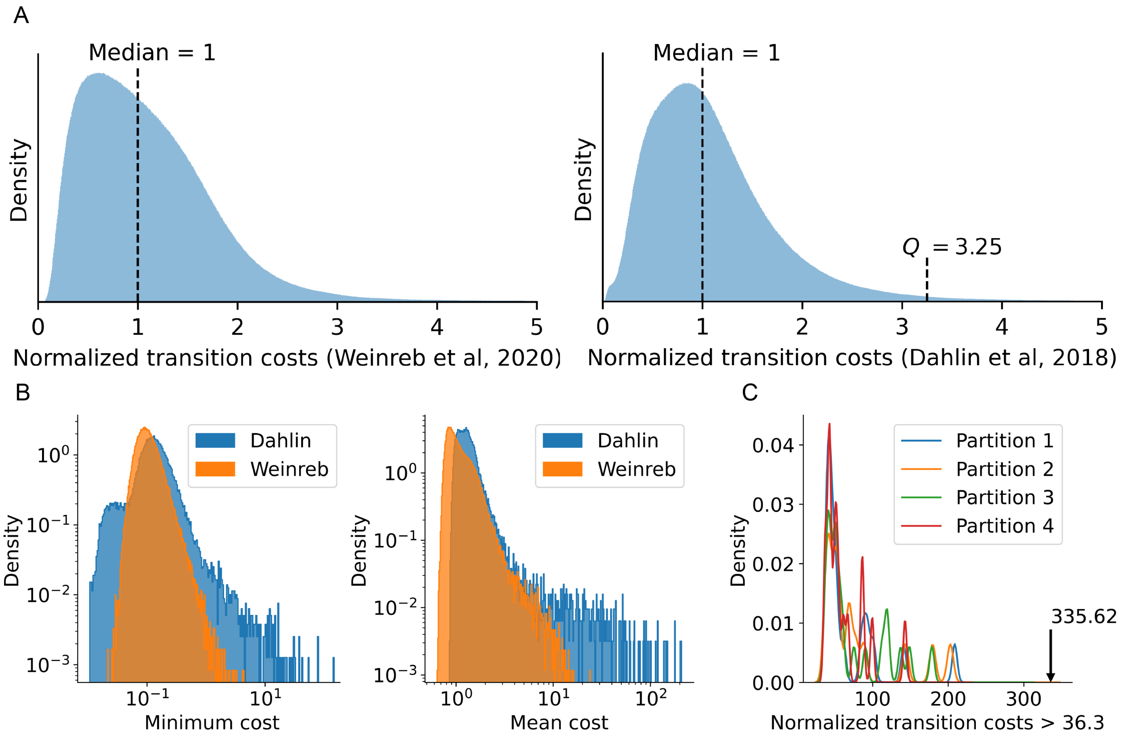

Fig. S7: (A) Representative distributions of transition costs (squared Euclidean distance) in the range [0,5] in Weinreb et al. [2] (left) versus Dahlin et al. [5] (right). The cost $Q = 3.25$, of transporting unit mass to the auxiliary states in the extended MultistageOT model (see Methods) is marked in the right plot. (B) Minimum (left) and mean (right) transition costs in the Dahlin et al. versus Weinreb et al. data sets. Note the log-scales. (C) Density plots of transition costs for each partition of the Dahlin et al. data (Supplementary Methods), considering only large costs above the maximum cost in the Weinreb et al. data set, which was 36.3. For reference, the arrow marks the highest transition cost in the Dahlin et al. data set.

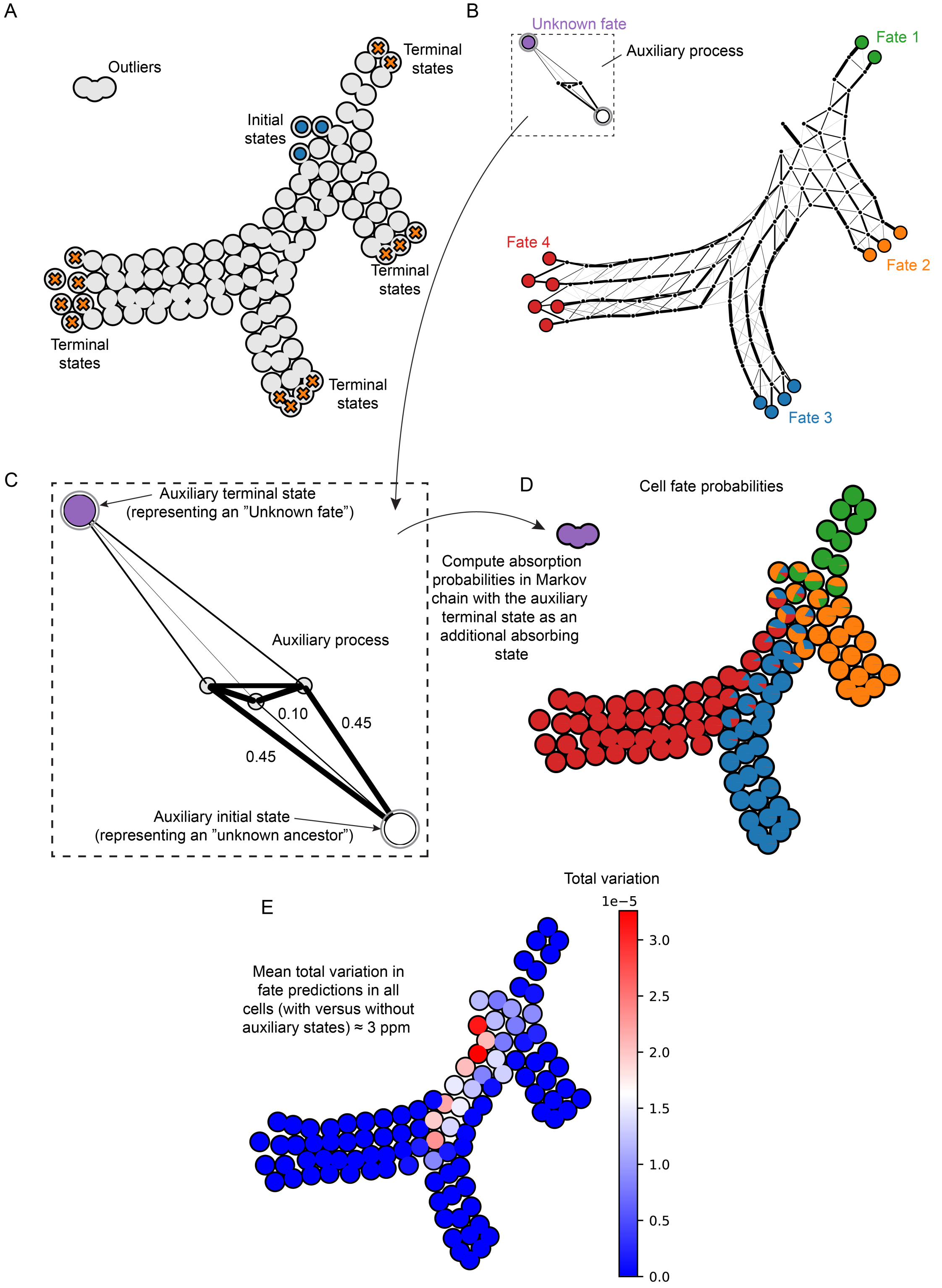

Fig. S8: Extended MultistageOT model with auxiliary states applied to the synthetic data set in Fig. 4, with the addition of outliers. (A) A cluster of three outlier cells was added in the top left corner of the data in Fig. 4. (B) We solved the MultistageOT problem with the added auxiliary cell states. The

plot visualizes the Markov chain transition probabilities between cells, based on the optimal transport solution (Methods). (C) In this scenario, the auxiliary states help model the development of the outlier cells as separate process: No connections were formed between the outliers and the cells in the main process. The auxiliary intermediate cell state was not utilized by any cell in the optimal transport solution and was therefore omitted in the visualization. Note: The auxiliary terminal and initial states (purple and white respectively) are here only included to visualize the transition probabilities; in general they do not possess geometric coordinates. (D) Including the auxiliary terminal state as an additional absorbing state in the Markov chain allows estimating the probability of each cell ending up in an "unknown fate", represented by the auxiliary terminal cell state. The probabilities are represented by pie charts, and purple wedges corresponds to this "Unknown fate". (E) The total variation distance for each non-outlier cell, quantifying the difference between the cell fate predictions shown in Fig. 4 (using the original MultistageOT formulation) and the cell fate predictions made with the extended MultistageOT model shown in subpanel (D).

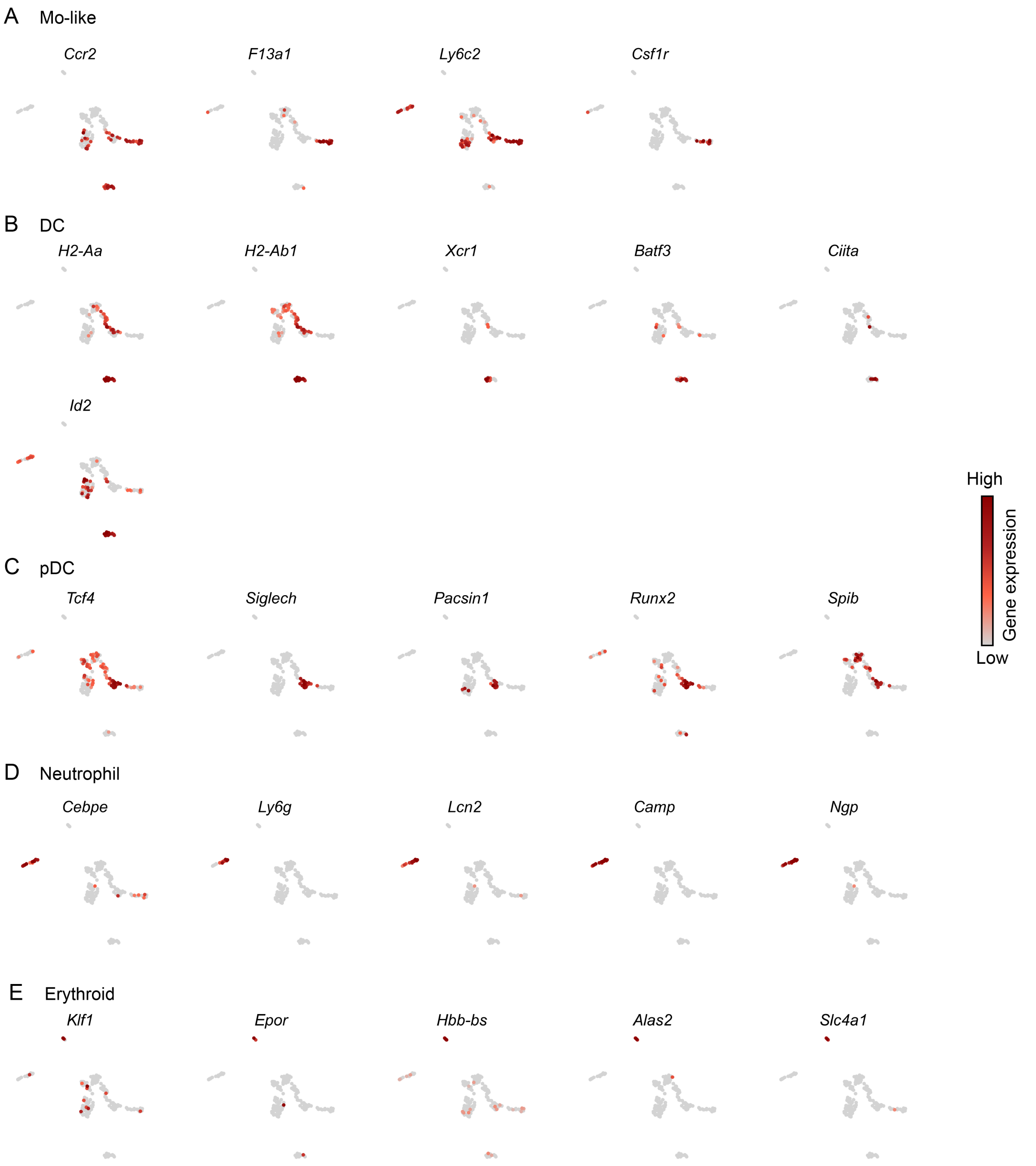

Fig. S9: The expression levels (log(x+1)-transformed) of lineage-associated gene markers used in annotating the candidate outliers identified by MultistageOT in the data set from Dahlin et al. [5]. UMAP embedding featuring only the candidate outliers. (A) Monocyte-like (Mo-like) markers from Mildner et al. [6] and Konturek-Ciesla et al. [7]. (B) DC (dendritic cell) markers from Lukowski et al. [8]. (C) pDC (plasmacytoid DC) markers from Lukowski et al. [8]. (D) Neutrophil markers from Konturek-Ciesla et al. [7] and Grieshaber-Bouyer et al. [9]. (E) Erythroid markers from An et al. [10] and Dzierzak & Philipsen [11].

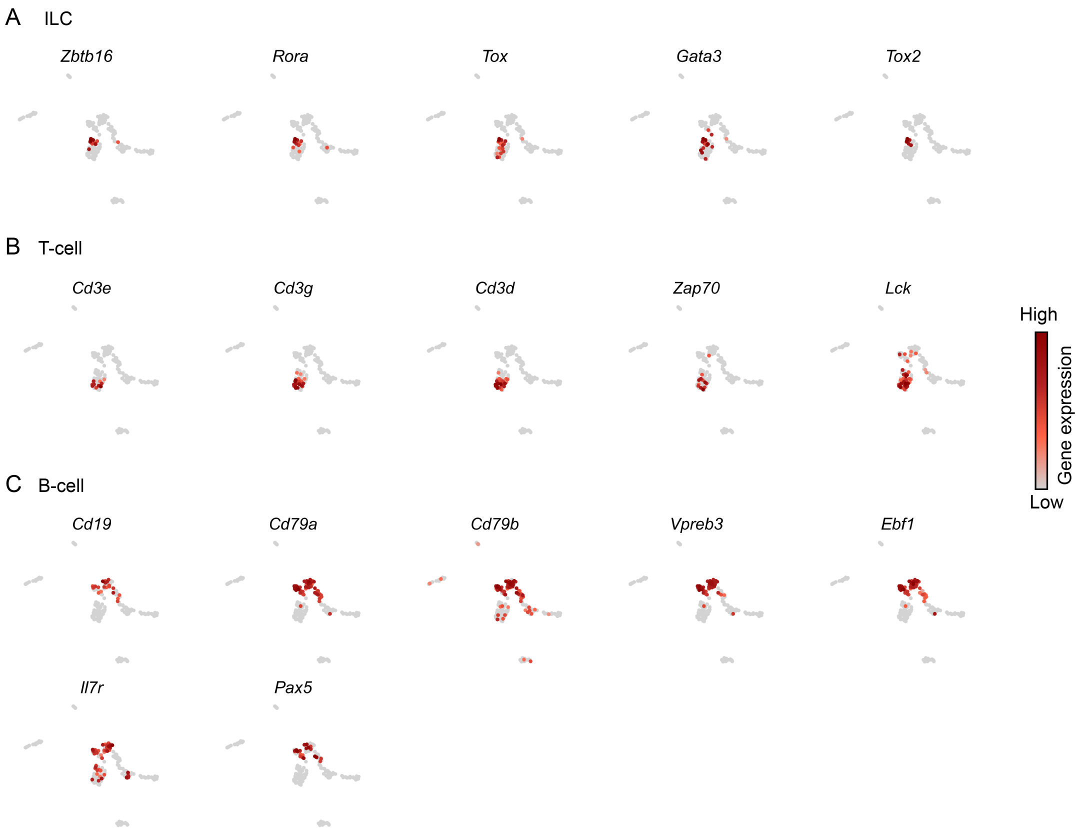

Fig. S10: The expression levels (log(x+1)-transformed) of lineage-associated gene markers used in annotating the candidate outliers identified by MultistageOT in the data set from Dahlin et al. [5]. UMAP embedding featuring only the candidate outliers. (A) ILC (innate lymphoid cell) markers from Seillet et al. [12]. (B) T-cell markers from Rothenberg et al. [13] and Rothenberg [14]. (C) B-cell markers from Rothenberg [14].

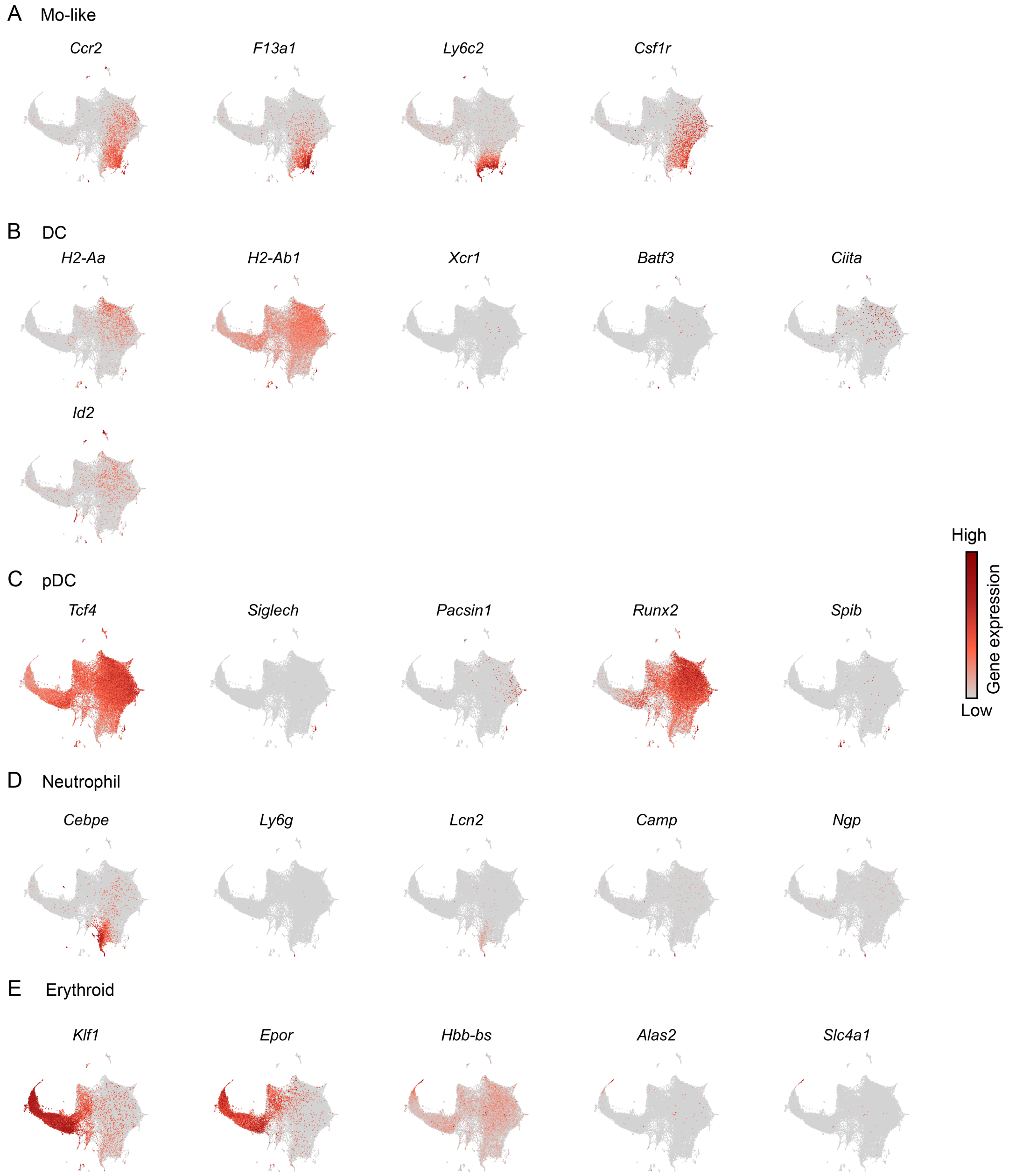

Fig. S11: The expression levels (log(x+1)-transformed) of lineage-associated gene markers used in annotating the candidate outliers identified by MultistageOT in the data set from Dahlin et al. [5]. (A) Monocyte-like (Mo-like) markers from Mildner et al. [6] and Konturek-Ciesla et al. [7]. (B) DC (dendritic cell) markers from Lukowski et al. [8]. (C) pDC (plasmacytoid DC) markers from Lukowski et al. [8]. (D) Neutrophil markers from Konturek-Ciesla et al. [7] and Grieshaber-Bouyer et al. [9]. (E) Erythroid markers from An et al. [10] and Dzierzak & Philipsen [11].

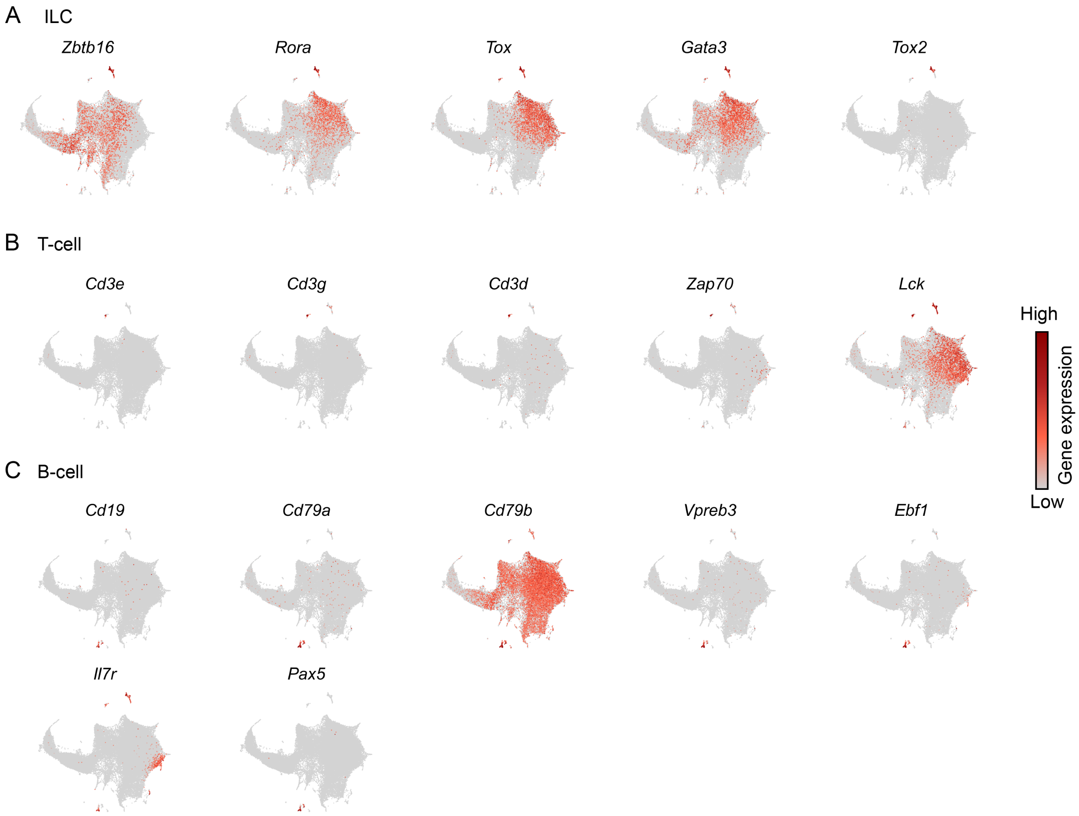

Fig. S12: The expression levels (log(x+1)-transformed) of lineage-associated gene markers used in annotating the candidate outliers identified by MultistageOT in the data set from Dahlin et al. [5]. (A) ILC (innate lymphoid cell) markers from Seillet et al. [12]. (B) T-cell markers from Rothenberg et al. [13] and Rothenberg [14]. (C) B-cell markers from Rothenberg [14].

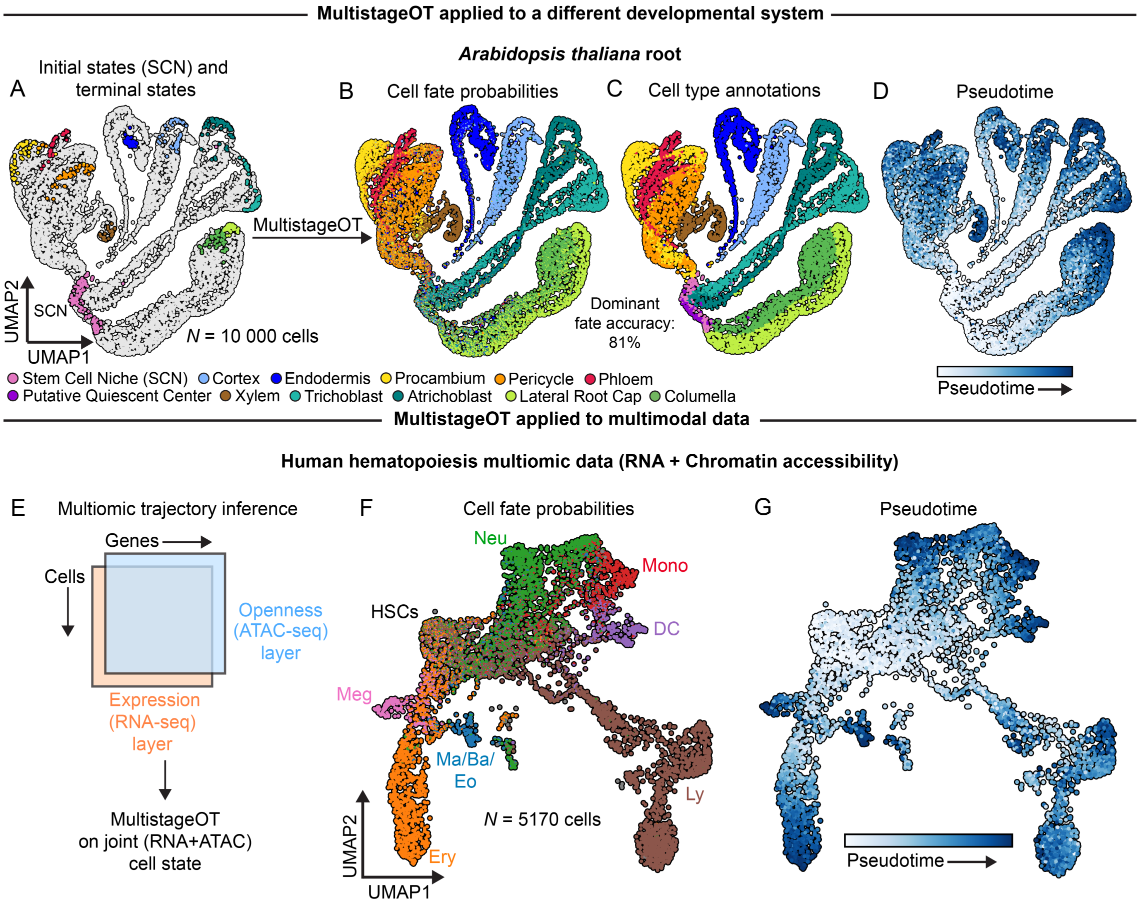

Fig. S13: MultistageOT applied to additional systems and modalities. (A) MultistageOT was applied to a subset of the *Arabidopsis thaliana* cell atlas of Shahan et al. [15], in which initial and terminal states were specified based on available meta data (*SI Appendix*, Supplementary Methods). (B) MultistageOT's inferred cell fate probabilities. (C) Cell type annotations from meta data (D) MultistageOT's inferred pseudotemporal ordering. (E) Multiome data [16] used as input to MultistageOT. (F) MultistageOT's inferred cell fate probabilities. Unknown fate is colored with grey (we used a fixed cost $Q = 2$ to transport mass to auxiliary cell states on this data set). Abbreviations: Meg (megakaryocyte), Ery (erythroid), Ma (mast cell), Ba (basophil), Eo (eosinophil), Neu (neutrophil), Mono (monocyte), Ly (lymphoid), DC (dendritic cell), HSCs (hematopoietic stem cells). (G) MultistageOT's inferred pseudotemporal ordering.

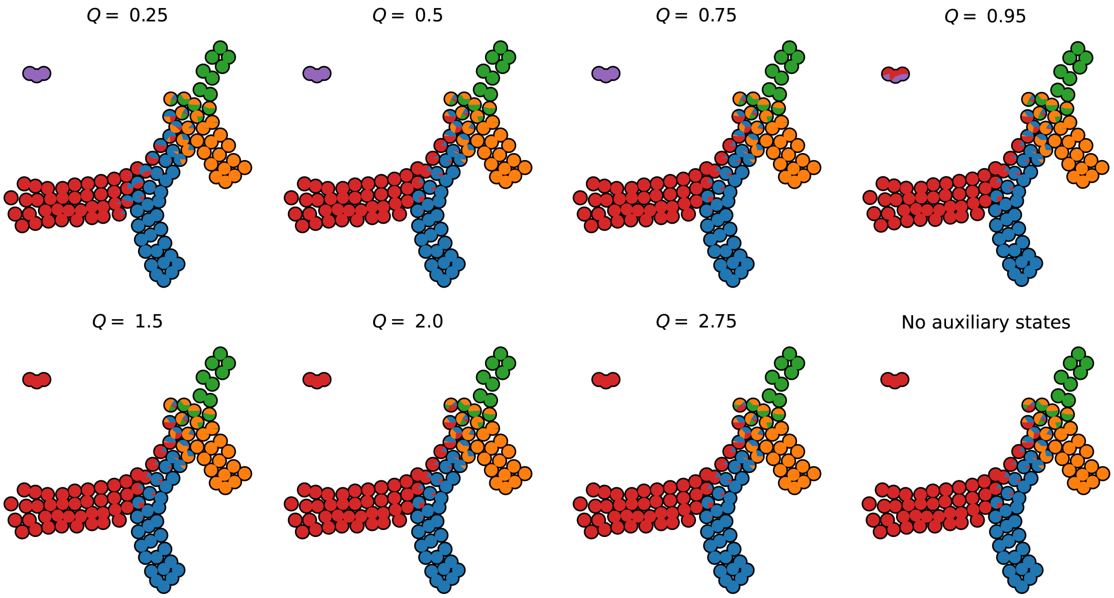

Fig. S14: Effects of varying the fixed cost Q of transitioning to the auxiliary cell states on the estimated cell fate probabilities. For reference, we include the cell fate probabilities when no auxiliary states are used (bottom rightmost plot). Low Q-values favors more utilization of the auxiliary states, reflected in the strong commitment of the outliers to terminate in the auxiliary terminal state (purple color) for $Q \in \{0.25, 0.5, 0.75\}$. Conversely, higher Q-values means less involvement of the auxiliary states. For the results in Fig. 7B we used $Q = 0.9$.

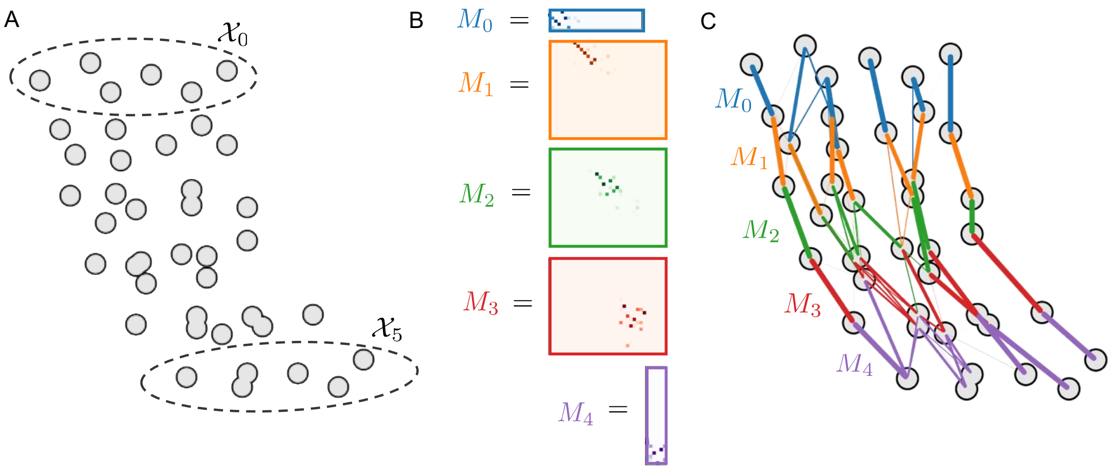

Fig. S15: Multistage problem (3.20) solved for a two-dimensional toy data set. (A) Initial and terminal states are specified as input to the model. (B) Problem (3.20) is solved with $\epsilon = 0.005$ and $T = 5$. The color intensity of a matrix element $(m_t)_{ij}$ in the transport plan matrices M_t, $t = 0, 1, 2, 3, 4$ is determined by the amount of mass sent from cell i to cell j in time step t. (C) Visualization of the optimal transport plans $M_0 = \tilde{M}_0$, $M_t = \begin{bmatrix}\tilde{M}_t & \hat{M}_t\end{bmatrix}$, $t = 0, 1, 2, 3,$ $M_4 = \hat{M}_4$. The width of a colored line connecting two cells is proportional to the amount of mass sent between the cells in the corresponding time step: $t = 0$ (blue); $t = 1$ (orange); $t = 2$ (green) ; $t = 3$ (red); $t = 4$ (purple). These mass transport plans can be interpreted in terms of transitions likelihoods.

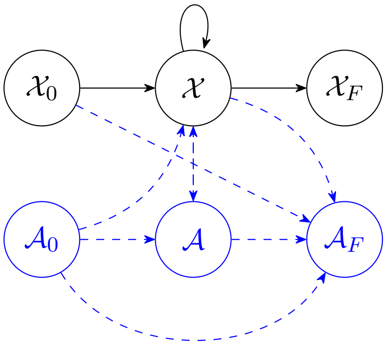

Fig. S16: Graph representation of our multistage optimal transport model of cell differentiation, together with the extension with added auxiliary states, $\mathcal{A}_0$ (initial), $\mathcal{A}$ (intermediate), $\mathcal{A}_F$ (terminal). The black graph represents the MultistageOT model without the extension (i.e., a collapsed version of the graph in Fig. 3) and the blue graph with dashed arrows represent the extension with auxiliary states (Supplementary Note).

References

[1] Paul, F. *et al.* Transcriptional heterogeneity and lineage commitment in myeloid progenitors. *Cell* **163**, 1663–1677 (2015).

[2] Weinreb, C., Rodriguez-Fraticelli, A., Camargo, F. D. & Klein, A. M. Lineage tracing on transcriptional landscapes links state to fate during differentiation. *Science* **367**, eaaw3381 (2020).

[3] Weiler, P., Lange, M., Klein, M., Pe'er, D. & Theis, F. Cellrank 2: unified fate mapping in multiview single-cell data. *Nat. Methods* **21**, 1196–1205 (2024).

[4] Stassen, S. V. *et al.* Stavia: spatially and temporally aware cartography with higher-order random walks for cell atlases. *Genome Biol.* **25**, 224 (2024).

[5] Dahlin, J. S. *et al.* A single-cell hematopoietic landscape resolves 8 lineage trajectories and defects in Kit mutant mice. *Blood* **131**, e1–e11 (2018).

[6] Mildner, A. *et al.* Genomic characterization of murine monocytes reveals C/EBPβ transcription factor dependence of Ly6C$^-$ cells. *Immunity* **46**, 849–862 (2017).

[7] Konturek-Ciesla, A. *et al.* Temporal multimodal single-cell profiling of native hematopoiesis illuminates altered differentiation trajectories with age. *Cell Rep.* **42**, 112304 (2023).

[8] Lukowski, S. W. *et al.* Absence of Batf3 reveals a new dimension of cell state heterogeneity within conventional dendritic cells. *IScience* **24**, 102402 (2021).

[9] Grieshaber-Bouyer, R. *et al.* The neutrotime transcriptional signature defines a single continuum of neutrophils across biological compartments. *Nat. Commun.* **12**, 2856 (2021).

[10] An, X. *et al.* Global transcriptome analyses of human and murine terminal erythroid differentiation. *Blood* **123**, 3466–3477 (2014).

[11] Dzierzak, E. & Philipsen, S. Erythropoiesis: development and differentiation. *Cold Spring Harb. Perspect. Med.* **3**, a011601 (2013).

[12] Seillet, C. *et al.* Deciphering the innate lymphoid cell transcriptional program. *Cell Rep.* **17**, 436–447 (2016).

[13] Rothenberg, E. V., Moore, J. E. & Yui, M. A. Launching the T-cell-lineage developmental programme. *Nat. Rev. Immunol.* **8**, 9–21 (2008).

[14] Rothenberg, E. V. Transcriptional control of early T and B cell developmental choices. *Annu. Rev. Immunol.* **32**, 283–321 (2014).

[15] Shahan, R. *et al.* A single-cell arabidopsis root atlas reveals developmental trajectories in wild-type and cell identity mutants. *Dev. Cell* **57**, 543–560 (2022).

[16] Weng, C. *et al.* Deciphering cell states and genealogies of human haematopoiesis. *Nature* **627**, 389–398 (2024).

Supplementary Methods

Data preprocessing

All single-cell RNA-sequencing data were preprocessed in Python 3.9 using the Scanpy module (version 1.9.3), unless otherwise stated.

Generation of synthetic data

For the simulated data in Fig. 4, we manually generated $N = 105$ data points in the unit square to reflect a simple differentiation process with known initial and terminal cell states, utilizing the online tool: `https://guoguibing.github.io/librec/datagen.html`. This synthetic dataset was generated to illustrate the method's core concepts in an accessible, non-technical manner, and to ensure correct implementation.

Paul et al. (2015) *in vivo* snapshot of mouse hematopoiesis

The Paul et al. [1] data is integrated in the Scanpy python module, and was loaded using the function `scanpy.datasets.paul15`. We performed the preprocessing steps outlined in the Scanpy tutorial in `https://scanpy-tutorials.readthedocs.io/en/latest/paga-paul15.html`. In particular, the data was converted to float64 (as per the recommendations in the tutorial) and finally preprocessed using the function `scanpy.pp.recipe_zheng17`. This procedure retained 1000 highly expressed genes and then normalized cells by total counts, after which $\log(x+1)$-transformation and standardization to unit variance and zero mean was performed.

Weinreb et al. (2020) *in vitro* lineage tracing in mouse hematopoiesis

The lineage tracing data from Weinreb et al. [2] was downloaded as a normalized count table (L_1-normalized counts) from the authors' Github repository: `https://github.com/AllonKleinLab/paper-data/tree/master/Lineage_tracing_on_transcriptional_landscapes_links_state_to_fate_during_differentiation`. To start, the data frame contained 25289 genes and 130887 cells. Highly variable genes were selected using Scanpy's `highly_variable_genes` function (`flavor='seurat', min_mean=0.001, max_mean=5, min_disp=0.05`) leaving us with a total of 10220 genes. The data was then $\log(x+1)$-transformed and scaled to unit variance and zero mean. PCA was performed on this preprocessed data keeping the first 50 principal components. Finally, we randomly omitted three data points from the data set in order to have the total number of cells divisible by 12, and then created a partition of 12 disjoint subsets of the data, each of size 10907. MultistageOT was applied to each partition independently, and the results were then pooled when creating the 2D visualizations of pseudotime and cell fate probabilities in Fig. 6.

Dahlin et al. (2018) *in vivo* snapshot of mouse hematopoiesis

Data from Dahlin et al. [3] was downloaded from `http://gottgens-lab.stemcells.cam.ac.uk/adultHSPC10X/`. Doublets were removed by referencing the doublet score file in

the publicly available data. Cells were removed based on the following critera: having more than 10% mitochondrial reads, having the total number of reads ± 3 standard deviations from the mean (in log-scale) and having fewer than 500 genes expressed. Genes expressed in fewer than 3 cells were then removed. The resulting gene expression data frame featured 17633 genes in 44802 cells. The data was normalized by total counts so that each cell's expression levels summed to 10^4. Subsequently, we identified 5033 highly variable genes based on using Scanpy's `filter_genes_dispersion`-function (with parameters `log=True, flavor='seurat', min_mean=0.001, max_mean=5, min_disp=0.05, copy=True`). The remaining genes were then $\log(x+1)$-transformed and scaled to unit variance and zero mean. Finally, genes were correlated against a reference of cell cycle genes provided by Dahlin et al. [3], keeping only genes with with 0.2 or lower Pearson's correlation coefficient. This removed a total of 368 genes. At this stage, the data featured a total of 4665 genes in 44802 cells. PCA was performed on this processed data, keeping the first 50 principal components. Finally, a UMAP was created by first computing a neighbour graph with `scanpy.pp.neighbors`, using 9 nearest neighbors and the 50 first principal components, and then calling `scanpy.tl.umap` with `min_dist`-parameter to 0.4. We randomly removed two data points to make the total number of points divisible by four, and split the data set into four disjoint partitions, each of size 11200. MultistageOT was then applied to each partition independently.

Shahan et al. (2022) *Arabidopsis thaliana* root

Processed and annotated data were downloaded from the Github repository of [4] (`https://github.com/zsteve/StationaryOT`). This processed data features 10000 cells and 2000 genes and is a subset of the *Arabidopsis thaliana* root atlas of Shahan et al. [5]. A PCA analysis was performed on these 2000 genes, and the projection of the data onto the 50 first principal components was given as input to MultistageOT.

Weng et al. (2024) *in vivo* multiomic snapshot of human hematopoiesis

We downloaded "GSE219106_Young1_HSPC" from GEO (accession number: GSE219106).

To process the ATAC fragment file, we applied the SnapATAC2 [6] (version 2.8.0) pipeline, including the following QC and preprocessing steps. We performed TSS enrichment scoring and then called their `filter_cells` function with `min_counts` = 5000, `min_tsse` = 8, `max_counts` = 100,000. We then called `add_tile_matrix` followed by `select_features` that selected the 500,000 most accessible features across all cells. Finally, we called their `make_gene_matrix` function to create a cell-by-gene activation matrix, which was row normalized to 10,000 total counts for each cell and then $\log(x+1)$ transformed.

The RNA expression data was processed with the following steps. First, Scrublet (version 0.2.3) was run—removing 770 predicted doublets. We then performed QC and preprocessing steps with Scanpy (version 1.11). Specifically, we kept cells expressing less than 10% mitochondrial genes and more than a 100 different genes, and we kept genes expressed in at least 3 cells. We selected 10,000 highly variable genes using `scanpy.pp.highly_variable_genes` with `flavor = 'seurat'`. Next, the data was row normalized to 10,000 total

counts for each cell and then $\log(x + 1)$ transformed. After this preprocessing the RNA data comprised 6600 cells and 10,000 genes.

To define a multiomic-based state as input to MultistageOT, we intersected the RNA and ATAC data, choosing the cells and genes that were featured in both data sets. We also filtered some genes corresponding to zero variance. Note that this gave us 5170 cells and 7495 genes featured in both data frames, corresponding to paired gene expression and gene accessibility estimates. We performed separate PCA analyses on the RNA and ATAC gene data, keeping the 50 first principal components for both modalities. A UMAP visualization was then computed based on the RNA expression data. First, `scanpy.pp.neighbors` was called with `n_pcs = 50` and `n_neighbors = 30` and then `scanpy.tl.umap` was called with `min_dist = 0.3` and the rest left to default parameters. Finally, to define the input to MultistageOT, we concatenated the two PCA embeddings into an expanded cell state. In particular, if $z \in \mathbb{R}^{50}$ is the ATAC modality's PCA embedding, and $w \in \mathbb{R}^{50}$ is the RNA modality's PCA embedding, then we define the expanded cell state s as the concatenation $s = (z^T, w^T)^T \in \mathbb{R}^{100}$.

Implementation for supplementary data

MultistageOT was applied to the data from Shahan et al. [5] and Weng et al. [7]. In monitoring MultistageOT's convergence of the Sinkhorn algorithm (*SI Appendix*, Supplementary Note), we used a threshold $\tau = 10^{-4}$ for the maximum dual variable updates and absolute constraint deviations for both data sets. For the Shahan et al. [5] data, initial states were defined as the $n_0 = 165$ cells having a "Stem Cell Niche" cell type annotation (*SI Appendix*, Fig. S13A). Following the Github script of Zhang et al. [4], we assigned terminal states as the 5% most differentiated cells based on the "`consensus.time`"-variable in the meta data (excluding cells annotated as "Putative Quiescent Center" or "Stem Cell Niche") corresponding to a total of $n_F = 492$ terminal cells (*SI Appendix*, Fig. S13A). For the Weng et al. [7] data, we chose $n_0 = 15$ initial cells and a total of $n_F = 105$ terminal cells (15 terminal cells for each of the mature cell lineages) based on RNA expression of established gene markers: *AVP* for HSCs [8], *HDC* and *CLC* for mast cell/basophil/eosinophil-like cells, *KLF*1 and *HBB* for erythroid cells, *ELANE* and *AZU*1 for neutrophils, *LYZ* and *ANXA*2 for monocytes, *SPIB* and *IRF*8 for dendritic cells, *DNTT* for lymphoid cells, and *ITGA*2*B*, *PF*4 and *VWF* for megakaryocyte [9].

Statistical tests

In comparing the distribution of pseudotime across experimental time points (Fig. 6G), the Mann-Whitney U-test (two-sided) in Scipy (version 1.11.0) was used. The group sizes were 28249 cells in day 2, 48497 cells in day 4, and 54138 cells in day 6. In benchmarking performance of predicting day 2 progenitor fates (795 cells) (Fig. 6J), the Mann-Whitney U-test (two-sided) was used. For the differential expression analysis (Fig. 7K), p-values were computed with the Mann-Whitney U-test (two-sided), comparing the 35 BMCP-like cells to all other cells (44765).

Downstream analyses

Let $\mathcal{D} = \{x^{(i)} \mid i = 1, \ldots, N\}$, represent a snapshot of gene expression states. Specifically, $x^{(i)} \in \mathbb{R}^m$ represents the vector of m gene expression levels of cell i. We represent the sets of initial, intermediate and terminal states respectively with the ordered sets $\mathcal{X}_0 = (x^{(i)} \mid i = 1, \ldots, n_0)$, $\mathcal{X} = (x^{(i)} \mid i = n_0 + 1, \ldots, N - n_F)$ and $\mathcal{X}_F = (x^{(i)} \mid i = N - n_F + 1, \ldots, N)$,

In MultistageOT, cell differentiation is modeled as a multistage optimal mass transport problem, whereby initial cells in $\mathcal{X}_0$ are assumed to transition into terminal cells in $\mathcal{X}_F$ over T stages of differentiation in a way that minimizes a total transition cost. Due to the squared Euclidean distance cost, transitions corresponding to large jumps between cell states are associated with high cost. Thus, to achieve a minimum transition cost, intermediate cells in $\mathcal{X}$ must be recruited as feasible intermediate differentiation states (see *SI Appendix*, Supplementary Note for details). We here show how this induces a pseudotemporal ordering as well as cell fate probabilities of all intermediate cell states.

Pseudotime

Denote by $\mu_t^{(i)}$ the total mass transported through each intermediate state, $x^{(n_0+i)} \in \mathcal{X}$, in stage t for $i = 1, \ldots, N - n_0 - n_F$ and $t = 1, \ldots, T - 1$. By normalizing by the total transported mass (over all stages), we obtain, for each intermediate state, the corresponding probabilities

$$\mathbb{P}\left(x^{(n_0+i)} \in \mathcal{X} \text{ transports mass in stage } t\right) := \frac{\mu_t^{(i)}}{\sum_{k=1}^{T-1} \mu_k^{(i)}} \tag{1}$$

(illustrated in Fig. 4C). Similarly, let $\nu_t^{(i)}$ be the total mass received by each terminal cell state, $x^{(N-n_F+i)} \in \mathcal{X}_T$, in stage t for $i = 1, \ldots, n_F$ and $t = 1, \ldots, T - 1$. We obtain the probabilities

$$\mathbb{P}\left(x^{(N-n_F+i)} \in \mathcal{X}_F \text{ receives mass in stage } t\right) := \frac{\hat{\nu}_t^{(i)}}{\sum_{k=1}^{T-1} \hat{\nu}_k^{(i)}}. \tag{2}$$

Based on these probabilities, we compute, for each intermediate state and terminal state, an expected value over the different transport stages. A pseudotemporal ordering of the cells is then obtained by ranking the cells based on this expected transport stage, such that the cell with the earliest expected transport stage is assigned a pseudotime of 0, and the cell with the latest expected transport stage is assigned a pseudotime of 1. Initial states in $\mathcal{X}_0$ by definition have an expected transport stage of 0 and are thus all given a pseudotime of 0.

Cell fate probabilities

To quantify the likelihood of a cell ending up in a particular fate, we use results from Markov chain theory. In particular, we estimate cell fate probabilities by computing absorption probabilities in an absorbing Markov chain as follows, similar to Weinreb et al. [10] and Lange et al. [11].

Let $m_{ij}^{(t)}$ denote the amount of mass sent between cell i and any other cell j in the t:th stage, obtained by solving Algorithm 1 (see *SI Appendix*, Supplementary Note). To quantify the affinity between any two cell states, we aggregate the transport plans by summing the total mass sent between any two cells over all stages, i.e., we compute

$$m_{ij} = \sum_{t=1}^{T-1} m_{ij}^{(t)}, \quad \text{for } i = 1, \dots, N, \text{ and } j = 1, \dots, N. \tag{3}$$

For each initial and intermediate cell states (i.e., $i = 1, \dots, N - n_F$) the aggregated transport coupling is normalized to represent a probability of transition between cell i and cell $j = 1, \dots, N$:

$$a_{ij} := \frac{m_{ij}}{\sum_{k=1}^{N} m_{ik}} \quad \text{(cell } i \text{ to cell } j \text{ transition probability model).} \tag{4}$$

For terminal cell states, $i = N - n_F, \dots, N$, we have that $m_{ij} = 0$ for all $j = 1, \dots, N$, so we define

$$a_{ij} = \begin{cases} 1 & \text{if } i = j \\ 0, & \text{otherwise.} \end{cases} \tag{5}$$

We let the matrix $A = [a_{ij}]_{i,j=1}^{N}$ be a transition probability matrix in a stationary absorbing Markov chain, in which initial and intermediate cell states in $\mathcal{X}_0$ and $\mathcal{X}$ define the n transient states and the terminal cell states in $\mathcal{X}_F$ define the n_F absorbing states. We partition A according to

$$A = \begin{bmatrix} Q & R \\ \mathbf{0} & I_{n_F} \end{bmatrix}, \tag{6}$$

so that $Q = [q_{ij}]_{i,j=1}^{n}$ encodes transitions between transient states, R encodes transitions between transient and absorbing states, $\mathbf{0}$ is the $n_F \times n$ matrix of zeros, and I_{n_F} is the $n_F \times n_F$ identity matrix. It is a well-known result in Markov chain theory that the matrix

$$Z = (I_n - Q)^{-1} R, \tag{7}$$

with elements z_{ij}, $i = 1, \dots, n$, $j = 1, \dots, n_F$, encodes the probability of transient state i being absorbed in absorbing state j.

Assume K different classes of absorbing states (e.g., representing different lineages). The probability of transient state i ending up in a class k of absorbing states is obtained by summing the absorption probabilities over all states in class k. Hence, we compute the cell fate probability of cell i eventually being absorbed in fate k through

$$p_k^{(i)} := \sum_{j \in \mathcal{I}_k} z_{ij}, \tag{8}$$

where $\mathcal{I}_k = \{j \in \{1, \dots, n_F\} | \text{cell state } x^{(N-n_F+j)} \text{ belongs to fate } k\}$ denotes the index set corresponding to terminal states in fate class k. As a measure of potency of cell i, we

computed the Shannon entropy according to:

$$s^{(i)} = -\sum_{k=1}^{K} p_k^{(i)} \log p_k^{(i)}, \quad i = 1, \ldots, N. \tag{9}$$

Higher entropy $s^{(i)}$ reflects a lower degree of commitment, whereas lower values reflects a higher degree of commitment to a particular fate (the lowest value corresponds cell fate probabilities $p_k^{(i)}$ which is a singular distribution, supported only on a single fate, whereas the highest value corresponds to a uniform distribution over all fates).

Quantifying the model's predictive performance

Lineage tracing data from Weinreb et al. [2] was used to benchmark model predictions.

Predicting annotated mature cells: When comparing fate prediction with author annotations in Fig. 6F, we excluded cells from the terminal cell subset, $\mathcal{X}_F$, since these were used in fitting the models to the data. The dominant predicted fate probability (the fate predicted as most probable) in each cell was then compared to its annotation. The accuracy was taken as the ratio of correct matches to the total number of considered cells (a total of 51720 annotated cells were considered).

Predicting day 2 cells: When benchmarking the performance of predicting fates of day 2 cells, we chose day 2 cells satisfying each of the following criteria. In addition to being a cell from day 2, it needed to:

1. Belong to a clone with a non-empty set of clonal sisters in days 4 or 6.
2. Not be part of the set of predefined terminal states used in optimizing our model.
3. Have at least 7 clonal sisters in days 4 or 6.
4. Have at least 1 clonal sister in days 4 or 6 annotated as committed to any of the 10 identified cell lineages.
5. Not belong to a clone represented in more than one library type (which would indicate a spurious clonal relationship based on a repeated lineage barcode).

This left us with $N_{d_2} = 795$ day 2 cells. For each such cell, if it did not already have a cell type annotation (in which case it was assumed to be fully committed to that fate), we computed "ground truth" cell fate probabilities by counting the number of its clonal sisters in days 4 or 6 that were annotated as a particular mature cell type, ignoring the counts of clonal sisters named "Undifferentiated". The counts were then normalized to sum to one: inducing, for each day 2 cell, a probability distribution over the different mature cell types. More specifically, by letting $n_k^{(i)}$ denote the number of clonal sisters found in fate k at days 4 or 6 within the clone of a given day 2 cell i ($i = 1, \ldots, N_{d_2}$), the empirical cell fate probability, $q_k^{(i)}$, of cell i ending up in fate k was computed according to

$$q_k^{(i)} = \frac{n_k^{(i)}}{\sum_{k=1}^{K} n_k^{(i)}}. \tag{10}$$

Based on using these empirical cell fate probabilities as a ground truth, we could compute different performance metrics (see table in Fig. 6J) including total variation distance (TV), defined via

$$\mathrm{TV}(p, q) := \frac{1}{2} \sum_{k=1}^{K} |p_k - q_k|, \tag{11}$$

for two probably vectors, $p := (p_1, \ldots, p_K)$, $q := (q_1, \ldots, q_K)$, as well as dominant fate accuracy when predicting the most likely cell fate:

$$\text{dominant fate accuracy} := \frac{\text{correct classifications of most likely fate}}{N_{d_2}}. \tag{12}$$

We also developed two novel metrics to gauge cell fate predictive performance. The first, which we call total accuracy, is defined as the fraction of cells for which the m largest predicted fate probabilities matches the m observed clonal sister fates. More specifically, let $\mathcal{F}^{(i)}$ represent the set of observed clonal sister fates for cell i, i.e., $\mathcal{F}^{(i)} = \{k | q_k^{(i)} > 0\}$, and again let $p_k^{(i)}$ denote the predicted probability of fate k in cell i. Total accuracy is defined as the fraction of day 2 cells for which it holds that

$$\min \left\{ p_k^{(i)} \right\}_{k \in \mathcal{F}^{(i)}} > \max \left\{ p_k^{(i)} \right\}_{k \notin \mathcal{F}^{(i)}}. \tag{13}$$

In the second measure, presented in Fig. 6L, we turned the predicted (continuous) fate probabilities into categorical fate assignments by assigning to each cell any fate with a predicted probability above a threshold δ (Fig. 6K). This can be seen as a generalization of the dominant fate accuracy which corresponds to a single assignment (the dominant fate probability). We achieved a new type of performance metric by calculating the fraction of all cells with perfect matches after performing fate assignments at a particular threshold. Note that this performance metric depends on the threshold δ. To get a robust estimate for this generalized accuracy, we computed this value for all possible thresholds $\delta \in [0, 1]$ and computed an area under the curve (Fig. 6L).

StationaryOT

The StationaryOT [4] method is also based on optimal transport. It requires the user to specify sets of initial and terminal cell states (referred to as source and sink nodes respectively in Zhang et al. [4]). It represents a fundamentally different approach from MultistageOT in that StationaryOT solves a single-step transport problem to obtain couplings between cells, whereas MultistageOT solves a global transport problem over multiple transport steps (*SI Appendix*, Supplementary Note).

Following [4], we used the quadratic cost function $C(x, y) = \frac{1}{2}\|x - y\|_2^2$ between cell states x and y. In benchmarking predictive performance in Fig. 6, we applied StationaryOT for computing cell fates within each of the 12 partitions of the data set, using the same set of initial and terminal states as in MultistageOT, denoted $\mathcal{X}_0$ and $\mathcal{X}_F$ respectively. In addition to the entropy-regularization parameter, ϵ, StationaryOT requires the user to specify relative growth rates for the cells, $g_i = \exp(R_i)$, $i = 1, \ldots, N$ (N being the

number of cells and R_i being a "flux-rate" [4]) as well as a time step parameter Δt. In the absence of growth rate estimates in the data set, we used the following heuristic: we specified $g_i \equiv 0$ for any sink node $x^{(i)} \in \mathcal{X}_F$ and

$$R_i \equiv \frac{1}{\Delta t} \ln \left(\frac{n_F}{n_0} + 1 \right) \iff g_i \equiv \exp \left\{ \frac{1}{\Delta t} \ln \left(\frac{n_F}{n_0} + 1 \right) \right\} = \left(\frac{n_F}{n_0} + 1 \right)^{\frac{1}{\Delta t}}, \tag{14}$$

for any source node $x^{(i)} \in \mathcal{X}_0$, and $g_i = 1$ for any intermediate cell $x^{(i)} \in \mathcal{X}$. Note that this implies that

$$\sum_{i=1}^{N} g_i^{\Delta t} = n_F + n_0 + n = N, \tag{15}$$

so that, if we take the first marginal at $t = 0$ to be $\mu = g^{\Delta t}$ and we define the second marginal at $t = \Delta t$ as $\nu = \left(\frac{\sum_{i=1}^{N} \mu_i}{N} \right) \mathbf{1}_N$, we obtain $\nu = \mathbf{1}_N$.

The time step parameter was taken as $\Delta t = 0.25$, which was used by Zhang et al. [4] on the *Arabidopsis thaliana* root tip data set. The regularization parameter $\epsilon = 0.12$ was chosen small enough such that we did not experience underflow in the computation for the optimal couplings, and thus allowed us to obtain numerically stable results for the cell fate probabilities (both of these numerical issues are discussed in Zhang et al. [4]).

Inverse Distance Weighed (IDW) model

To gauge the performance of our modeling framework in relation to other approaches, we established a naive cell fate model, based only on the relative proximity to the different terminal fates and a prior fate bias. Let $\mathcal{X}_k \subset \mathcal{X}_F$ be the subset terminal states in $\mathcal{X}_F$ belonging to cell fate k and let $x^{(i)}$ denote the cell state of cell i in day 2. Specifically, the IDW model estimates the probability for a cell i to end up in fate k according to:

$$r_k^{(i)} = \frac{b_k \left(\frac{\|x^{(i)} - \bar{x}_k\|}{\|\bar{x}_0 - \bar{x}_k\|} \right)^{-\gamma}}{\sum_k b_k \left(\frac{\|x^{(i)} - \bar{x}_k\|}{\|\bar{x}_0 - \bar{x}_k\|} \right)^{-\gamma}}, \tag{16}$$

where b_k is a weight representing a prior fate bias for fate k such that $\sum_k b_k = 1$,

$$\bar{x}_0 = \frac{1}{|\mathcal{X}_0|} \sum_{x^{(i)} \in \mathcal{X}_0} x^{(i)} \tag{17}$$

is the sample mean vector of all initial states, and

$$\bar{x}_k = \frac{1}{|\mathcal{X}_k|} \sum_{x^{(i)} \in \mathcal{X}_k} x^{(i)} \tag{18}$$

is the sample mean of the terminal states in fate k. The parameter $\gamma \in [0, \infty)$ controls how much the distances to the fates should be weighted. As $\gamma \to \infty$, the cell fate probabilities

tend to the deterministic distributions corresponding to a single dominant fate, whereas $\gamma = 0$ corresponds to $r_k^{(i)} \equiv b_k$, for $i = 1, \ldots, N_{d_2}$. We base the prior weights, b_k, for each fate k, on the number of mature cell type annotations found in the corresponding fate (as annotated by Weinreb et al. [2]). We scaled these to sum to unity and obtained the weights shown in SI Appendix, Table S2. All distances were computed on the space spanned by the 50 first PCA components.

The IDW model depends on γ. We chose $\gamma \in [1, 50]$ so as to maximize the accuracy (12). This yielded the optimal $\gamma^* = 20$ (see *SI Appendix*, Fig. S3B).

CellRank2

We applied CellRank2 [12] (version 2.0.7) on the Weinreb et al. [2] data. We applied CellRank2's `ConnectivityKernel` and `PseudotimeKernel`, both designed for within-snapshot trajectory inference. The connectivity-kernel is based on an undirected nearest neighbor graph, whereas the pseudotime-kernel allows the inference of directed transition probabilities, by considering the directions of increasing pseudotime. We used their GPCCA estimator which allows the user to set initial and terminal states via the `set_initial_states` and `set_terminal_states` methods. To make the results comparable to MultistageOT, we used the same set of initial and terminal states as in MultistageOT. To estimate fate probabilities, we called CellRank2's estimator method `compute_fate_probabilities`.

StaVIA

We applied StaVIA (version 0.2.4) on the Weinreb et al. [2] data. StaVIA allows the user to specify true labels for each cell, e.g. corresponding to cell type annotations. We leveraged this to specify initial and terminal cell states. To make the results comparable to MultistageOT, all initial cells used as input to MultistageOT were given the label "root", and all terminal cells used as input to MultistageOT were given the corresponding "Cell type annotation" provided by the Weinreb et al. [2] data. We then ran StaVIA's `run_VIA` function, having specified the following parameters: `root_user = ['root']`, `dataset = ['group']`, and the `user_defined_terminal_group` parameter was set to a list of names of the 10 annotated blood cell lineages featured in the data. To estimate fate probabilities, we used StaVIA's `single_cell_bp_rownormed` attribute which gives probabilities towards each lineage.

Inference of bipotent basophil and mast cell progenitors

For the analysis of cells possessing both basophil and mast cell lineage-forming capacity (shown in Fig. 7J-K), we selected cells with MultistageOT-inferred fate probabilities above 10% for both the mast cell and basophil fates, and less than 1% for all other fates.

References

[1] Paul, F. *et al.* Transcriptional heterogeneity and lineage commitment in myeloid progenitors. *Cell* **163**, 1663–1677 (2015).

[2] Weinreb, C., Rodriguez-Fraticelli, A., Camargo, F. D. & Klein, A. M. Lineage tracing on transcriptional landscapes links state to fate during differentiation. *Science* **367**, eaaw3381 (2020).

[3] Dahlin, J. S. *et al.* A single-cell hematopoietic landscape resolves 8 lineage trajectories and defects in Kit mutant mice. *Blood* **131**, e1–e11 (2018).

[4] Zhang, S., Afanassiev, A., Greenstreet, L., Matsumoto, T. & Schiebinger, G. Optimal transport analysis reveals trajectories in steady-state systems. *PLoS Comput. Biol.* **17**, e1009466 (2021).

[5] Shahan, R. *et al.* A single-cell arabidopsis root atlas reveals developmental trajectories in wild-type and cell identity mutants. *Dev. Cell* **57**, 543–560 (2022).

[6] Zhang, K., Zemke, N. R., Armand, E. J. & Ren, B. A fast, scalable and versatile tool for analysis of single-cell omics data. *Nat. Methods* **21**, 217–227 (2024).

[7] Weng, C. *et al.* Deciphering cell states and genealogies of human haematopoiesis. *Nature* **627**, 389–398 (2024).

[8] Zhang, X. *et al.* An immunophenotype-coupled transcriptomic atlas of human hematopoietic progenitors. *Nat. Immunol.* **25**, 703–715 (2024).

[9] Pellin, D. *et al.* A comprehensive single cell transcriptional landscape of human hematopoietic progenitors. *Nat. Commun.* **10**, 2395 (2019).

[10] Weinreb, C., Wolock, S., Tusi, B. K., Socolovsky, M. & Klein, A. M. Fundamental limits on dynamic inference from single-cell snapshots. *Proc. Natl Acad. Sci. USA* **115**, E2467–E2476 (2018).

[11] Lange, M. *et al.* CellRank for directed single-cell fate mapping. *Nat. Methods* **19**, 159–170 (2022).

[12] Weiler, P., Lange, M., Klein, M., Pe'er, D. & Theis, F. Cellrank 2: unified fate mapping in multiview single-cell data. *Nat. Methods* **21**, 1196–1205 (2024).

Supplementary Note

Mathematical formulation of MultistageOT

Magnus Tronstad[1*], Johan Karlsson[2†*], and Joakim S. Dahlin[1†*]

[1]Department of Medicine Solna, Karolinska Institutet, and Center for Molecular Medicine, Karolinska University Hospital, Stockholm, Sweden.
[2]Department of Mathematics, KTH Royal Institute of Technology, Stockholm, Sweden.

[†]These authors jointly supervised the work.
[*]Corresponding authors. Emails: magnus.tronstad@ki.se, johan.karlsson@math.kth.se, and joakim.dahlin@ki.se

1 Notation

Whenever the exponential function is applied on vectors or matrices, it is assumed that it works component-wise. Also we use the following notation:

$\odot$	Component-wise multiplication (Hadamard product).
$\oslash$	Component-wise division.
$\langle \cdot, \cdot \rangle$	Frobenius inner product.
δ_x	Dirac delta point measure, corresponding to a unit mass located in x. Formally: $$\delta_x(A) = \begin{cases} 0, & x \notin A \\ 1, & x \in A, \end{cases}$$ for any measurable set A.
$(x_i)_{i=1}^n$	Ordered set of elements x_i, $i = 1, \dots, n$.
$\mathbb{R}_+$	The set $[0, \infty)$.
$\overline{\mathbb{R}}_+$	The nonnegative real line, including infinity $\mathbb{R}_+ \cup \{\infty\}$.
$\mathbf{1}_n$	A vector of all-ones of length n.

2 Background

Optimal transport solves the problem of moving one distribution into another in an optimal way [1]. This general framework can be traced back to the 18th century [2], and traditional areas of applications include economics and logistics [3, 4, 5]. Extension to a broader range of applications was for a long time hampered, as the computational cost associated with numerically computing an optimal transport solution placed severe limits on problem size. Notwithstanding, following a computational breakthrough due to Cuturi [6], it is

now possible to address and solve problems that were historically too large for standard numerical methods. This is accomplished by adding an entropic regularization term to the classical optimal transport formulation; Sinkhorn iterations [7] are then leveraged to efficiently obtain a near-optimal solution, even for very large problem sizes. Since then, the interest for optimal transport has increased rapidly and it has been applied to a wide range of problems, including image processing [8, 9], tracking and sensor fusion [10], ensemble control problems [11], fluid mechanics [12], the Schrödinger bridge problem [13] as well as flow problems [14, 15] and applications to biological systems [16, 17].

Recently, optimal transport theory has successfully been applied in the context of cellular development [18, 19, 20, 21, 22]. Many of these studies have been designed around time-series matching. In this setting, one is faced with multiple snapshots of single-cell sequencing data: Each snapshot comprises a large collection of individual measurements of cell states from a specific time point, and the goal is to match cells from consecutive time points in a biologically meaningful way. The cell states can be represented as point masses in state space, and a cost can be associated with the transportation of a unit mass between any pair of cells in two consecutive time points. Optimal transport then provides a natural mathematical framework for finding optimal (least costly) assignments of cell states between time points. In 2019, Schiebinger et al. [18] introduced Waddington-OT which uses optimal transport to find the optimal matching of single cells in pairs of consecutive time points. In Forrow et al. [20], this framework is combined with lineage information to improve the reconstruction of developmental trajectories. Yang et al. [19] extend the Waddington-OT approach by computing the transport costs in a latent space generated by an autoencoder.

Time-series data is not the only means by which one can study gene expression dynamics of cellular populations. When the developmental process originates in a population of continuously self-replenishing cell states (such as in blood cell development), a single sequencing snapshot can be expected to represent cells from a range of different stages of maturation, from blood stem cells to late stage progenitors. Thus, a second approach to inferring developmental trajectories relies on analyzing a single snapshot of single-cell sequencing data. Zhang et al. [21], presented StationaryOT, showing that classical bimarginal discrete optimal transport can be used for such within-snapshot trajectory inference when cell growth rate estimates are available.

In this work, we propose MultistageOT, a novel modeling framework for trajectory inference within a snapshot. MultistageOT extends bimarginal optimal transport so that transport occurs across multiple (more than two) marginals. This allows cell differentiation in a snapshot to be modeled as a transition process on gene expression over a number of transport steps, each step corresponding to an intermediate differentiation stage. This introduces a temporal axis to our model, which provides a natural way to order the cells in terms of maturity in the absence of time-resolved data.

This note contains information presented in the main article but expands on it for a complete mathematical exposition. In the remainder of this note, we briefly review some preliminaries on classical discrete optimal transport before presenting a mathematical formulation of our MultistageOT model of cell differentiation. We state the entropy-regularized optimization problem solved in MultistageOT, and then derive a generalized

Sinkhorn-Knopp algorithm for efficient computation of the optimal solution.

2.1 Optimal transport

MultistageOT is based on an extension of discrete optimal transport. In the classical discrete optimal transport setting, two discrete mass distributions are given:

$$\sum_{i=1}^{n_1} \mu_1^{(i)} \delta_{x_1^{(i)}}, \quad \sum_{j=1}^{n_2} \mu_2^{(j)} \delta_{x_2^{(j)}},$$

where $\mathcal{X}_1 = (x_1^{(i)})_{i=1}^{n_1}$ and $\mathcal{X}_2 = (x_2^{(j)})_{j=1}^{n_2}$ correspond to the points of support of the respective distributions, and where $\mu_1^{(i)}$ and $\mu_2^{(j)}$ represent the mass in the points $x_1^{(i)}$ and $x_2^{(j)}$, respectively. A transport plan is a matrix, $M = [m_{ij}]_{i=1,j=1}^{n_1,n_2}$, whose components m_{ij} denote the amount of mass transported from $x_1^{(i)} \in \mathcal{X}_1$ to $x_2^{(j)} \in \mathcal{X}_2$. We say that the transport plan is feasible if the total amounts transported are consistent with the initial and final distributions, i.e., if $M\mathbf{1}_{n_2} = \mu_1$ and $M^T\mathbf{1}_{n_1} = \mu_2$, where $\mu_1 = [\mu_1^{(i)}]_{i=1}^{n_1}$ and $\mu_2 = [\mu_2^{(j)}]_{j=1}^{n_2}$ are the vectors representing the mass distributions. Next, let $c_{ij} = c\left(x_1^{(i)}, x_2^{(j)}\right)$ denote the cost of moving a unit of mass from $x_1^{(i)}$ to $x_2^{(j)}$, and let $C = [c_{ij}]_{i=1,j=1}^{n_1,n_2}$ be the corresponding cost matrix. The optimal transport problem, then, is to find a feasible transport plan that moves the mass in μ_1 to μ_2 with minimal cost, i.e.,

$$\mathcal{T}(\mu_1, \mu_2) := \underset{M \in \mathbb{R}_+^{n_1 \times n_2}}{\text{minimize}} \quad \langle C, M \rangle \tag{2.1a}$$

$$\text{subject to} \quad M\mathbf{1}_{n_2} = \mu_1 \tag{2.1b}$$

$$M^T\mathbf{1}_{n_1} = \mu_2, \tag{2.1c}$$

where $\langle C, M \rangle = \sum_{i=1}^{n_1} \sum_{j=1}^{n_2} c_{ij} m_{ij}$. This problem is referred to as the Kantorovich formulation [1], and is a linear programming problem. For certain costs, this problem can be used to define a metric space. For example, when $c(x_1^{(i)}, x_2^{(j)}) = \|x_1^{(i)} - x_2^{(j)}\|^2$, the Wasserstein-2 metric between two distributions with equal mass is defined as the square root of the optimal transport cost (2.1).

For small enough problems, the formulation (2.1) can be solved using standard methods for linear programs such as the Simplex algorithm or interior-point methods. However, as the number of points in each support grows large, these methods become less practical; to efficiently address large scale problems, one can introduce an entropic regularization term in the objective as proposed by Cuturi [6]. The entropy-regularized optimal transport problem is then formulated as

$$\mathcal{T}_\epsilon(\mu_1, \mu_2) := \underset{M \in \mathbb{R}_+^{n_1 \times n_2}}{\text{minimize}} \quad \langle C, M \rangle + \epsilon D(M|P) \tag{2.2a}$$

$$\text{subject to} \quad M\mathbf{1}_{n_2} = \mu_1 \tag{2.2b}$$

$$M^T\mathbf{1}_{n_1} = \mu_2, \tag{2.2c}$$

where the scalar $\epsilon > 0$ is a parameter determining the weight given to the entropy term

$$D(M|P) = \sum_{i=1}^{n_1} \sum_{j=1}^{n_2} \left(m_{ij} \log \frac{m_{ij}}{p_{ij}} - m_{ij} + p_{ij} \right) \tag{2.3}$$

and where the matrix $P = [p_{ij}]_{i=1,j=1}^{n_1,n_2}$ can represent a prior distribution [23]. In general, P can be any matrix with strictly positive elements, but common choices include $P = \mathbf{1}_{n_1}\mathbf{1}_{n_2}^T$ [6] or $P = \mu_1\mu_2^T$. It can be shown [6] that the solution to (2.2) is in the form

$$M_\epsilon^* = K \odot (u_1 u_2^T) \tag{2.4}$$

where $K = \exp(-C/\epsilon) \odot P$, and the vectors $u_1 \in \mathbb{R}^{n_1}$, $u_2 \in \mathbb{R}^{n_2}$ can be obtained through Sinkhorn iterations:

$$u_1 \leftarrow \mu_1 \oslash (K u_2), \quad u_2 \leftarrow \mu_2 \oslash (K^T u_1). \tag{2.5}$$

Note that these iterations can be performed efficiently since the bottleneck is matrix-vector multiplication. It can also be shown that if the costs are finite the algorithm converges linearly [24]. There are several ways to interpret Sinkhorn's method: scaling via diagonal matrix multiplication [7], iterative Bregman projections [25], Dykstra's algorithm [25], or dual coordinate ascent [26].

3 Multistage optimal transport

We model a snapshot of single-cell RNA sequencing (scRNA-seq) data as a collection of data points generated by multiple trajectories from a continuous time-invariant (unknown) dynamical system with state variables, $x_k(t) \in \mathbb{R}$, $k = 1, \ldots, m$, corresponding to the gene expression level of gene k at time $t \in \mathbb{R}_+$ (note that t does not correspond to elapsed real time; rather, it measures progression through the differentiation process). Under this framework, the transcriptional state of a cell is represented by the state vector $x(t) = (x_1(t), x_2(t), \ldots, x_m(t)) \in \mathbb{R}^m$ which, as it develops, traces a trajectory in m dimensional gene expression space (one dimension per gene).

Now assume we are given a snapshot $\mathcal{D} = \{x^{(i)} \,|\, i = 1, \ldots, N\}$, corresponding to a collection of cell states, $x^{(i)} = (x_1^{(i)}, x_2^{(i)}, \ldots, x_m^{(i)}) \in \mathbb{R}^m$, representing the measured RNA expression levels of m different genes, for each cell $i = 1, \ldots, N$. Each cell state is assumed to have been generated by sampling a state trajectory from the underlying dynamical system after some time t. We thus assume that $\mathcal{D}$ contains cells from a range of intermediate stages of differentiation. Let the first n_0 cells in $\mathcal{D}$ correspond to the most immature cell states; we refer to these as the initial states, and denote them by $\mathcal{X}_0$. Let the last n_F cells correspond to the most mature cell states; we refer to these as the terminal states, and denote them by $\mathcal{X}_F$. The remaining cells are referred to as intermediate states and are denoted $\mathcal{X}$ (see Fig. 2 in main article). These three ordered sets are thus given by $\mathcal{X}_0 = (x^{(i)} \,|\, i = 1, \ldots, n_0)$, $\mathcal{X}_F = (x^{(i)} \,|\, i = N - n_F + 1, \ldots, N)$, and $\mathcal{X} = (x^{(i)} \,|\, i = n_0 + 1, \ldots, N - n_F)$. We assume that $\mathcal{X}_0$ and $\mathcal{X}_F$ are known, and one main problem that needs to be solved is to determine how the cells in $\mathcal{X}$ should be ordered in terms of temporal

progression through the differentiation process. Note that this means that we do not here consider the problem of discovering $\mathcal{X}_0$ and $\mathcal{X}_F$, and our approach is thus limited in systems where such states are less well-characterized or unknown. However, the assumption of known initial and terminal cells holds in many biological systems, such as hematopoiesis, where there are well-established gene markers for multipotent and committed progenitors respectively that can be used to define the initial and terminal states. In such systems, the fundamental problem remains of characterizing the intermediate cell states, which is the focus of our work.

With MultistageOT, we model the cell differentiation process as a process on gene expression space, in which the cells corresponding to the initial states in $\mathcal{X}_0$ are assumed to transition into the terminal states in $\mathcal{X}_F$ over at most T discrete time steps, using all the intermediate states as possible transit hubs. With each state transition we associate a cost, modeling the likelihood of the transition, and we seek the transitions that minimize the overall cost. As we will see, this can be formulated using optimal mass transport.

To that end, we equip each cell with a notion of "mass", that can be transported to other cells, and define the cost[1] of transporting a unit of mass between any two cells with states $x^{(i)}, x^{(j)} \in \mathbb{R}^m$ as

$$c(x^{(i)}, x^{(j)}) = \left\|x^{(i)} - x^{(j)}\right\|_2^2, \tag{3.1}$$

i.e., the squared Euclidean distance between $x^{(i)}$ and $x^{(j)}$. Moreover, to allow for more concise notation, with two ordered sets, $\mathcal{X}_1 = (x_1^{(i)} \,|\, i = 1, \dots, n_1)$ and $\mathcal{X}_2 = (x_2^{(j)} \,|\, j = 1, \dots, n_2)$, we associate a cost matrix $c(\mathcal{X}_1, \mathcal{X}_2) \in \overline{\mathbb{R}}_+^{n_1 \times n_2}$, with elements

$$c(\mathcal{X}_1, \mathcal{X}_2) = \left[c(x_1^{(i)}, x_2^{(j)})\right]_{i=1,j=1}^{n_1,n_2} = \left[\left\|x^{(i)} - x^{(j)}\right\|_2^2\right]_{i=1,j=1}^{n_1,n_2}. \tag{3.2}$$

To reflect biology, we assume that the mass transportation starts in the initial states, $\mathcal{X}_0$, and terminates in the terminal states, $\mathcal{X}_F$. In each time step, cells can transport mass to other cells in either $\mathcal{X}$ or $\mathcal{X}_F$. Any mass transported to $\mathcal{X}$ remains within the system and can be further transported in subsequent time steps, whereas mass sent to $\mathcal{X}_F$ exits the system—representing a terminal differentiation step. Let $\tilde{\mu}_0^{(i)}$, for $i = 1, \dots, n_0$, denote the portion of mass that is transported in $t = 0$ from cell $x^{(i)} \in \mathcal{X}_0$ to cells in $\mathcal{X}$, and let $\hat{\mu}_t^{(i)}$ denote the corresponding portion that is transported to $\mathcal{X}_F$. For notational brevity, let these two mass distributions be represented by the vectors

$$\tilde{\mu}_0 = \left[\tilde{\mu}_0^{(i)}\right]_{i=1}^{n_0}, \quad \text{(mass to } \mathcal{X}\text{, } \textbf{remains} \text{ in system)} \tag{3.3}$$

$$\hat{\mu}_0 = \left[\hat{\mu}_0^{(i)}\right]_{i=1}^{n_0} \quad \text{(mass to } \mathcal{X}_F\text{, } \textbf{exits} \text{ system).} \tag{3.4}$$

Similarly, in subsequent time steps $t = 1, \dots, T-1$, cells in $\mathcal{X}$ must transport mass received in the previous time step, $t-1$, to cells either in $\mathcal{X}$ or $\mathcal{X}_F$. As before, whatever mass is sent to $\mathcal{X}_F$ will exit the system. Thus, for $i = 1, \dots, N - n_0 - n_F$, we let $\tilde{\mu}_t^{(i)}$ denote the

[1] While the norm used in the cost in (3.1) is the squared Euclidean norm, it should be noted that this framework in principle can be used with any type of norm or generalized notion of a transport cost between states.

portion of mass transported in time step t from cell $x^{(n_0+i)} \in \mathcal{X}$ to cells in $\mathcal{X}$ and let $\hat{\mu}_t^{(i)}$ denote the corresponding portion transported to $\mathcal{X}_F$. We define, for each $t = 1, \ldots, T-1$, the corresponding vectors

$$\tilde{\mu}_t = \left[\tilde{\mu}_t^{(i)}\right]_{i=1}^{N-n_0-n_F}, \quad \text{(mass to } \mathcal{X}, \textbf{ remains} \text{ in system)} \tag{3.5}$$

$$\hat{\mu}_t = \left[\hat{\mu}_t^{(i)}\right]_{i=1}^{N-n_0-n_F} \quad \text{(mass to } \mathcal{X}_F, \textbf{ exits} \text{ system).} \tag{3.6}$$

Finally, let $\tilde{\nu}_t = [\tilde{\nu}_t^{(i)}]_{i=1,\ldots,N-n_0-n_F}$, for $t = 0, 1, \ldots, T-1$, be the vector whose element $\tilde{\nu}_t^{(i)}$ denotes the mass received by intermediate states $x^{(n_0+i)} \in \mathcal{X}$ ($i = 1, \ldots, N - n_0 - n_F$) in time step t, and let $\hat{\nu}_t = [\hat{\nu}_t^{(i)}]_{i=1,\ldots,n_F}$, for $t = 0, 1, \ldots, T-1$, be the vector whose element $\hat{\nu}_t^{(i)}$ denotes the mass received by terminal state x^{N-n_F+i} ($i = 1, \ldots, n_F$) in time step t. A schematic overview of this sequential mass transport process is shown in Fig. 3 in main article.

In this work, we take the global minimum cost transport plans—describing how mass should most efficiently be sent through all the cell states, over all time points—as a model for possible differentiation trajectories. To achieve this, we constrain the transport plans in the following way:

1. The total mass transported from a cell in time step t must be equal to that which was received by that same cell in the previous time step, i.e.,

$$\tilde{\mu}_t + \hat{\mu}_t = \tilde{\nu}_{t-1}, \quad \text{for } t = 0, 1, \ldots, T-2, \tag{3.7}$$

$$\hat{\mu}_{T-1} = \tilde{\nu}_{T-2}. \tag{3.8}$$

   Note that in the last step of transport, the intermediate states can only transport mass to cells in $\mathcal{X}_F$.

2. Every initial state should transport at least a unit amount of mass, i.e., we impose that

$$\tilde{\mu}_0 + \hat{\mu}_0 \geq \mathbf{1}_{n_0}. \tag{3.9}$$

3. Every intermediate state should in total, over all time steps $t = 1, \ldots, T-1$, transport at least a unit amount of mass. We formulate this as

$$\sum_{t=1}^{T-2} \tilde{\mu}_t + \sum_{t=1}^{T-1} \hat{\mu}_t \geq \mathbf{1}_{N-n_0-n_F}. \tag{3.10}$$

4. Every terminal state should, over all time steps $t = 1, \ldots, T-1$, receive at least a unit amount of mass, i.e., we impose that

$$\sum_{t=0}^{T-1} \hat{\nu}_t \geq \mathbf{1}_{n_F}. \tag{3.11}$$

Now, let

$$\tilde{M}_0 = \Big[(\tilde{M}_0)_{ij}\Big]_{i=1,j=1}^{n_0,N-n_0-n_F} \tag{3.12}$$

be the transport plan matrix whose components $(\tilde{M}_0)_{ij}$, for $i = 1, \ldots, n_0$, $j = 1, \ldots, N - n_0 - n_F$, denote the mass sent from $x^{(i)} \in \mathcal{X}_0$ to $x^{(n_0+j)} \in \mathcal{X}$ in time step $t = 0$, and let

$$\hat{M}_0 = \Big[(\hat{M}_0)_{ij}\Big]_{i=1,j=1}^{n_0,n_F} \tag{3.13}$$

be the transport plan matrix whose components $(\hat{M}_0)_{ij}$, for $i = 1, \ldots, n_0$, $j = 1, \ldots, n_F$, denote the amount of mass sent from $x^{(i)} \in \mathcal{X}_0$ to $x^{(N-n_F+j)} \in \mathcal{X}_F$ in time step $t = 0$. Note that the following relations hold

$$\tilde{\mu}_0 = \tilde{M}_0 \mathbf{1}_{n_0}, \quad \hat{\mu}_0 = \hat{M}_0 \mathbf{1}_{n_F} \tag{3.14}$$

Analogously, we define the transport matrices

$$\tilde{M}_t = \Big[(\tilde{M}_t)_{ij}\Big]_{i=1,j=1}^{N-n_0-n_F,N-n_0-n_F}, \quad \text{for } t = 1, \ldots, T-2 \tag{3.15}$$

$$\hat{M}_t = \Big[(\hat{M}_t)_{ij}\Big]_{i=1,j=1}^{N-n_0-n_F,n_F} \quad \text{for } t = 1, \ldots, T-1 \tag{3.16}$$

such that

$$\tilde{\mu}_t = \tilde{M}_t \mathbf{1}_{N-n_0-n_F}, \quad \text{for } t = 1, \ldots, T-2 \tag{3.17}$$

$$\hat{\mu}_t = \hat{M}_t \mathbf{1}_{n_F} \quad \text{for } t = 1, \ldots, T-1. \tag{3.18}$$

For each transport plan matrix, for $t = 0, 1, \ldots, T-1$, we associate corresponding cost matrices $\tilde{C}_t, \hat{C}_t$ of matching sizes (i.e., $\tilde{C}_t$ corresponds to $\tilde{M}_t$ and $\hat{C}_t$ to $\hat{M}_t$), so that $(\tilde{C}_t)_{ij}$ and $(\hat{C}_t)_{ij}$ encodes the cost associated with mass transports $(\tilde{M}_t)_{ij}$ and $(\hat{M}_t)_{ij}$ respectively.

3.1 Optimization problem

Recall that, in (2.1), $\mathcal{T}(\mu_1, \mu_2)$ is defined as the optimal objective function value in the optimal mass transport problem of moving μ_1 to μ_2 in a minimum cost way. Using this formalism, the multistage optimal mass transport problem over all time steps can be formulated as

$$(P) \quad \begin{aligned} \underset{\substack{\tilde{\mu}_t, \hat{\mu}_t, \hat{\nu}_t \\ t=0,\ldots,T-1}}{\text{minimize}} \quad & \sum_{t=0}^{T-2} \tilde{\mathcal{T}}_t(\tilde{\mu}_t, \tilde{\mu}_{t+1} + \hat{\mu}_{t+1}) + \sum_{t=0}^{T-1} \hat{\mathcal{T}}_t(\hat{\mu}_t, \hat{\nu}_t) \\ \text{subject to} \quad & \tilde{\mu}_0 + \hat{\mu}_0 \geq \mathbf{1}_{n_0} \\ & \sum_{t=1}^{T-2} \tilde{\mu}_t + \sum_{t=1}^{T-1} \hat{\mu}_t \geq \mathbf{1}_{N-n_0-n_F} \\ & \sum_{t=0}^{T-1} \hat{\nu}_t \geq \mathbf{1}_{n_F}. \end{aligned}$$

A more explicit formulation can be obtained by expressing (P) directly in terms of the transport plan matrices $\tilde{M}_t$ for $t = 0, 1, \ldots, T-2$ and $\hat{M}_t$ for $t = 0, 1, \ldots, T-1$. For brevity, we define $n = N - n_0 - n_F$, and let $n_t = n$ for $t = 1, \ldots, T-1$ Then,

$$\begin{aligned}
\underset{\substack{\tilde{M}_t, \hat{M}_t \geq 0 \\ t=0,\ldots,T-1}}{\text{minimize}} \quad & \sum_{t=0}^{T-2} \langle \tilde{C}_t, \tilde{M}_t \rangle + \sum_{t=0}^{T-1} \langle \hat{C}_t, \hat{M}_t \rangle && (3.19a) \\
\text{subject to} \quad & \tilde{M}_0 \mathbf{1}_{n_1} + \hat{M}_0 \mathbf{1}_{n_F} \geq \mathbf{1}_{n_0} && (3.19b) \\
& \tilde{M}_t \mathbf{1}_{n_{t+1}} + \hat{M}_t \mathbf{1}_{n_F} = \tilde{M}_{t-1}^T \mathbf{1}_{n_{t-1}} \quad \text{for } t = 1, \ldots, T-2 && (3.19c) \\
& \hat{M}_{T-1} \mathbf{1}_{n_F} = \tilde{M}_{T-2}^T \mathbf{1}_{n_{T-2}} && (3.19d) \\
& \sum_{t=1}^{T-2} \tilde{M}_t \mathbf{1}_{n_{t+1}} + \sum_{t=1}^{T-1} \hat{M}_t \mathbf{1}_{n_F} \geq \mathbf{1}_n && (3.19e) \\
& \sum_{t=0}^{T-1} \hat{M}_t^T \mathbf{1}_{n_t} \geq \mathbf{1}_{n_F}. && (3.19f)
\end{aligned}$$

Note that (3.19) is a linear program. However, due to the sizes of modern scRNA-seq data sets, it is not practical to solve the problem (3.19) with standard methods for linear programs. Moreover, due to the large amount of noise inherent in the data, the transport maps in the solution to (3.19), lying on an edge of the feasible polytope, are likely overly sparse (i.e., mapping one cell to few other cells). To resolve these issues, we instead solve an approximate optimal transport problem in which a regularizing entropy term is added to the objective function (see subsection 2.1 for details). This regularization not only allows us to derive an efficient iterative algorithm for solving the problem numerically, but it also promotes more diffuse transport maps, reducing the variance in the estimated cell-cell couplings. The regularized problem solved in MultistageOT is

$$\begin{aligned}
\underset{\substack{\tilde{M}_t, \hat{M}_t \\ t=0,\ldots,T-1}}{\text{minimize}} \quad & \sum_{t=0}^{T-2} \Big(\langle \tilde{C}_t, \tilde{M}_t \rangle + \epsilon D(\tilde{M}_t | \tilde{P}_t) \Big) + \sum_{t=0}^{T-1} \Big(\langle \hat{C}_t, \hat{M}_t \rangle + \epsilon D(\hat{M}_t | \hat{P}_t) \Big) && (3.20a) \\
\text{subject to} \quad & \tilde{M}_0 \mathbf{1}_{n_1} + \hat{M}_0 \mathbf{1}_{n_F} \geq \mathbf{1}_{n_0} && (3.20b) \\
& \tilde{M}_t \mathbf{1}_{n_{t+1}} + \hat{M}_t \mathbf{1}_{n_F} = \tilde{M}_{t-1}^T \mathbf{1}_{n_{t-1}}, \quad \text{for } t = 1, \ldots, T-2 && (3.20c) \\
& \hat{M}_{T-1} \mathbf{1}_{n_F} = \tilde{M}_{T-2}^T \mathbf{1}_{n_{T-2}}, && (3.20d) \\
& \sum_{t=1}^{T-2} \tilde{M}_t \mathbf{1}_{n_{t+1}} + \sum_{t=1}^{T-1} \hat{M}_t \mathbf{1}_{n_F} \geq \mathbf{1}_n && (3.20e) \\
& \sum_{t=0}^{T-1} \hat{M}_t^T \mathbf{1}_{n_t} \geq \mathbf{1}_{n_F}, && (3.20f)
\end{aligned}$$

where $\tilde{P}_t$ and $\hat{P}_t$ are "prior" transport plan matrices corresponding to $\tilde{M}_t$ and $\hat{M}_t$ respectively (see 2.1). Intuitively, the entropy-regularization penalizes deviations from these

prior matrices. In case $\tilde{P}_t$ and $\hat{P}_t$ are simply taken to be matrices of ones, it will favor less sparse optimal transport plans in general. Thus, the solution depends on the choice of the regularization parameter, ϵ (smaller values of ϵ promote sparser transport plans, whereas larger values promote denser transport plans). In Fig. S15, MultistageOT is applied to a 2D toy data set to visualize the optimal solution to (3.20).

The marginals $\tilde{\mu}_t$, for $t = 0, 1 \ldots, T-2$, and $\hat{\mu}_t$, for $t = 0, 1 \ldots, T-1$, are retrieved from the optimal transport plans via (3.14), (3.17) and (3.18). The total mass sent from every intermediate state is given by

$$\mu_t := \tilde{\mu}_t + \hat{\mu}_t \quad \text{for } t = 1, \ldots, T-2, \tag{3.21}$$

and $\mu_{T-1} := \hat{\mu}_{T-1}$. These intermediate marginals can be scaled so that they represent, for each intermediate state, the probability of that state belonging to a particular transport stage t. This allows us to compute a mean transport stage for each state and thereby order cells in terms of temporal progression through the differentiation process (see Fig. 4C-E, and Supplementary Methods for more details on downstream analyses based on the optimal transport solution).

4 Algorithm

The iterative algorithm for solving the entropy-regularized MultistageOT problem (3.20) presented in this work can be viewed as a form of generalized Sinkhorn-Knopp [7] iterations, and we here derive it using Lagrangian duality. The iterations can be interpreted as performing block-coordinate ascent on the dual to (3.20).

4.1 Duality

To allow for more succinct notation, we let

$$\mathbf{M} = (\tilde{M}_0, \hat{M}_0, \tilde{M}_1, \hat{M}_1 \ldots, \tilde{M}_{T-2}, \hat{M}_{T-2}, \hat{M}_{T-1}) \tag{4.1}$$

denote the tuple of all transport plans, and we let $\mathbf{\Lambda} = (\rho, \lambda_0, \lambda_1, \ldots, \lambda_T)$ denote the tuple of all the Lagrange multipliers (i.e., the primal and dual variables respectively).

The Lagrange function corresponding to (3.20) is then

$$\begin{aligned}
\mathcal{L}(\mathbf{M}, \mathbf{\Lambda}) = &\sum_{t=0}^{T-2} \left(\langle \tilde{C}_t, \tilde{M}_t \rangle + \epsilon D(\tilde{M}_t | \tilde{P}_t) \right) + \sum_{t=0}^{T-1} \left(\langle \hat{C}_t, \hat{M}_t \rangle + \epsilon D(\hat{M}_t | \hat{P}_t) \right) \\
&+ \rho^T \left(\mathbf{1}_n - \left(\sum_{t=1}^{T-2} \tilde{M}_t \mathbf{1}_{n_{t+1}} + \sum_{t=1}^{T-1} \hat{M}_t \mathbf{1}_{n_F} \right) \right) + \lambda_0^T \left(\mathbf{1}_{n_0} - \tilde{M}_0 \mathbf{1}_{n_1} - \hat{M}_0 \mathbf{1}_{n_F} \right) \\
&+ \sum_{t=1}^{T-2} \lambda_t^T \left(\tilde{M}_{t-1}^T \mathbf{1}_{n_{t-1}} - \tilde{M}_t \mathbf{1}_{n_{t+1}} - \hat{M}_t \mathbf{1}_{n_F} \right) \\
&+ \lambda_{T-1}^T \left(\tilde{M}_{T-2}^T \mathbf{1}_{n_{T-1}} - \hat{M}_{T-1} \mathbf{1}_{n_F} \right) + \lambda_T^T \left(\left(\sum_{t=0}^{T-1} \hat{M}_t^T \mathbf{1}_{n_t} \right) - \mathbf{1}_{n_F} \right)
\end{aligned}$$

and the Lagrange dual problem is

$$\max_{\mathbf{\Lambda}|\rho,\lambda_0,\lambda_T\geq 0} \inf_{\mathbf{M}\geq 0} \mathcal{L}(\mathbf{M},\mathbf{\Lambda}). \tag{4.2}$$

The key to deriving the algorithm presented in this work lies in leveraging the block-like structure in the problem induced by the different transport stages. In the following proposition, each transport plan is expressed in terms of the dual variables, and the dual problem is expressed on a form which emphasizes this block-structure.

Proposition 4.1. *Assume there exists a feasible solution to* (3.20) *with* $(\tilde{M}_t)_{ij} > 0$ *whenever* $(\tilde{C}_t)_{ij} < \infty$ *and* $(\hat{M}_t)_{ij} > 0$ *whenever* $(\hat{C}_t)_{ij} < \infty$*. Then, the optimal transport plans in* (3.20) *may be factorized as*

$$\boxed{\begin{aligned} \tilde{M}_t &= \tilde{K}_t \odot \tilde{U}_t, \quad \text{for } t = 0,1,\dots,T-2,\\ \hat{M}_t &= \hat{K}_t \odot \hat{U}_t, \quad \text{for } t = 0,1,\dots,T-1, \end{aligned}}$$

where

$$\begin{aligned} \tilde{K}_t &= \tilde{P}_t \odot \exp(-\tilde{C}_t/\epsilon), \quad \text{for } t = 0,1,\dots,T-2,\\ \hat{K}_t &= \hat{P}_t \odot \exp(-\hat{C}_t/\epsilon), \quad \text{for } t = 0,1,\dots,T-1,\\ \tilde{U}_t &= (u_t \odot s_t)v_{t+1}^T, \quad \text{for } t = 0,1,\dots,T-2,\\ \hat{U}_t &= (u_t \odot s_t)v_T^T, \quad \text{for } t = 0,1,\dots,T-1, \end{aligned}$$

and where

$$\begin{aligned} u_t &= \exp(\lambda_t/\epsilon) \quad && t = 0,1\dots,T\\ v_t &= \exp(-\lambda_t/\epsilon) \quad && t = 0,1,\dots,T\\ s_t &= \begin{cases} \mathbf{1}_{n_0} & t = 0\\ \exp(\rho/\epsilon) & t = 1,\dots,T-1. \end{cases} \end{aligned}$$

Moreover, strong-duality holds, and the dual function of (3.20) *may, up to a constant, be expressed as*

$$\begin{aligned} \varphi(\mathbf{\Lambda}) = &-\epsilon\big(e^{\lambda_0/\epsilon}\big)^T\left(\tilde{K}_0 e^{-\lambda_1/\epsilon} + \hat{K}_0 e^{-\lambda_T/\epsilon}\right)\\ &-\epsilon\sum_{t=1}^{T-2}\big(e^{\lambda_t/\epsilon}\odot e^{\rho/\epsilon}\big)^T\big(\tilde{K}_t e^{-\lambda_{t+1}/\epsilon} + \hat{K}_t e^{-\lambda_T/\epsilon}\big)\\ &-\epsilon\big(e^{\lambda_{T-1}/\epsilon}\odot e^{\rho/\epsilon}\big)^T\left(\hat{K}_{T-1}e^{-\lambda_T/\epsilon}\right)\\ &+\mathbf{1}_{n_0}^T\lambda_0 + \mathbf{1}_{n_F}^T\lambda_T + \mathbf{1}_n^T\rho. \end{aligned}$$

Proof. Assume first that $(\tilde{C}_t)_{ij} < \infty$, $(\hat{C}_t)_{ij} < \infty$ for all i,j and t, in which case there always exists a strictly positive feasible solution to (3.20) (e.g., sending mass uniformly

to all other cells in each time point). Since the objective (3.20a) is strictly convex, if a minimizer $\mathbf{M}^*$ exists, it is unique. Moreover, since the entropy-regularization terms have infinite, negative, slope at $(\tilde{M}_t)_{ij} = 0$, $(\hat{M}_t)_{ij} = 0$, for $i = 1, \ldots, n_t$, $j = 1, \ldots, n_{t+1}$, and $t = 0, \ldots, T-1$, it follows that all elements in the optimal solution will be strictly positive. Therefore, since the Lagrange function is continuously differentiable with respect to $\mathbf{M}$, we know that any minimizer $\mathbf{M}^*$ must satisfy

$$\nabla_{\mathbf{M}} \mathcal{L}(\mathbf{M}^*) = 0.$$

Differentiating $\mathcal{L}$ with respect to the variables $(\tilde{M}_0)_{ij}$ for $i = 1, \ldots, n_0, j = 1, \ldots, n_1$ and setting the derivative to zero yields

$$(\tilde{M}_0)_{ij} = (\tilde{P}_0)_{ij} \exp\Big(-(\tilde{C}_0)_{ij}/\epsilon + (\lambda_0)_i/\epsilon - (\lambda_1)_j/\epsilon\Big),$$

or, equivalently,

$$\begin{aligned}\tilde{M}_0 &= \mathrm{diag}(u_0)\tilde{P}_0 \odot \exp(-\tilde{C}_0/\epsilon)\mathrm{diag}(v_1) = \tilde{P}_0 \odot \exp(-\tilde{C}_0/\epsilon) \odot \left(u_0 v_1^T\right) = \tilde{K}_0 \odot \left(u_0 v_1^T\right) \\ &= \{s_0 = \mathbf{1}_{n_0}\} = \tilde{K}_0 \odot \left((u_0 \odot s_0) v_1^T\right).\end{aligned}$$

Doing the same for $(\hat{M}_0)_{ij}$, for $i = 1, \ldots, n_0$, $j = 1, \ldots, n_F$, yields

$$(\hat{M}_0)_{ij} = (\hat{P}_0)_{ij} \exp\Big(-(\hat{C}_0)_{ij}/\epsilon + (\lambda_0)_i/\epsilon - (\lambda_T)_j/\epsilon\Big),$$

or,

$$\hat{M}_0 = \hat{K}_0 \left(u_0 v_T^T\right) = \{s_0 = \mathbf{1}_{n_0}\} = \hat{K}_0 \left((u_0 \odot s_0) v_T^T\right).$$

Similarly, for $t = 1, \ldots, T-2$ we obtain

$$(\tilde{M}_t)_{ij} = (\tilde{P}_t)_{ij} \exp\Big(-(\tilde{C}_t)_{ij}/\epsilon + \rho_i/\epsilon + (\lambda_t)_i/\epsilon - (\lambda_{t+1})_j/\epsilon\Big),$$

for $i = 1, \ldots, n, j = 1, \ldots, n$, and

$$(\hat{M}_t)_{ij} = (\hat{P}_t)_{ij} \exp\Big(-(\hat{C}_t)_{ij}/\epsilon + \rho_i/\epsilon + (\lambda_t)_i/\epsilon - (\lambda_T)_j/\epsilon\Big),$$

for $i = 1, \ldots, n, j = 1, \ldots, n_F$. Hence, for $t = 1, \ldots, T-2$, we have that

$$\begin{aligned}\tilde{M}_t &= \tilde{K}_t \odot \left((u_t \odot s_t) v_{t+1}^T\right), \\ \hat{M}_t &= \hat{K}_t \odot \left((u_t \odot s_t) v_T^T\right).\end{aligned}$$

Finally, for $t = T-1$, we obtain

$$(\hat{M}_{T-1})_{ij} = (\hat{P}_{T-1})_{ij} \exp\Big(-(\hat{C}_{T-1})_{ij}/\epsilon + \rho_i/\epsilon + (\lambda_{T-1})_i/\epsilon - (\lambda_T)_i/\epsilon\Big),$$

for $i = 1, \ldots, n, j = 1, \ldots, n_F$, which implies

$$\hat{M}_{T-1} = \hat{K}_{T-1} \odot \left((u_{T-1} \odot s_{T-1}) v_T^T\right).$$

It follows that in the optimal solution, the entropy-regularization for $\tilde{M}_0$ reduces to

$$\begin{aligned}
\epsilon D(\tilde{M}_0|\tilde{P}_0) =& \epsilon \sum_{i=1}^{n_0}\sum_{j=1}^{n}(\tilde{M}_0)_{ij}\log(\tilde{M}_0)_{ij} - (\tilde{M}_0)_{ij}\log(\tilde{P}_0)_{ij} - (\tilde{M}_0)_{ij} + (\tilde{P}_0)_{ij} \\
=& -\sum_{i=1}^{n_0}\sum_{j=1}^{n}(\tilde{M}_0)_{ij}(\tilde{C}_0)_{ij} + \sum_{i=1}^{n_0}\sum_{j=1}^{n}(\tilde{M}_0)_{ij}(\lambda_0)_i - \sum_{i=1}^{n_0}\sum_{j=1}^{n}(\tilde{M}_0)_{ij}(\lambda_1)_j \\
& - \epsilon\sum_{i=1}^{n_0}\sum_{j=1}^{n}(\tilde{M}_0)_{ij} + \epsilon\sum_{i=1}^{n_0}\sum_{j=1}^{n}(\tilde{P}_0)_{ij} \\
=& -\langle \tilde{C}_0, \tilde{M}_0\rangle + \lambda_0^T\tilde{M}_0\mathbf{1}_{n_1} - \lambda_1^T\tilde{M}_0^T\mathbf{1}_{n_0} - \epsilon\mathbf{1}_{n_0}^T\tilde{M}_0\mathbf{1}_{n_1} + \epsilon\mathbf{1}_{n_0}^T\tilde{P}_0\mathbf{1}_{n_1}.
\end{aligned}$$

Similarly, we get that

$$\epsilon D(\hat{M}_0|\hat{P}_0) = -\langle \hat{C}_0, \hat{M}_0\rangle + \lambda_0^T\hat{M}_0\mathbf{1}_{n_F} - \lambda_1^T\hat{M}_0^T\mathbf{1}_{n_0} - \epsilon\mathbf{1}_{n_0}^T\hat{M}_0\mathbf{1}_{n_F} + \epsilon\mathbf{1}_{n_0}^T\hat{P}_0\mathbf{1}_{n_F}$$

For $t = 1, \dots, T-2$, we have

$$\begin{aligned}
\epsilon D(\tilde{M}_t|\tilde{P}_t) =& -\langle \tilde{C}_t, \tilde{M}_t\rangle + \rho^T\tilde{M}_t\mathbf{1}_{n_{t+1}} + \lambda_t^T\tilde{M}_t\mathbf{1}_{n_{t+1}} - \lambda_{t+1}^T\tilde{M}_t^T\mathbf{1}_{n_t} \\
& - \epsilon\mathbf{1}_{n_t}^T\tilde{M}_t\mathbf{1}_{n_{t+1}} + \epsilon\mathbf{1}_{n_t}^T\tilde{P}_t\mathbf{1}_{n_{t+1}}, \\
\epsilon D(\hat{M}_t|\hat{P}_t) =& -\langle \hat{C}_t, \hat{M}_t\rangle + \rho^T\hat{M}_t\mathbf{1}_{n_T} + \lambda_t^T\hat{M}_t\mathbf{1}_{n_T} - \lambda_T^T\hat{M}_t^T\mathbf{1}_{n_t} \\
& - \epsilon\mathbf{1}_{n_t}^T\hat{M}_t\mathbf{1}_{n_T} + \epsilon\mathbf{1}_{n_t}^T\hat{P}_t\mathbf{1}_{n_{t+1}},
\end{aligned}$$

and, for $t = T-1$,

$$\begin{aligned}
\epsilon D(\tilde{M}_{T-1}|\tilde{P}_{T-1}) =& -\langle \tilde{C}_{T-1}, \tilde{M}_{T-1}\rangle + \rho^T\tilde{M}_{T-1}\mathbf{1}_n + \lambda_{T-1}^T\tilde{M}_{T-1}\mathbf{1}_n \\
& - \epsilon\mathbf{1}_n^T\tilde{M}_{T-1}\mathbf{1}_{n_T} + \epsilon\mathbf{1}_n^T\tilde{P}_{T-1}\mathbf{1}_n, \\
\epsilon D(\hat{M}_{T-1}|\hat{P}_{T-1}) =& -\langle \hat{C}_{T-1}, \hat{M}_{T-1}\rangle + \rho^T\hat{M}_{T-1}\mathbf{1}_n + \lambda_{T-1}^T\hat{M}_{T-1}\mathbf{1}_n - \lambda_T^T\hat{M}_{T-1}^T\mathbf{1}_n \\
& - \epsilon\mathbf{1}_n^T\hat{M}_{T-1}\mathbf{1}_{n_T} + \epsilon\mathbf{1}_n^T\hat{P}_{T-1}\mathbf{1}_n.
\end{aligned}$$

Thus, when we substitute the optimal transport plans (in terms of the dual variables) into the Lagrange function, many of the terms cancel, and we obtain the following dual function (up to an additive constant):

$$\varphi(\mathbf{\Lambda}) = -\epsilon\left(\sum_{t=0}^{T-2}\langle \tilde{K}_t, \tilde{U}_t\rangle - \sum_{t=0}^{T-1}\langle \hat{K}_t, \hat{U}_t\rangle\right) + \mathbf{1}_{n_0}^T\lambda_0 + \mathbf{1}_{n_F}^T\lambda_T + \mathbf{1}_n^T\rho.$$

Alternatively, it can be expressed explicitly in terms of the dual variables $\mathbf{\Lambda}$,

$$\begin{aligned}
\varphi(\mathbf{\Lambda}) =& -\epsilon\big(e^{\lambda_0/\epsilon}\big)^T\left(\tilde{K}_0e^{-\lambda_1/\epsilon} + \hat{K}_0e^{-\lambda_T/\epsilon}\right) \\
& - \epsilon\sum_{t=1}^{T-2}\left(e^{\lambda_t/\epsilon}\odot e^{\rho/\epsilon}\right)^T\left(\tilde{K}_te^{-\lambda_{t+1}/\epsilon} + \hat{K}_te^{-\lambda_T/\epsilon}\right) \\
& - \epsilon\big(e^{\lambda_{T-1}/\epsilon}\odot e^{\rho/\epsilon}\big)^T\left(\hat{K}_{T-1}e^{-\lambda_T/\epsilon}\right) \\
& + \mathbf{1}_{n_0}^T\lambda_0 + \mathbf{1}_{n_F}^T\lambda_T + \mathbf{1}_n^T\rho.
\end{aligned}$$

Since there exists a strictly positive feasible solution, we know there exists a point in the relative interior of the feasible set (Slater's condition). Thus, by Slater's theorem, strong duality holds (see [27] Section 5.3.2. for a proof).

In the case of infinite cost elements $(\tilde{C}_t)_{ij} = \infty$ or $(\hat{C}_t)_{ij} = \infty$ for some i, j and t, the corresponding elements in $\tilde{M}_t$ or $\hat{M}_t$ need to be 0 for the transport to be feasible, and such transports may thus be removed from the formulation without changing the problem. If there is a feasible solution with all remaining variables strictly positive, then Slater's condition still holds.

□

Remark 1. *For certain choices $(\tilde{C}_t)_{ij} = \infty$ or $(\hat{C}_t)_{ij} = \infty$, the optimal solution might require setting other transport elements to 0 even though the corresponding costs are finite, meaning Slater's condition is not fulfilled; this may for example happen if an entire row or column is filled with only infinite cost elements. This situation corresponds to infinite dual variables and is avoided by the assumption that $(\tilde{M}_t)_{ij} > 0$ whenever $(\tilde{C}_t)_{ij} < \infty$, and $(\hat{M}_t)_{ij} > 0$ whenever $(\hat{C}_t)_{ij} < \infty$ in Proposition 4.1.*

4.2 Computation of optimal transport plans via block-coordinate ascent on the dual function

We solve the dual problem derived above in a block-wise fashion: each "block update" corresponds to optimizing the dual function with respect to a particular dual variable vector in each step, while keeping the others fixed. This procedure then directly leads to Algorithm 1. From the optimal dual variables, the optimal transport plans are readily obtained using the results in Proposition 4.1.

Taking the gradient with respect to ρ yields

$$\nabla_\rho \varphi = \mathbf{1}_n - e^{\rho/\epsilon} \odot \left[e^{\lambda_{T-1}/\epsilon} \odot \left(\hat{K}_{T-1} e^{-\lambda_T/\epsilon} \right) + \sum_{t=1}^{T-2} e^{\lambda_t/\epsilon} \odot \left(\tilde{K}_t e^{-\lambda_{t+1}/\epsilon} + \hat{K}_t e^{-\lambda_T/\epsilon} \right) \right],$$

or, defining $s = \exp(\rho/\epsilon)$, we can express this in terms of the utility variables:

$$\nabla_\rho \varphi = \mathbf{1}_n - s \odot \left[u_{T-1} \odot \left(\hat{K}_{T-1} v_T \right) + \sum_{t=1}^{T-2} u_t \odot \left(\tilde{K}_t v_{t+1} + \hat{K}_t v_T \right) \right].$$

Setting this to zero and solving for s leads to

$$s = \mathbf{1}_n \oslash \left[u_{T-1} \odot \left(\hat{K}_{T-1} v_T \right) + \sum_{t=1}^{T-2} u_t \odot \left(\tilde{K}_t v_{t+1} + \hat{K}_t v_T \right) \right].$$

Note however that we also have the constraints $\rho \geq 0$. To ensure that this constraint is not violated, the update rule is modified and becomes:

$$s = \max \left\{ \mathbf{1}_n, \mathbf{1}_n \oslash \left[u_{T-1} \odot \left(\hat{K}_{T-1} v_T \right) + \sum_{t=1}^{T-2} u_t \odot \left(\tilde{K}_t v_{t+1} + \hat{K}_t u_T \right) \right] \right\}. \tag{4.3}$$

Taking the gradient with respect to λ_0 yields

$$\nabla_{\lambda_0}\varphi = \mathbf{1}_{n_0} - \left(\tilde{K}_0 e^{-\lambda_1/\epsilon} + \hat{K}_0 e^{\lambda_T/\epsilon}\right) \odot e^{\lambda_0/\epsilon} = \mathbf{1}_{n_0} - (\tilde{K}_0 v_1 + \hat{K}_0 v_T) \odot u_0.$$

Again, imposing that this should be zero (and ensuring $\lambda_0 \geq 0$) we may solve for u_0 to obtain:

$$u_0 = \max\left\{\mathbf{1}_{n_0}, \mathbf{1}_{n_0} \oslash (\tilde{K}_0 v_1 + \hat{K}_0 v_T)\right\}. \tag{4.4}$$

Similarly, differentiating with respect to λ_1, one obtains

$$\begin{aligned}\nabla_{\lambda_1}\varphi &= \left(\tilde{K}_0^T e^{\lambda_0/\epsilon}\right) \odot e^{-\lambda_1/\epsilon} - \left(\tilde{K}_1 e^{-\lambda_{t+1}/\epsilon} + \hat{K}_1 e^{\lambda_T/\epsilon}\right)\left(e^{\lambda_t/\epsilon} \odot e^{\rho/\epsilon}\right) \\ &= \left(\tilde{K}_0^T u_0\right) \oslash u_1 - \left(\tilde{K}_1 v_2 + \hat{K}_1 v_T\right)\left(u_1 \odot s\right) = 0,\end{aligned}$$

and, solving for u_1,

$$u_1 = \left\{\left(\tilde{K}_0^T u_0\right) \oslash \left[s \odot \left(\tilde{K}_1 v_2 + \hat{K}_1 v_T\right)\right]\right\}^{1/2}. \tag{4.5}$$

Repeating this procedure for $t = 2, \ldots, T-2$, one obtains

$$u_t = \left\{\left(\tilde{K}_{t-1}^T (u_{t-1} \odot s)\right) \oslash \left[s \odot \left(\tilde{K}_t v_{t+1} + \hat{K}_t v_T\right)\right]\right\}^{1/2}, \tag{4.6}$$

and for the final two blocks,

$$u_{T-1} = \left\{\left(\tilde{K}_{T-2}^T (u_{T-2} \odot s)\right) \oslash \left(s \odot \left(\hat{K}_{T-1} v_T\right)\right)\right\}^{1/2} \tag{4.7}$$

$$u_T = \max\left\{\mathbf{1}_{n_F}, \mathbf{1}_{n_F} \oslash \left[\hat{K}_0^T u_0 + \sum_{t=1}^{T-1} \hat{K}_t^T (u_t \odot s)\right]\right\}. \tag{4.8}$$

The algorithm is summarized in Algorithm 1.

In practice, we define the following cost matrices:

$$\tilde{C}_0 := c(\mathcal{X}_0, \mathcal{X}) \tag{4.9}$$

$$\hat{C}_0 := [\infty]_{i=1,j=1}^{n_0,n_F} \tag{4.10}$$

$$\tilde{C}_t := c(\mathcal{X}, \mathcal{X}) + \eta I, \qquad \text{for } t = 1, \ldots, T-2 \tag{4.11}$$

$$\hat{C}_t := c(\mathcal{X}, \mathcal{X}_F), \quad \text{for } t = 1, \ldots, T-1, \tag{4.12}$$

where the cost function $c(\cdot,\cdot)$ is defined in (3.2), corresponding to the squared Euclidean distances between cell states, η is a large number which penalizes self-transports and I is the n by n identity matrix. We effectively forbid such transport by setting the diagonal to `numpy.inf` in our Python implementation. Note that our choice of $\hat{C}_0$ corresponds to an assumption that no initial states takes a terminal differentiation step in the first stage of transport.

Algorithm 1 Block-coordinate ascent algorithm for solving (3.20)

Given: A data set $\mathcal{D} = \{x^{(i)} | i = 1, \ldots, N\}$ of the cellular states (e.g., gene expression levels) of N cells partitioned into three disjoint ordered subsets $\mathcal{X}_0$ (initial states), $\mathcal{X}$ (intermediate states), $\mathcal{X}_F$ (terminal states).
Choose: parameter values for the number of time steps, T, and entropy-regularization, $\epsilon \in (0, \infty)$.
Pre-compute: matrices $\tilde{K}_t$ for $t = 0, 1, 2, \ldots, T-2$, and $\hat{K}_t$ for $t = 1, 2, \ldots, T-1$ (Note: we set all elements of $\hat{K}_0 = [(\hat{K}_0)_{ij}]_{i=1,j=1}^{n_0,n_F}$ to zero in our implementation, corresponding to infinite costs, $(\hat{C}_0)_{ij} = \infty$, $i = 1, \ldots, n_0$, $j = 1, \ldots, n_F$).
Initialize $u_t = \mathbf{1}_{n_t}$, for $t = 0, 1, \ldots, T$, and $s = \mathbf{1}_n$.

- **while** not converged **do**
  - **for** $t = 0, 1, \ldots, T$ **do**
    - $v_t \leftarrow \mathbf{1}_{n_t} \oslash u_t$
  - **end for**
  - $s \leftarrow \max\left\{\mathbf{1}_n, \mathbf{1}_n \oslash \left[u_{T-1} \odot \left(\hat{K}_{T-1} v_T\right) + \sum_{t=1}^{T-2} u_t \odot \left(\tilde{K}_t v_{t+1} + \hat{K}_t v_T\right)\right]\right\}$
  - $u_0 \leftarrow \max\left\{\mathbf{1}_{n_0}, \mathbf{1}_{n_0} \oslash \left(\tilde{K}_0 v_1 + \hat{K}_0 v_T\right)\right\}$
  - $u_1 \leftarrow \left\{\left(\tilde{K}_0^T u_0\right) \oslash \left[s \odot \left(\tilde{K}_1 v_2 + \hat{K}_1 v_T\right)\right]\right\}^{1/2}$
  - **for** $t = 2, \ldots, T-2$ **do**
    - $u_t \leftarrow \left\{\left(\tilde{K}_{t-1}^T \left(u_{t-1} \odot s\right)\right) \oslash \left[s \odot \left(\tilde{K}_t v_{t+1} + \hat{K}_t v_T\right)\right]\right\}^{1/2}$
  - **end for**
  - $u_{T-1} \leftarrow \left\{\left(\tilde{K}_{T-2}^T \left(u_{T-2} \odot s\right)\right) \oslash \left[s \odot \left(\hat{K}_{T-1} v_T\right)\right]\right\}^{1/2}$
  - $u_T \leftarrow \max\left\{\mathbf{1}_{n_F}, \mathbf{1}_{n_F} \oslash \left[\hat{K}_0^T u_0 + \sum_{t=1}^{T-1} \hat{K}_t^T \left(u_t \odot s\right)\right]\right\}$
- **end while**
- $\tilde{M}_0 \leftarrow \tilde{K}_0 \odot \left(u_0 v_1^T\right)$
- $\hat{M}_0 \leftarrow \hat{K}_0 \odot \left(u_0 v_T^T\right)$
- **for** $t = 1, \ldots, T-2$ **do**
  - $\tilde{M}_t \leftarrow \tilde{K}_t \left((u_t \odot s) v_{t+1}^T\right)$
  - $\hat{M}_t \leftarrow \hat{K}_t \left((u_t \odot s) v_T^T\right)$
- **end for**
- $\hat{M}_{T-1} \leftarrow \hat{K}_{T-1} \left((u_t \odot s) v_T^T\right)$

4.3 Reduction of entropy-regularization through a proximal point scheme

In our derivation of Algorithm 1, the regularization parameter ϵ was taken as a given constant. However, the solution indeed depends on ϵ, and setting $\epsilon = 0$ reverts the regularized problem back into the original problem (P). For this reason, it may be tempting to choose ϵ very small. However, the block-coordinate update scheme derived above is not defined for $\epsilon = 0$, as this would cause all elements in the K_t-matrices to become 0, yielding infeasible transport plans for any finite choice of the dual variables; and even for strictly positive values, choosing ϵ too small leads to numerical instability if enough elements in

the K_t-matrices become too small to be represented as non-zero values. For small $\epsilon > 0$, this issue can in principle be solved by performing all computations on the log-transformed utility variables instead [28]. However, this greatly increases the computational complexity of the algorithm [29].

To avoid having to choose ϵ too large (and thus straying too far from optimality in the original problem), we nested the block-coordinate ascent method in an outer proximal point scheme, based on the approach introduced in Xie et al. [29]. The proximal point scheme works by taking a previously computed solution as a prior in the entropy term. The solution to this new problem can be shown to correspond to a different parameter ϵ' which is strictly smaller than the parameter used for the original problem. To see this, consider $\tilde{M}_t^p$ as prior optimal transport plan in iteration p of the proximal point scheme, obtained after solving (3.20) with prior transport plan matrix $\tilde{M}_t^{p-1}$. Then, from Proposition 4.1, we have that

$$\tilde{M}_t^p = \tilde{K}_t^p \odot \tilde{U}_t^p = \tilde{M}_t^{p-1} \odot \exp\left(-\tilde{C}_t/\epsilon\right) \odot \tilde{U}_t^p. \tag{4.13}$$

In proximal point iteration $p+1$, we solve (3.20) again using entropy-regularization $D\left(\tilde{M}_t^{p+1}|\tilde{M}_t^p\right)$. The optimal transport plan can be expressed as

$$\tilde{M}_t^{p+1} = \tilde{M}_t^p \odot \exp\left(-\tilde{C}_t/\epsilon\right) \odot \tilde{U}_t^{p+1} = \tag{4.14}$$

$$= \left(\tilde{M}_t^{p-1} \odot \exp\left(-\tilde{C}_t/\epsilon\right) \odot \tilde{U}_t^p\right) \odot (-\tilde{C}_t/\epsilon) \odot \tilde{U}_t^{p+1} \tag{4.15}$$

$$= \tilde{M}_t^{p-1} \odot \exp\left(-\frac{\tilde{C}_t}{\frac{\epsilon}{2}}\right) \odot \tilde{U}_t^p \odot \tilde{U}_t^{p+1}. \tag{4.16}$$

Hence, by induction, we find that the transport plans obtained in this way in proximal point iteration p can be expressed

$$\tilde{M}_t^p = \tilde{P}_t^0 \odot \exp\left(-\frac{\tilde{C}_t}{\frac{\epsilon}{p+1}}\right) \odot \mathcal{U}_t^p, \tag{4.17}$$

where $\tilde{\mathcal{U}}_t := \tilde{U}_t^p \odot \tilde{U}_t^{p-1} \odot \tilde{U}_t^{p-2} \odot \cdots \odot \tilde{U}_t^0$, and $\tilde{P}_t^0$ is an initial prior regularization matrix (not necessarily a transport plan). This corresponds to regularization parameter $\epsilon/(p+1)$, which decreases as the number of outer iterations p increases. More generally, if, in iteration p of the proximal point scheme, (3.20) is solved using regularization parameter ϵ_p and if the prior transport plan had been obtained by solving (3.20) with regularization parameter ϵ_{p-1}, then the new optimal transport plans will correspond to regularization parameter

$$\epsilon' = \frac{1}{\frac{1}{\epsilon_{p-1}} + \frac{1}{\epsilon_p}}. \tag{4.18}$$

By induction, the effective regularization parameter after p iterations is

$$\epsilon' = \frac{1}{\frac{1}{\epsilon_p} + \frac{1}{\epsilon_{p-1}} + \cdots \frac{1}{\epsilon_0}}, \tag{4.19}$$

with ϵ_0 corresponding to the first transport plan prior obtained by solving (3.20) with some initial prior regularization matrix.

In our implementation, we initiate the proximal point scheme with the "uninformative" regularization priors $\tilde{P}_t = \mathbf{1}_{n_t}\mathbf{1}_{n_{t+1}}^T$, $t = 0, 1, \ldots, T-2$ and $\hat{P}_t = \mathbf{1}_{n_t}\mathbf{1}_{n_F}^T$, $t = 0, 1, \ldots, T-1$. In all subsequent iterations, the optimal transport plans obtained in the previous iteration are taken as regularization priors—effectively decreasing the effects of regularization on the optimal transport plans as shown above. If in any outer iteration the solutions become numerically unstable, we increase the value of the regularization parameter for that iteration. This generates a sequence of strictly decreasing effective regularization parameter values, as in (4.19), and the proximal point scheme terminates when the desired ϵ-threshold is reached. In theory, this method can be used to reduce the effects of regularization to zero, yielding an "exact" solution. However, in practice, we found the value ϵ_p in each iteration may need to grow so large that this convergence would be realized very slowly. Notwithstanding, it allowed us to reduce the regularization parameter with roughly a factor of 5 compared to the original value (see Table S1).

5 Model extension with auxiliary cell states

We noticed that when a snapshot contains cells for which there are no nearby cell states, the cost of sending mass to the nearest neighbour may be so large that it leads to numerical instability in the Sinkhorn iterations. From a biological perspective, then, it may be invalid to assume that such cells should be allowed to form connections to other cells in the data set. To simultaneously address both of these issues, we extended the standard MultistageOT model by adding three auxiliary states: an auxiliary initial (denoted $\mathcal{A}_0$), intermediate (denoted $\mathcal{A}$) and terminal (denoted $\mathcal{A}_F$) state respectively (Fig. S16). Cells in the snapshot $\mathcal{D}$ may form connections with these auxiliary cell states, if it is too costly to form connections with other cells in $\mathcal{D}$. We thus introduce a fixed cost, denoted Q, at which cells can transport mass to any of these auxiliary states.

More precisely, we add an auxiliary state to each of the groups $\mathcal{X}_0, \mathcal{X}$ and $\mathcal{X}_F$, and modify the indices accordingly, i.e.,

$$\mathcal{X}_0 \to (x^{(i)} \,|\, i = 1, \ldots, n_0 + 1) \tag{5.1}$$

$$\mathcal{X} \to (x^{(i)} \,|\, i = n_0 + 2, \ldots, N - n_F + 1) \tag{5.2}$$

$$\mathcal{X}_F \to (x^{(i)} \,|\, i = N - n_F + 2, \ldots, N + 3), \tag{5.3}$$

so that $x^{(n_0+1)}$ corresponds to the auxiliary initial state, $x^{(N-n_F+1)}$ corresponds to the auxiliary intermediate state, and $x^{(N+3)}$ corresponds to the auxiliary terminal state. We add the following costs to these auxiliary states. For $t = 0$,

$$(\tilde{C}_0)_{n_0+1}, j = Q \quad \text{for } j = 1, \ldots, N - n_F + 1 \tag{5.4}$$

$$(\hat{C}_0)_{n_0+1}, j = \begin{cases} Q & \text{for } j = n_F + 1 \\ \infty & \text{otherwise.} \end{cases} \tag{5.5}$$

For $t = 1, \dots, T-2$,

$$(\tilde{C}_t)_{N-n_0-n_F+1,j} = \begin{cases} Q & \text{for } j = 1, \dots, N-n_0-n_F \\ \infty & \text{for } j = N-n_0-n_F+1 \end{cases} \tag{5.6}$$

$$(\hat{C}_t)_{N-n_0-n_F+1,j} = \begin{cases} Q & \text{for } j = n_F+1 \\ \infty & \text{otherwise} \end{cases} \tag{5.7}$$

$$(\tilde{C}_t)_{i,N-n_0-n_F+1} = Q \quad \text{for } i = 1, \dots, N-n_0-n_F \tag{5.8}$$

$$(\hat{C}_t)_{i,n_F+1} = Q \quad \text{for } i = 1, \dots, N-n_0-n_F+1, \tag{5.9}$$

and, for $t = T-1$,

$$(\hat{C}_{T-1})_{N-n_0-n_F+1,j} = \begin{cases} Q & \text{for } j = n_F+1 \\ \infty & \text{otherwise} \end{cases} \tag{5.10}$$

$$(\hat{C}_{T-1})_{i,n_F+1} = Q \quad \text{for } i = 1, \dots, N-n_0-n_F+1. \tag{5.11}$$

Expanding the utility variables analogously, we add the following update rules in each iteration:

$$s_{N-n_F-n_0+1} \leftarrow 1 \tag{5.12}$$

$$(u_0)_{n_0+1} \leftarrow 1 \tag{5.13}$$

$$(u_T)_{n_F+1} \leftarrow 1, \tag{5.14}$$

with all other update rules remaining the same. These updates correspond to a lack of transport constraints on the auxiliary states (unlike their counterparts in the original formulation, the auxiliary initial and intermediate states are not constrained to send a certain amount of mass, and the auxiliary terminal state is not constrained to receive a certain amount of mass).

We first validated this extended model on the 2D synthetic data set, as presented in Fig. 4, but with an added island of three outlier cell states (Fig. 7A). When computing cell fate probabilities, our extended model predicted that all three outliers would end up being absorbed in the "unknown fate", represented by the auxiliary terminal fate (Fig. 7B). To gauge how the addition of the auxiliary states affected the rest of the solution, we compared the cell fate predictions made on the remaining, non-outlier cells, with the original predictions (Fig. 4I) using the total variation (TV) as a measure of the deviations. In doing so, we found that the change in the predicted cell fate probabilities corresponded to a TV $\leq 3.3 \cdot 10^{-5}$ in any cell (Fig. S8E), indicating little to no change. We then applied the extended model when no outliers were present, i.e., on the exact data used in Fig. 4. In this case, comparing the model with and without auxiliary states, we found the maximum total variation in any cell to be $< 10^{-7}$, affirming that with no outliers present, the extended model has the desired behavior of simply ignoring the auxiliary states. When applying the extended model on a partition of the barcoded scRNA-seq data set in Weinreb et al. [30], we found that the change in predicted cell fate probabilities for the day 2 cells, as a result of adding auxiliary states, was small for large enough choices of Q (see Table S3).

6 Relation to bimarginal approaches and continuous-time formulations

In this section we place MultistageOT into a broader context and compare it to existing optimal transport literature.

6.1 MultistageOT versus classical bimarginal optimal transport (StationaryOT)

Previously, trajectory inference with optimal transport in a single snapshot has been addressed using a stationary approach, exemplified by the StationaryOT algorithm [21]. We here discuss fundamental differences between their approach and our generalized multistage formulation (MultistageOT).

StationaryOT is an application of the classical bimarginal optimal transport formulation (2.1), and thus features a single transport stage. Specifically, by modelling initial cells as sources and terminal cells as sinks, it takes the two marginals μ_0 and μ_1 to represent the snapshot's cells in two time points. Zhang et al. [21] showed that if the sources and sinks are weighted appropriately, a (single) transport plan matrix can be taken as a proxy for transitions between cell states, and thus indicates a direction in the transport. While this represents an elegant solution to applying classical bimarginal optimal transport to a snapshot of single-cell data, the approach comes with limitations since it models the differentiation process using a single time step. Fundamentally, since StationaryOT employs a single transport stage—which produces a single transport plan matrix M—it is inherently unable to trace cell transitions across the differentiation process (which we know involves more than a single step). By contrast, MultistageOT formulates cell differentiation as a transportation problem over a sequence of multiple transport stages $t = 0, 1, \ldots, T-1$—each stage with its own pair of transport plans $(\tilde{M}_t, \hat{M}_t)$. By mapping each transport plan to a particular stage of the differentiation process, a multistage formulation naturally establishes a temporal order of the cells. This is not possible from the stationary formulation alone. For this reason, MultistageOT can be seen as a dynamic—as opposed to stationary—formulation able to explicitly model cell differentiation as a multistep process (see Section 6.2 for a more detailed discussion about the connection between MultistageOT and dynamic, continuous-time, optimal transport). This also opens up for developing further refinements of MultistageOT, such as incorporating known biological characteristics of the dynamics that may depend on differentiation stage or cell type. Finally, an additional contribution in MultistageOT is the introduction of the concept of auxiliary cell states, which allows us to keep track of outliers that do not belong to the process. This feature is not available in StationaryOT.

6.2 Continuous-time optimal transport formulations

In this section, we discuss the connection between continuous-time, or dynamic, formulations of optimal transport and our multistage optimal transport model of cell differentiation. Assume we are given densities μ_0, μ and μ_F representing the distributions of

initial states, intermediate states, and terminal states respectively. We here consider the following optimization problem:

$$\min_{\rho,\nu,f} \int_0^1 \int_X \frac{1}{2}\|f(t,x)\|^2 \rho(t,x)dxdt \tag{6.1}$$

$$\text{subject to } \frac{\partial \rho(t,x)}{\partial t} + \nabla \cdot (f(t,x)\rho(t,x)) = -\nu(t,x) \tag{6.2}$$

$$\int_0^1 \rho(t,x)dt = \mu(x), \quad \rho(1,x) \equiv 0 \tag{6.3}$$

$$\rho(0,x) = \mu_0(x), \quad \int_0^1 \nu(t,x)dt = \mu_F(x). \tag{6.4}$$

This problem seeks the continuous-time optimal transportation of a density $\rho(t,x)$ (on gene expression space) from an initial density $\mu_0(x)$ to a final density $\mu_F(x)$ under the influence of a velocity field $f(t,x)$ and a mass flux $\nu(t,x)$ (both also to be learnt). This can thus be seen as the continuous analogue of MultistageOT. Note that there are some important differences between this and the Benamou-Brenier formulations of optimal transport [12]. First, (6.3) requires that the evolution of ρ completely constitutes the prescribed intermediate density μ. This is a crucial difference, as the solutions to the Benamou-Brenier formulation correponds to displacement interpolation between the two densities—without constraints on how the density should behave in-between (i.e., that it should match the intermediate cell states in a single-cell snapshot). Second, in (6.2) and (6.4), mass can escape the system via the mass flux function $\nu(t,x)$ (cf. [31]), allowing the terminal density to develop mass over time (analogous to the marginals $\hat{\nu}_t$ for $t = 1, \ldots, T-1$ in MultistageOT). This addresses the fact that certain cell types may have longer trajectories than others, and it is thus not reasonable to assume a fixed time to maturation.

We emphasize that the continuous-time problem formulated in this section is not directly applicable since the data consists of a discrete set of points. MultistageOT can be viewed as an entropy-regularized discretization (both in time and in state-space) of this problem, allowing the optimal transport plans to be found via generalized Sinkhorn iterations. Consequently, if μ_0, μ and μ_F are exactly given, and we let the number of cells N and the number of transport stages T both tend to infinity, and $\epsilon \to 0$, we expect the MultistageOT problem to converge to the continuous-time problem (6.1)-(6.4). The inequality constraints used in MultistageOT reflect the fact that the densities μ_0, μ and μ_F are typically not exactly known, and that we thus allow for some freedom in the how mass should spread over the cell states, effectively treating them as latent variables. Our main findings demonstrate that these assumptions work well in practice for varying resolutions in the temporal discretization (i.e., stages in the range 11 to 26).

References

[1] Villani, C. *Topics in optimal transportation* (American Mathematical Society, 2003).

[2] Monge, G. Mémoire sur la théorie des déblais et des remblais. *Historie de l'Academie Royale des Sciences de Paris* 666–704 (1781).

[3] Hitchcock, F. L. The distribution of a product from several sources to numerous localities. *J. Math. Phys.* **20**, 224–230 (1941).

[4] Kantorovich, L. V. On the translocation of masses. In *Proc. USSR Acad. Sci.*, vol. 37, 199–201 (1942).

[5] Kantorovich, L. V. Mathematical methods of organizing and planning production. *Manage. Sci.* **6**, 366–422 (1960).

[6] Cuturi, M. Sinkhorn distances: Lightspeed computation of optimal transport. In *Adv. Neural Inf. Process. Syst.* (eds Burges, et al.) (NeurIPS, 2013).

[7] Sinkhorn, R. & Knopp, P. Concerning nonnegative matrices and doubly stochastic matrices. *Pacif. J. Math.* **21**, 343–348 (1967).

[8] Rabin, J., Ferradans, S. & Papadakis, N. Adaptive color transfer with relaxed optimal transport. In *Int. Conf. Image Process. ICIP*, 4852–4856 (IEEE, 2014).

[9] Ferradans, S., Papadakis, N., Peyré, G. & Aujol, J.-F. Regularized discrete optimal transport. *SIAM J. Imaging Sci.* **7**, 1853–1882 (2014).

[10] Elvander, F., Haasler, I., Jakobsson, A. & Karlsson, J. Multi-marginal optimal transport using partial information with applications in robust localization and sensor fusion. *Signal Process.* **171**, 107474 (2020).

[11] Haasler, I., Karlsson, J. & Ringh, A. Control and estimation of ensembles via structured optimal transport. *IEEE Control Syst. Mag.* **41**, 50–69 (2021).

[12] Benamou, J.-D. & Brenier, Y. A computational fluid mechanics solution to the Monge-Kantorovich mass transfer problem. *Numer. Math.* **84**, 375–393 (2000).

[13] Chen, Y., Georgiou, T. T. & Pavon, M. On the relation between optimal transport and Schrödinger bridges: A stochastic control viewpoint. *J. Optim. Theory Appl.* **169**, 671–691 (2016).

[14] Haasler, I., Ringh, A., Chen, Y. & Karlsson, J. Scalable computation of dynamic flow problems via multimarginal graph-structured optimal transport. *Math. Oper. Res.* (2023).

[15] Mascherpa, M., Haasler, I., Ahlgren, B. & Karlsson, J. Estimating pollution spread in water networks as a Schrödinger bridge problem with partial information. *Eur. J. Control* **74**, 100846 (2023).

[16] Sandhu, R. *et al.* Graph curvature for differentiating cancer networks. *Scientific reports* **5**, 12323 (2015).

[17] Farooq, H., Chen, Y., Georgiou, T. T., Tannenbaum, A. & Lenglet, C. Network curvature as a hallmark of brain structural connectivity. *Nature communications* **10**, 4937 (2019).

[18] Schiebinger, G. *et al.* Optimal-transport analysis of single-cell gene expression identifies developmental trajectories in reprogramming. *Cell* **176**, 928–943 (2019).

[19] Yang, K. D. *et al.* Predicting cell lineages using autoencoders and optimal transport. *PLoS Comput. Biol.* **16**, e1007828 (2020).

[20] Forrow, A. & Schiebinger, G. LineageOT is a unified framework for lineage tracing and trajectory inference. *Nat. Commun.* **12**, 4940 (2021).

[21] Zhang, S., Afanassiev, A., Greenstreet, L., Matsumoto, T. & Schiebinger, G. Optimal transport analysis reveals trajectories in steady-state systems. *PLoS Comput. Biol.* **17**, e1009466 (2021).

[22] Lamoline, F., Haasler, I., Karlsson, J., Gonçalves, J. & Aalto, A. Gene regulatory network inference from single-cell data using optimal transport. *bioRxiv* 2024–05 (2024).

[23] Peyré, G., Cuturi, M. *et al.* Computational optimal transport: With applications to data science. *Found. Trends Mach. Learn.* **11**, 355–607 (2019).

[24] Franklin, J. & Lorenz, J. On the scaling of multidimensional matrices. *Linear Algebra Appl.* **114**, 717–735 (1989).

[25] Benamou, J.-D., Carlier, G., Cuturi, M., Nenna, L. & Peyré, G. Iterative Bregman projections for regularized transportation problems. *SIAM J. Sci. Comput.* **37**, A1111–A1138 (2015).

[26] Karlsson, J. & Ringh, A. Generalized Sinkhorn iterations for regularizing inverse problems using optimal mass transport. *SIAM J. Imaging Sci.* **10**, 1935–1962 (2017).

[27] Boyd, S. & Vandenberghe, L. *Convex optimization* (Cambridge University Press, 2004).

[28] Schmitzer, B. Stabilized sparse scaling algorithms for entropy regularized transport problems. *SIAM J. Sci. Comput.* **41**, A1443–A1481 (2019).

[29] Xie, Y., Wang, X., Wang, R. & Zha, H. A fast proximal point method for computing exact Wasserstein distance. In *Uncertainty in Artificial Intelligence*, 433–453 (PMLR, 2020).

[30] Weinreb, C., Rodriguez-Fraticelli, A., Camargo, F. D. & Klein, A. M. Lineage tracing on transcriptional landscapes links state to fate during differentiation. *Science* **367**, eaaw3381 (2020).

[31] Gangbo, W., Li, W., Osher, S. & Puthawala, M. Unnormalized optimal transport. *Journal of Computational Physics* **399**, 108940 (2019).
